# RICTOR/mTORC2 downregulation in BRAF^V600E^ melanoma cells promotes resistance to BRAF/MEK inhibition

**DOI:** 10.1186/s12943-024-02010-1

**Published:** 2024-05-16

**Authors:** Luca Ponzone, Valentina Audrito, Claudia Landi, Enrico Moiso, Chiara Levra Levron, Sara Ferrua, Aurora Savino, Nicoletta Vitale, Massimiliano Gasparrini, Lidia Avalle, Lorenza Vantaggiato, Enxhi Shaba, Beatrice Tassone, Stefania Saoncella, Francesca Orso, Daniele Viavattene, Eleonora Marina, Irene Fiorilla, Giulia Burrone, Youssef Abili, Fiorella Altruda, Luca Bini, Silvia Deaglio, Paola Defilippi, Alessio Menga, Valeria Poli, Paolo Ettore Porporato, Paolo Provero, Nadia Raffaelli, Chiara Riganti, Daniela Taverna, Federica Cavallo, Enzo Calautti

**Affiliations:** 1 Molecular Biotechnology Center “Guido Tarone”, University of Turin, Turin, 10126 Italy; 2 Department of Molecular Biotechnology and Health Sciences, University of Turin, Turin, 10126 Italy; 3 Department of Science and Technological Innovation, University of Piemonte Orientale, Alessandria, 15121 Italy; 4 Functional Proteomic Section, Department of Life Sciences, University of Siena, Siena, 53100 Italy; 5 Department of Medicine, Memorial Sloan Kettering Cancer Center, New York, USA; 6 Department of Life Sciences and Systems Biology, University of Turin, Turin, 10126 Italy; 7 Department of Agriculture, Food and Environmental Sciences, Polytechnic University of Marche, Ancona, 60131 Italy; 8 Department of Personal Care, dsm-firmenich, Kaiseraugst, 4303 Switzerland; 9 Department of Clinical and Biological Sciences, University of Turin, Turin, 10124 Italy; 10 GenomeUp, Rome, 00144 Italy; 11 Department of Medical Sciences, University of Turin, Turin, 10124 Italy; 12 Neuroscience Department “Rita Levi Montalcini”, University of Turin, Turin, 10126 Italy; 13 Department of Oncology, University of Turin, Turin, 10124 Italy

**Keywords:** mTORC2, RICTOR, NAMPT, Drug resistance, Targeted therapy, BRAF^V600E^ melanoma, Mitochondrial metabolism

## Abstract

**Background:**

The main drawback of BRAF/MEK inhibitors (BRAF/MEKi)-based targeted therapy in the management of BRAF-mutated cutaneous metastatic melanoma (MM) is the development of therapeutic resistance. We aimed to assess in this context the role of mTORC2, a signaling complex defined by the presence of the essential RICTOR subunit, regarded as an oncogenic driver in several tumor types, including MM.

**Methods:**

After analyzing The Cancer Genome Atlas MM patients’ database to explore both overall survival and molecular signatures as a function of intra-tumor RICTOR levels, we investigated the effects of RICTOR downregulation in BRAF^V600E^ MM cell lines on their response to BRAF/MEKi. We performed proteomic screening to identify proteins modulated by changes in RICTOR expression, and Seahorse analysis to evaluate the effects of RICTOR depletion on mitochondrial respiration. The combination of BRAFi with drugs targeting proteins and processes emerged in the proteomic screening was carried out on RICTOR-deficient cells in vitro and in a xenograft setting in vivo.

**Results:**

Low RICTOR levels in BRAF-mutated MM correlate with a worse clinical outcome. Gene Set Enrichment Analysis of low-RICTOR tumors display gene signatures suggestive of activation of the mitochondrial Electron Transport Chain (ETC) energy production. RICTOR-deficient BRAF^V600E^ cells are intrinsically tolerant to BRAF/MEKi and anticipate the onset of resistance to BRAFi upon prolonged drug exposure. Moreover, in drug-naïve cells we observed a decline in RICTOR expression shortly after BRAFi exposure. In RICTOR-depleted cells, both mitochondrial respiration and expression of nicotinamide phosphoribosyltransferase (NAMPT) are enhanced, and their pharmacological inhibition restores sensitivity to BRAFi.

**Conclusions:**

Our work unveils an unforeseen tumor-suppressing role for mTORC2 in the early adaptation phase of BRAF^V600E^ melanoma cells to targeted therapy and identifies the NAMPT-ETC axis as a potential therapeutic vulnerability of low RICTOR tumors. Importantly, our findings indicate that the evaluation of intra-tumor RICTOR levels has a prognostic value in metastatic melanoma and may help to guide therapeutic strategies in a personalized manner.

**Supplementary Information:**

The online version contains supplementary material available at 10.1186/s12943-024-02010-1.

## Background

Malignant melanoma is the deadliest form of skin cancer, and about 50% of tumors carry activating mutations (V600E/K) of the BRAF oncogene. In the clinics, BRAF^V600E^ tumors can be selectively targeted by BRAF-inhibitors (BRAFi) administered in combination with MEK inhibitors (MEKi) [1], with remarkable clinical efficacy in the short term. However, BRAF/MEKi targeted therapy is characterized by the nearly inevitable and rapid development of therapeutic resistance [2, 3]. The mechanisms underlying resistance to BRAF/MEKi include genetic and epigenetic alterations, aberrant activation of signaling pathways, phenotype plasticity, and metabolic rewiring [3–6].The latter typically relies on a switch from a glycolytic based- to an Oxidative Phosphorylation (OXPHOS)-based energetic metabolism triggered by BRAF pharmacological inhibition [3, 4], which is often coupled with the upregulation of NAD^+^ biosynthesis [7]. In the clinics, resistance to targeted therapy may depend on the drug-resistant phenotype of preexisting tumor cell subpopulations (intrinsic resistance) or occur via *de novo* mutations that render cancer cells permanently refractory to BRAF/MEKi treatment (acquired resistance) [3, 5]. In both cases, the therapeutic pressure causes the “Darwinian” selection of drug-resistant tumor cell populations that are at the bases of the patients’ relapse. Recently, it became evident that non-genetic, reversible adaptation mechanisms occurring in tumor cells subjected to BRAF/MEKi treatment foster the maintenance of clinically elusive populations of drug-tolerant persister cells [8]. While persister cells can revert to a drug-sensitive phenotype upon discontinuation of therapeutic regimens, they can act as founders for the subsequent development of acquired genetic resistance upon continuous drug exposure. Several lines of evidence indicate that the capacity of melanoma persister cells to tolerate BRAF/MEK inhibition relies in large part on the reprogramming of the cell biosynthetic processes, including mRNA translation and/or mitochondrial energy production [8].

mTOR belongs to PI3K-related (PIKK) family of protein kinases and is a master regulator of the balance between cell biosynthetic and catabolic functions [9]. mTOR operates as the catalytic subunit of two distinct multi-protein signaling complexes, mTORC1 and mTORC2, both composed by shared (mLST8, DEPTOR) and specific components. While mTORC1 is defined by the presence of RAPTOR and PRAS40 and is activated by growth factors and amino acids, RICTOR, SIN1 and PROTOR1/2 represent the mTORC2-specific components. Both mTORC2 integrity and signaling activity depend on the essential RICTOR subunit. mTORC2 activity is typically engaged downstream of growth factors/PI3K signaling axis [9], although it can also function in a growth-factor-independent manner in specific subcellular districts [10, 11]. Through its kinase activity, mTORC2 regulates cell proliferation, survival, cytoskeleton organization, glucose and lipid metabolism by participating in the activation and/or stabilization of protein kinases such as AKT, SGK1 and PKCα [9, 12].

mTORC2 signaling is mostly regarded as an oncogenic driver in several cancer types including melanoma, via both AKT-dependent and -independent mechanisms [13, 14]. However, mTORC2 signaling can also play tumor-suppressive roles in a context-dependent manner [15, 16], and mTORC2 deficiency can protect against some forms of cellular stress. For instance, we previously found that RICTOR deficiency causes a rewiring of the energetic metabolism in murine keratinocytes that enhances the tolerance of cells towards anthracyclines and X-ray radiation [17].

Notably, the metabolic phenotype found in RICTOR-knockout keratinocytes, consisting of a switch from a glycolytic- to an OXPHOS-based energetic metabolism fueled by glutamine consumption, is reminiscent of that of targeted therapy-resistant melanoma cells. Consistently, RICTOR deficiency was previously associated with the induction of genes and/or cellular processes that favor mitochondrial ATP synthesis [18–21]. Based on these notions, we have hypothesized that RICTOR/mTORC2 deficiency in BRAF^V600E^ melanoma cells may favor BRAF/MEKi resistance.

This hypothesis prompted us to investigate this issue in the clinically relevant Skin Cutaneous Melanoma (SKCM) dataset from The Cancer Genome Atlas (TCGA) database. Our analysis revealed a correlation between low RICTOR expression in BRAF^V600E^ tumors and poor survival, accompanied by gene signatures associated with mitochondrial ATP production. Here we show that RICTOR downregulation enhances the intrinsic tolerance of drug-naïve cells to BRAF/MEK inhibition and promotes a BRAFi-resistant phenotype both in vitro and in vivo. Mechanistically, this drug resistance depends in large part on a gain in the activity of the rate-limiting enzyme of the NAD^+^ salvage pathway Nicotinamide Phosphoribosyl Transferase (NAMPT) that fuels mitochondrial OXPHOS, and that was previously identified as a driver of melanoma targeted therapy resistance [22, 23], but never shown to be negatively regulated by mTORC2. Indeed, pharmacological inhibition of either NAMPT or the mitochondrial Electron Transport Chain (ETC), administered in combination with BRAFi, restores the responsiveness of RICTOR-deficient-cells to the drug, suggesting that the NAMPT/ETC axis likely represents a specific therapeutic vulnerability of mTORC2-deficient melanomas. Our data support a model in which RICTOR/mTORC2 downregulation promotes early adaptation of BRAF^V600E^ cells to targeted therapy, accelerating the acquisition of therapeutic resistance.

## Methods

A complete list of the Methods can be found in Supplementary Materials and Methods section.

### Cell culture

A375, M14 (MDA-MB-435 S) and SK-MEL-28 cells were obtained from the American Type Culture Collection (ATCC) and maintained in Dulbecco’s modified Eagle’s medium (DMEM, Gibco 10566016) supplemented with 10% (v/v) fetal bovine serum (FBS, Gibco 10270106), 1% MEM Vitamin Solution (Gibco, 11120037), 1% MEM Non-Essential Amino Acids Solution (Gibco, 11140035), 10 mM HEPES Buffer Solution (Gibco, 15630056) and 1% Penicillin-Streptomycin (Gibco, 15140122). Cells were maintained in these culture conditions for all experiments except where specifically indicated. All cell lines were authenticated by PCR-single-locus-technology by Eurofins Genomics (Ebersberg, DE) and were routinely tested for Mycoplasma contamination.

### Colony forming efficiency (CFE) assay

Cells were seeded at 500 cells/well in 6-well plates and treated with the indicated drugs the following day. Media with the drugs was refreshed every 72 h and cells were cultured for 12 days. At the endpoint cells were fixed with 4% paraformaldehyde for 15 min, rinsed with PBS and stained with 0.1% crystal violet. Staining intensity was quantified by dissolving crystal violet with 1 ml of 10% acetic acid for 15 min, then 100 µl were moved in 96-well plate and optical density (OD) was measured by 560 nm absorbance using Promega GloMax Explorer GM3500.

For CFE assay of siRNA-transfected cells, cells were seeded at 2000 cells/well 24 h after transfection and treated with the indicated drugs the following day. Media with the drugs was refreshed every 72 h and cells were cultured for 7 days. Staining and intensity quantification were performed as described above.

### BRAFi resistance acquisition assay

Parental BRAFi-sensitive cells were seeded in 60 mm cell culture dishes (1.5 × 10^5^ cells/dish) and after 24 h treated with 0.2 µM Vemurafenib, then maintained in the presence of the same drug concentration with media refresh every 72 h. When cells reached confluency, they were collected and re-plated in new 60 mm dishes (1.5 × 10^5^ cells/dish). Cells were then treated with a higher dose of Vemurafenib and the same process was repeated when they reached confluency in the presence of the new Vemurafenib dose. Vemurafenib doses used for this experiment are indicated in Fig. 2B (0.2–0.4–0.8–1.2–1.6 µM) and cells were considered fully resistant when they could grow in the presence of 1.6 µM Vemurafenib.

### In vivo experiments

6–8 weeks-old NOD/SCID/IL2Rγ^null^ (NSG) mice were purchased from Charles River Laboratories International (Wilmington, MA, USA) and were maintained in a specific-pathogen-free (SPF) facility at the Molecular Biotechnology Center (MBC, Univerisity of Turin, Italy). 5 × 10^6^ M14 cells were resuspended in Matrigel® (Corning) and subcutaneously injected in the flank of mice, when tumors became palpable mice were treated with FK866 administered intraperitoneally (50 µl/twice daily/14 days at 20 mg/kg) and/or Vemurafenib administered by gavage (200 µl/daily/14 days at 60 mg/kg). The control group was treated in the same way administering a solution without the drug. The tumor size was measured weekly using calipers in two dimensions to generate a tumor volume using the following formula: 0.5 × (length × width^2^). After 14 days from the start of the treatment mice were euthanized and tumors were collected and weighed. Procedures were conducted in conformity with national and international laws and policies as approved by the Faculty Ethical Committee and the Italian Ministry of Health.

### Statistical analysis

For statistical analyses, significance was tested with one-way ANOVA and two-way ANOVA, with Dunnett’s, Sidak’s or Tukey’s *post hoc* tests. Statistical analysis was performed using the GraphPad Prism v8 software. *p* < 0.05 was considered significant. The definition of center and of dispersion and precision measures (e.g., mean and SD), as well as the number of technical or biological replicates of the experiments described and the specific statistical test used, are reported in the corresponding figure legends.

## Results

### Low levels of RICTOR in metastatic melanoma correlate with a poor patients’ prognosis

**Fig. 1:**
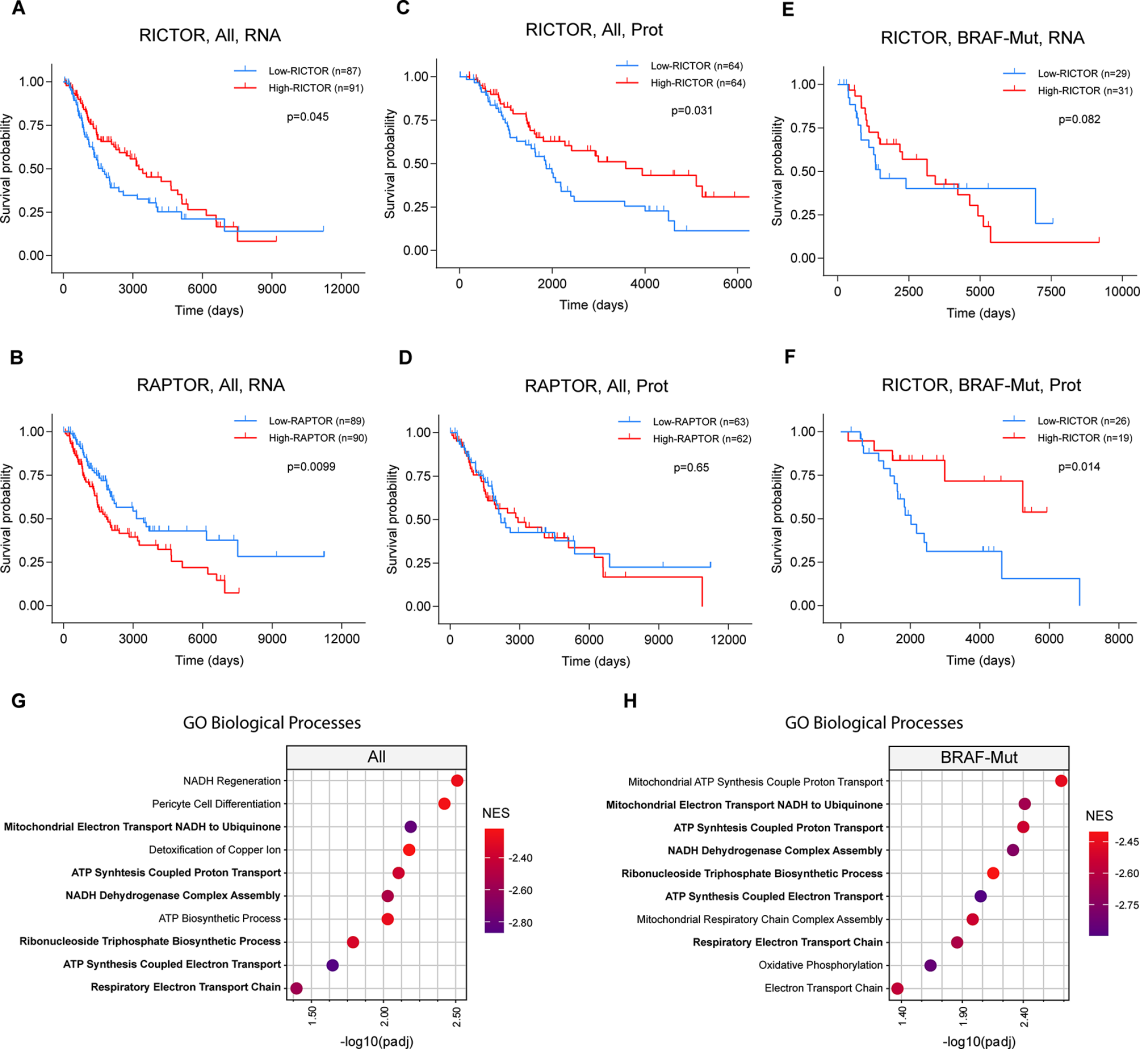
Analysis of Melanoma patients’ data from the TCGA database. (**A-F**) Kaplan-Meier survival analysis of the metastatic Skin Cutaneous Melanoma (SKCM) dataset obtained from TCGA patients’ database. **A-D** curves were obtained from analysis of the whole TCGA database irrespective of BRAF mutational status (All); **E-F** curves include only patients with reported BRAF Hotspot Mutations (BRAF-Mut); **A, B, E** curves were obtained from gene expression database (RNA); **C, D, F** were obtained from Reverse Phase Protein Array (RPPA) data. Patients were stratified into High and Low RICTOR-expressing groups based on average RNA/Protein expression levels. High-RICTOR/RAPTOR = fourth quartile; Low-RICTOR/RAPTOR = first quartile. Number of patients for each group and p-value calculated by Log-rank test are indicated in individual graphs. (**G-H**) Dotplots indicate the top 10 most significantly enriched Gene Ontology (GO) categories anticorrelated with RICTOR expression, in the (**G**) whole dataset (All; *n* = 367) or (**H**) after filtering for BRAF Hotspot Mutations (BRAF-Mut; *n* = 118). NES = Normalized Enrichment Score

To investigate the relationships between RICTOR expression and melanoma patients’ survival, we interrogated the entire Skin Cutaneous Melanoma (SKCM) cohort of patients from the publicly available The Cancer Genome Atlas (TCGA) database with respect to overall survival (OS). We found that high RICTOR mRNA levels in metastatic tumors (367 out of 448 total samples) positively correlate with patients’ survival, as indicated by a Kaplan-Meier curve obtained from patients falling in the first (*n* = 87) and fourth (*n* = 91) quartile of RICTOR expression (Fig. 1A). This was also indicated by Cox regression analysis (*p* = 0.007, median Hazard ratio = 0.68), using RICTOR expression as a continuous independent variable without imposing arbitrary thresholds (Fig S1A). In this analysis, however no significant association with RICTOR mRNA expression and distinct genetic subtypes (BRAF-Mut, NF1-Mut, RAS-Mut, Triple WT) emerged (Fig S1A). By contrast, analysis of the same cohort of patients stratified according to RAPTOR expression (first quartile *n* = 89; fourth quartile *n* = 90), indicated that elevated levels of this essential mTORC1 component correlate with a shorter OS (Fig. 1B), whereas no correlation was found between MTOR levels and patients’ survival (Fig S1B). Also for RAPTOR expression, Cox analysis confirms its negative association with patients’ survival without indicating association to specific genetic subtypes (Fig S1C). Thus, based on RICTOR and RAPTOR mRNA levels, these data suggest that mTORC2 and mTORC1 may play opposite roles in melanoma progression and/or therapeutic responses. Importantly, the positive correlation between RICTOR expression and patients’ OS was also indicated by both Kaplan-Meyer (Fig. 1C) and Cox analyses (*p* = 0.003, median Hazard ratio = 0.54) based on the TCGA protein dataset (Fig S1D), while no significant association with survival was found with the levels of RAPTOR and MTOR (Fig. 1D, S1E). Kaplan-Meier analysis performed on BRAF-mutated (BRAF-Mut) tumors did not evidence significant correlations between survival and RICTOR mRNA levels. However, both Kaplan-Meier and Cox analyses based on the TCGA protein expression dataset indicated significantly reduced survival in patients bearing BRAF-Mut tumors with low RICTOR expression (Fig. 1E, S1D). No significant difference in RICTOR mRNA or protein expression was detected between BRAF-WT (*n* = 99) and -Mut (*n* = 93) tumors, and further analysis indicated that RICTOR mRNA is only moderately correlated (Pearson correlation = 0.384 and 0.335 respectively) with protein levels, and thus may not reliably reflect the corresponding protein levels (Fig S1F, G).

**Fig. 2:**
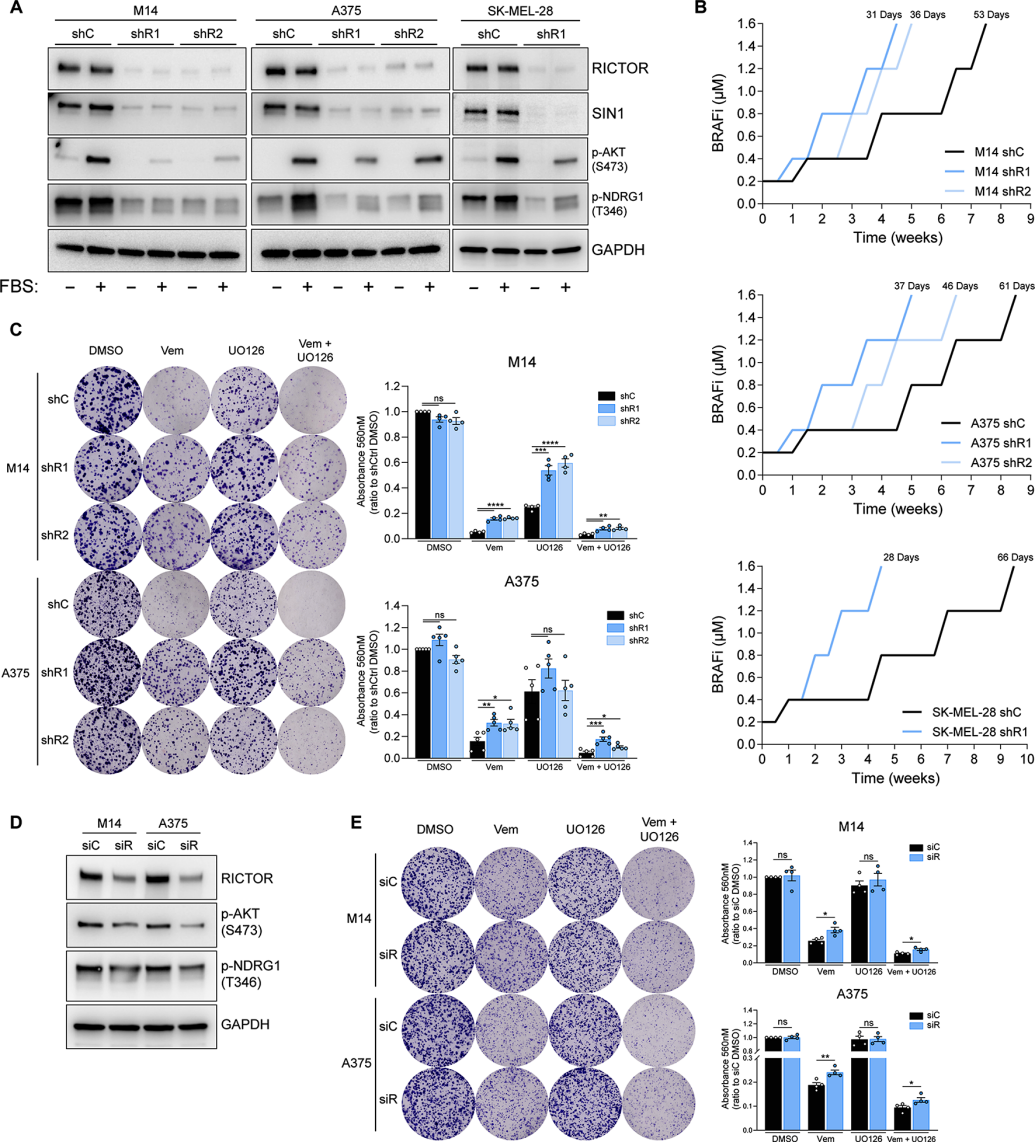
Downregulation of RICTOR in BRAF^V600E^ melanoma cell lines promotes resistance to BRAF/MEKi. (**A**) Western blot analysis of indicated cell lines transduced with RICTOR-targeting shRNAs (shR1, shR2) or scramble control (shC) lentiviruses. Cells were analyzed after 24 h of serum starvation (-) or 24 h of serum starvation followed by 15 min of refeeding (+). (**B**) Resistance acquisition kinetics analysis of RICTOR-silenced (shR1, shR2) or control (shC) M14/A375/SK-MEL-28 cells exposed to increasing doses of BRAFi (Vemurafenib, from 0.2 to 1.6 µM). The number of days required to reach resistance to 1.6 µM Vemurafenib is indicated on top of each curve. (**C**) Colony Formation Efficiency (CFE) assay of indicated cell lines cultured for 12 days in presence of vehicle control (DMSO), 0.5 µM Vemurafenib (Vem), 0.5 µM UO126 (UO126) or the combination of 0.5 µM Vemurafenib + 0.5 µM UO126 (Vem + UO126). Bar graphs represent the mean values of independent experiments ± SEM (*n* = 4 for M14 cells; *n* = 5 for A375 cells). ns = not significant, *p < 0.05, **p < 0.01, ***p < 0.001, ****p < 0.0001, one-way ANOVA followed by Dunnett’s multiple comparisons test. (**D**) Western blot analysis of indicated cell lines transfected with a RICTOR-targeting (siR) or Non-targeting control (siC) siRNAs at 72 h after transfection. (**E**) CFE assay of indicated cell lines cultured for 7 days in presence of DMSO, Vem, UO126 or the combination of Vem + UO126 as in (**C**). Bar graphs represent the mean values of 4 independent experiments ± SEM. ns = not significant, *p < 0.05, **p < 0.01, unpaired t test

Gene Set Enrichment Analysis (GSEA) revealed a significant anticorrelation between RICTOR expression levels and signatures relative to mitochondrial processes (e.g. Respiratory Electron transport chain; ATP Synthesis Coupled Proton Transport), coupled with stress protective and cell detoxifying pathways (Supplementary Table S1), both in the entire melanoma dataset as well as in the BRAF-Mut tumor subgroup (Fig. 2G, H). Thus, low expression of RICTOR in metastatic melanoma is associated with a poor clinical outcome, and the gene expression signature of low RICTOR tumors indicates the activation of processes that are frequently associated with BRAF/MEKi resistance [6, 24, 25].

### Downregulation of RICTOR in BRAF^V600E^ melanoma cell lines promotes resistance to BRAF/MEKi

To establish whether RICTOR/mTORC2 downregulation affects the responses of melanoma cells to BRAFi-based targeted therapy, we stably silenced RICTOR in three BRAF-mutated human melanoma cell lines (M14, A375 and SK-MEL-28) via lentiviral delivery of two separate RICTOR-targeted shRNAs (shR1, shR2), and compared their effects with those of a scramble control shRNA (shC). Both RICTOR protein and mRNA (Fig. 2A, S2A) levels were reduced, as well as the expression level of the other essential mTORC2 protein component SIN1 [26]. As readout of mTORC2 signaling activity, we monitored the phosphorylation levels of the downstream targets AKT and NDRG1 both under serum-deprived and -stimulated conditions (Fig. 1A). This analysis indicates that all the RICTOR-silenced cell lines display disruption of mTORC2 integrity and attenuation of downstream signaling, with no significant effects on proliferation rates under basal culture conditions (Fig S2B). Therefore, we used these cell lines as experimental models to investigate the effects of RICTOR/mTORC2 depletion in the response of BRAF-mutated melanoma cells to BRAF/MEKi.

Culture of BRAF-mutated melanoma cells in the presence of increasing concentrations of BRAFi can be used to generate BRAFi-resistant cell line variants from BRAFi-sensitive parental cells [5, 22]. We applied this procedure to evaluate the kinetics of acquisition of BRAFi-resistance in RICTOR-silenced and control cells of A375, M14 and SK-MEL-28 background over the course of ~ 8–9 weeks. We have arbitrarily set the experimental endpoint as the capacity of cells to expand in the presence of 1.6 µM Vemurafenib. Analysis of growth profiles of cell cultures in the presence of increasing drug concentrations showed that in all cellular backgrounds RICTOR-depleted cells anticipate by several weeks the reach of the experimental endpoint (Fig. 1B). Interestingly, A375 shR1 acquired the resistance status earlier than shR2 variants, suggesting that within the same cellular background RICTOR levels correlate with the timing of resistance acquisition.

To evaluate whether this anticipated acquisition of BRAFi resistance relies on an intrinsically higher tolerance to BRAFi of RICTOR-depleted cells, we have carried out Colony Formation Efficiency (CFE) survival assays by exposing M14 and A375 cells to fixed doses of BRAFi (Vemurafenib) and/or MEKi (UO126) over the course of 12 days (Fig. 2C). While RICTOR downregulation did not affect the basal clonogenicity of cells, the results indicated that RICTOR-depleted cells are intrinsically more tolerant than RICTOR-proficient counterparts to BRAFi and their combination with MEKi, as also confirmed by its acute siRNA-mediated downregulation (Fig. 2D, E).

### RICTOR protein downregulation occurs in drug-naïve BRAF^V600E^ melanoma cells as a consequence of MAPK pathway inhibition

**Fig. 3:**
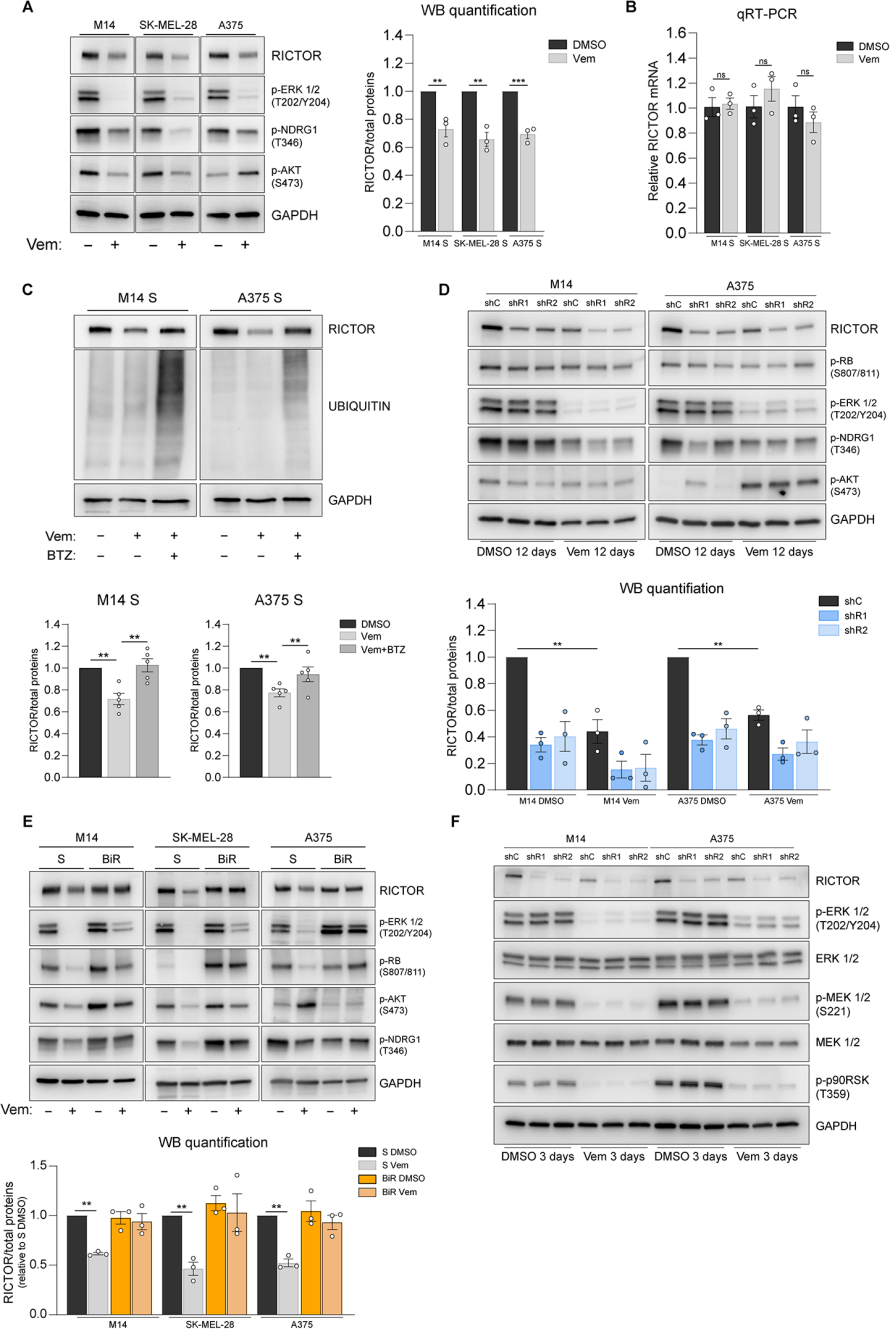
RICTOR protein downregulation occurs in drug-naïve BRAF^V600E^ melanoma cells as a consequence of MAPK pathway inhibition. (**A**) Western blot analysis of indicated cell lines treated with (-) DMSO or (+) with 1.6 µM Vemurafenib (Vem) for 72 h. Images are representative of 3 independent experiments. See *right panel* for quantification. (**B**) Quantitative reverse transcription PCR (qRT-PCR) analysis of RICTOR gene expression in the indicated cell lines treated for 72 h either with DMSO or 1.6 µM Vemurafenib (Vem). Bar graphs represent the mean values of 3 independent experiments ± SEM. ns = not significant, one-way ANOVA followed by Dunnett’s multiple comparisons test. (**C**) Upper panel: western blot analysis of indicated cell lines treated for 48 h with 1.6 µM Vem ± 10 nM Bortezomib (BTZ). Lower panel: bar graphs show the mean values of densitometric analysis of RICTOR from 5 independent experiments ± SEM. **p < 0.01, one-way ANOVA followed by Dunnett’s multiple comparisons test. (**D**) Western blot analysis of indicated cell lines. Cells were cultured for 12 days in presence of either DMSO or 0.5 µM Vem. (**E**) Western blot analysis of Vemurafenib-sensitive (S) and -resistant (BiR) BRAF^V600E^ melanoma cell lines treated for 72 h with (-) DMSO vehicle or (+) 1.6 µM Vemurafenib (Vem) (**F**) Western blot analysis of indicated cell lines treated for 72 h either with DMSO or 0.5 µM Vem

Because RICTOR levels affect the kinetics of acquisition of BRAFi resistance, it was important to determine whether the endogenous levels of the protein could be modulated in response to BRAF inhibition. Indeed, in drug-naïve M14, A375 and SK-MEL-28 cells the levels of RICTOR were significantly reduced by Vemurafenib treatment, paralleled by attenuated phosphorylation of the mTORC2 downstream target NDRG1 (Fig. 3A). Vemurafenib-induced decrease in RICTOR protein levels were not matched by a parallel decrease in mRNA, suggesting a post-transcriptional mechanism of regulation (Fig. 3B). Accordingly, concomitant treatment with proteasome inhibitor Bortezomib prevented BRAFi-induced RICTOR downregulation (Fig. 3C). In M14 and SK-MEL-28 cells AKT phosphorylation at Ser473 decreased in parallel with RICTOR, whereas in A375 cells Vemurafenib exposure increased AKT phosphorylation. This uncoupling between RICTOR and phospho-AKT levels is consistent with previous evidence of alternative, mTORC2-independent mechanisms of AKT Hydrophobic motif phosphorylation [27, 28], reported also to occur in BRAF-mutated melanoma cells, including A375 [27].

As RICTOR-silenced cells display an advantage in clonogenic growth upon BRAF inhibition, we investigated how survival/proliferation pathways are modulated under these conditions as a function of RICTOR expression and BRAF inhibition. Visual inspection of cultures kept in presence of BRAFi over the course of 12 days confirmed that colonies that survive treatment were progressively expanding, as also evidenced by similar levels of phospho-RB proliferation marker between treated and untreated colonies (Fig. 3D).

Consistent with previous data, in drug naïve RICTOR-proficient cells the endogenous levels of RICTOR protein were significantly reduced in the presence of Vemurafenib. Although the colonies generated by RICTOR-deficient cells were more abundant (Fig. 2C) and larger than those generated by control counterparts, we did not detect significant differences in the levels of ERK, AKT or RB phosphorylation that could account for their drug-tolerant phenotype (Fig. 3D). These data suggest that RICTOR downregulation occurs during the initial response and early adaptation phase to BRAFi, that normally precede the development of further mechanisms typical of acquired drug resistance.

To understand the role of RICTOR in later stages of BRAFi-resistance acquisition we applied the same methodology shown in Fig. 2B to derive BRAFi-resistant (BiR) cells from parental, drug-naïve cell lines (S). BiR cells present features of acquired resistance, such as maintenance of the resistant phenotype after drug withdrawal (Fig S3A) and reduced inhibition of ERK and RB phosphorylation upon Vemurafenib exposure, as compared to S counterparts (Fig. 3E, S3B). Moreover, BiR cells also showed a lineage-specific pattern of AKT phosphorylation under basal and treated conditions while in all BiR lineages RICTOR and p-NDRG1 levels were no longer reduced by Vemurafenib exposure (Fig. 3E, S3C). Overall, these data indicate that RICTOR expression is positively regulated by MAPK signaling both in sensitive and resistant cells.

We then attempted to define the effects of RICTOR knockdown after the acquisition of BRAFi resistance. In all three BiR lineages, shRNA-mediated RICTOR silencing significantly impaired mTORC2 downstream signaling (Fig S3D) and the clonogenic growth of cells (Fig S3E). However, in M14 and SK-MEL-28 lineages, RICTOR knockdown led to a relative increase in clonogenic growth after treatment with BRAFi, MEKi and their combination, compared to untreated conditions. Conversely, A375 RICTOR-deficient BiR cells displayed an overall reduction in the resistance to all treatments. This underscores a context-dependent role of RICTOR after development of acquired resistance, while RICTOR knockdown in drug-naïve cells consistently promotes a faster progression towards resistance in our cellular models.

To shed light on the mechanisms in which RICTOR downregulation plays a role in the early adaptation of cells to BRAFi, we analyzed pathways previously implicated in BRAF/MEKi resistance in multiple melanoma cell lines. These include receptor tyrosine kinase AXL [29], transcriptional regulators (MITF, SOX10 [30–32]) and BRAF downstream effectors (ERK, MEK, p-p90-RSK, RB) as readout of MAPK signaling activation (Fig. 3F, S3F).

Our analysis revealed that changes in RICTOR expression levels, either Vemurafenib- or shRNA-induced, did not correlate with modulation of MAPK signaling outputs nor with univocal changes in expression of the other resistance determinants we analyzed (Fig. 3F, S3F). These data did not provide sufficient evidence of the involvement of such mechanisms in the BRAFi tolerance of RICTOR-deficient cells.

### Increased mitochondrial respiration underlies the BRAFi resistance of RICTOR-deficient melanoma cells

To identify mTORC2-regulated molecules potentially implicated in the response of melanoma cells to targeted therapy, we have undertaken an unbiased proteomic approach to compare differentially expressed proteins between RICTOR-proficient and -deficient M14 and A375 cells under basal conditions. To this aim, protein extracts were separated by 2D-Gel Electrophoresis and spots were visualized by silver staining. Differentially expressed protein species were then identified by MALDI Mass Spectrometry (MS) and proteins contained in different spots displaying a fold change equal or higher than 1.5 are shown in Tables 1 and 2.

**Table 1:** **Differentially abundant proteins identified in RICTOR-deficient M14 cells by proteomic analysis**. Column 1 reports the spot numbers corresponding to those indicated in the representative image of Figure S6. Protein names of the identified spots by MALDI-ToF MS, corresponding UniProt gene names, fold change expressed as the ratio between M14 shR1 and shC (means of the Volumes of single spots/Volume of total spots quantified by densitometric analysis), and the p-value determined by one-way ANOVA Test (*n* = 5) are indicated

Spot n°	Protein name	Gene name	Fold change (shR/shC)	p-value
1	Syntenin-1	SDCBP	1.64	0.0010
2	3-hydroxyacyl-CoA dehydrogenase type-2	HSD17B10	1.97	0.0011
3	Nicotinamide phosphoribosyltransferase	NAMPT	1.54	0.0014
4	Peroxiredoxin-5, mitochondrial	PRDX5	1.59	0.0025
5	Nicotinamide phosphoribosyltransferase	NAMPT	1.87	0.0027
6	Glutamine amidotransferase-like class 1 domain-containing protein 3, mitochondrial	GATD3	1.55	0.0028
7	Histone H2A type 1-H	H2AC12	1.67	0.0031
8	Triosephosphate isomerase	TPI1	1.80	0.0040
9	Pyruvate dehydrogenase E1 component subunit alpha, somatic form, mitochondrial	PDHA1	2.58	0.0044
10	UBX domain-containing protein 1 C-term fragment	UBXN6	2.69	0.0049
11	Transgelin-2	TAGLN2	1.83	0.0052
12	High mobility group protein B1	HMGB1	1.52	0.0070
13	Poly(rC)-binding protein 1	PCBP1	2.66	0.0072
14	Pirin	PIR	1.50	0.0097
15	Small ribosomal subunit protein eS12	RPS12	1.59	0.0104
16	Polyubiquitin-B [free Ubiquitin]	UBB	1.67	0.0104
17	Phosphoserine phosphatase	PSPH	2.77	0.0110
18	Triosephosphate isomerase	TPI1	1.77	0.0124
19	Superoxide dismutase [Mn], mitochondrial	SOD2	1.73	0.0124
20	Nicotinate phosphoribosyltransferase	NAPRT	1.56	0.0125
21	Prelamin-A/C	LMNA	-1.73	0.0132
22	F-box only protein 22	FBXO22	2.90	0.0158
23	Cofilin-1	CFL1	1.51	0.0173
24	Cold shock domain-containing protein E1	CSDE1	-1.56	0.0174
25	Aldo-keto reductase family 1 member A1	AKR1A1	1.53	0.0182
26	Annexin A11	ANXA11	2.48	0.0188
27	Sialic acid synthase	NANS	1.70	0.0204
28	Far upstream element-binding protein 1	FUBP1	-2.81	0.0216
29	Translation initiation factor eIF-2B subunit alpha	EIF2B1	1.54	0.0243
30	Proteasome subunit alpha type-2	PSMA2	1.58	0.0250
31	Endoplasmic reticulum resident protein 44	ERP44	1.60	0.0258
32	NADH-ubiquinone oxidoreductase 75 kDa subunit, mitochondrial	NDUFS1	4.17	0.0260
33	Tubulin alpha-1C chain	TUBA1C	1.51	0.0272
	Peptidyl-prolyl cis-trans isomerase FKBP4	FKBP4		
34	CCHC-type zinc finger nucleic acid binding protein	CNBP	2.09	0.0314
35	Scinderin	SCIN	1.54	0.0344
36	Protein S100-A4	S100A4	-3.33	0.0366
37	Alpha-centractin	ACTR1A	1.62	0.0391
	Elongation factor Tu, mitochondrial	TUFM		
38	Inosine-5'-monophosphate dehydrogenase 2	IMPDH2	1.56	0.0393
	Adenylyl cyclase-associated protein 1	CAP1		
39	Galectin-3	LGALS3	1.60	0.0451
40	MYG1 exonuclease	MYG1	2.31	0.0494

**Table 2:** **Differentially abundant proteins identified in RICTOR-deficient A375 cells by proteomic analysis**. Protein names of the identified spots by MALDI-ToF MS, corresponding UniProt gene names, fold change expressed as the ratio between A375 shR1 and shC (means of the Volumes of single spots/Volume of total spots quantified by densitometric analysis), and the p-value determined by one-way ANOVA Test (*n* = 5) are indicated

Spot n°	Protein name	Gene Name	Fold change (shR/shC)	p-value
1	Peptidyl-prolyl cis-trans isomerase A	PPIA	-2.29	0.0101
2	Histone H2B type 1-B	H2BC3	-2.25	0.0253
3	Hippocalcin-like protein 1	HPCAL1	-3.97	0.0342
4	GTP-binding nuclear protein Ran	RAN	2.69	0.0032
5	Eukaryotic translation initiation factor 6	EIF6	-2.84	0.0138
6	Proteasome subunit alpha type-3	PSMA3	-3.35	0.0034
7	6-phosphogluconolactonase	PGLS	-2.42	0.0062
8	Proteasome activator complex subunit 3	PSME3	-2.24	0.0217
9	Serine/arginine-rich splicing factor 1	SRSF1	-2.67	0.0120
10	Tubulin beta chain	TUBB	3.66	0.0055
11	Cathepsin Z	CTSZ	-3.50	0.0001
12	Serine-threonine kinase receptor-associated protein	STRAP	12.03	0.0215
13	Twinfilin-2	TWF2	-2.89	0.0029
14	Actin, cytoplasmic 1	ACTB	-2.63	0.0223
15	Ubiquilin-1	UBQLN1	-2.34	0.0016
16	Glycine–tRNA ligase	GARS1	-2.14	0.0370
17	Eukaryotic translation initiation factor 4B	EIF4B	-2.61	0.0062
18	Eukaryotic translation initiation factor 4B	EIF4B	-2.43	0.0008
19	Far upstream element-binding protein 2	KHSRP	3.81	0.0021
20	Elongation factor 2	EEF2	2.61	0.0222
21	Glycogen phosphorylase, brain form	PYGB	2.09	0.0336
	Elongation factor 2	EEF2		
22	Vinculin	VCL	2.14	0.0061
23	Vinculin	VCL	2.55	0.0013
24	Protein S100-A10	S100A10	-1.66	0.0106
25	Peptidyl-prolyl cis-trans isomerase A	PPIA	1.55	0.0281
26	Eukaryotic translation initiation factor 5A-1	EIF5A	-1.61	0.0022
27	Chromobox protein homolog 3	CBX3	-1.58	0.0222
28	Prohibitin 1	PHB1	-1.58	0.0094
29	Calpain small subunit 1	CAPNS1	-1.61	0.0368
30	Isopentenyl-diphosphate Delta-isomerase 1	IDI1	1.63	0.0053
31	26S proteasome non-ATPase regulatory subunit 14	PSMD14	1.46	0.0236
32	Large ribosomal subunit protein uL10	RPLP0	1.47	0.0107
33	Crk-like protein	CRKL	-1.64	0.0381
34	Elongation factor Tu, mitochondrial	TUFM	1.53	0.0332
35	Anamorsin	CIAPIN1	-1.56	0.0375
36	26S proteasome non-ATPase regulatory subunit 13	PSMD13	-1.98	0.0006
37	Leukocyte elastase inhibitor	SERPINB1	-1.80	0.0015
38	Medium-chain specific acyl-CoA dehydrogenase, mitochondrial	ACADM	1.73	0.0049
39	Elongation factor Tu, mitochondrial	TUFM	1.56	0.0008
	Isocitrate dehydrogenase [NADP] cytoplasmic	IDH1		
40	Succinate–CoA ligase [ADP-forming] subunit beta, mitochondrial	SUCLA2	1.50	0.0117
41	Ornithine aminotransferase, mitochondrial	OAT	1.63	0.0068
42	Fascin	FSCN1	-1.90	0.0422
43	S-adenosylmethionine synthase isoform type-2	MAT2A	-2.00	0.0145
44	Aldehyde dehydrogenase X, mitochondrial	ALDH1B1	1.51	0.0007
45	Ras GTPase-activating protein-binding protein 1	G3BP1	1.51	0.0009
46	Prelamin-A/C	LMNA	-1.86	0.0025
47	Sorting nexin-9	SNX9	1.70	0.0143
48	Glycogen phosphorylase, brain form	PYGB	1.87	0.0101
	Elongation factor 2	EEF2		
	Cytoplasmic aconitate hydratase	ACO1		
49	Nucleolin	NCL	-1.54	0.0019
50	Heterogeneous nuclear ribonucleoprotein U-like protein 2	HNRNPUL2	-1.97	0.0092
51	Talin-1	TLN1	1.65	0.0253

**Fig. 4:**
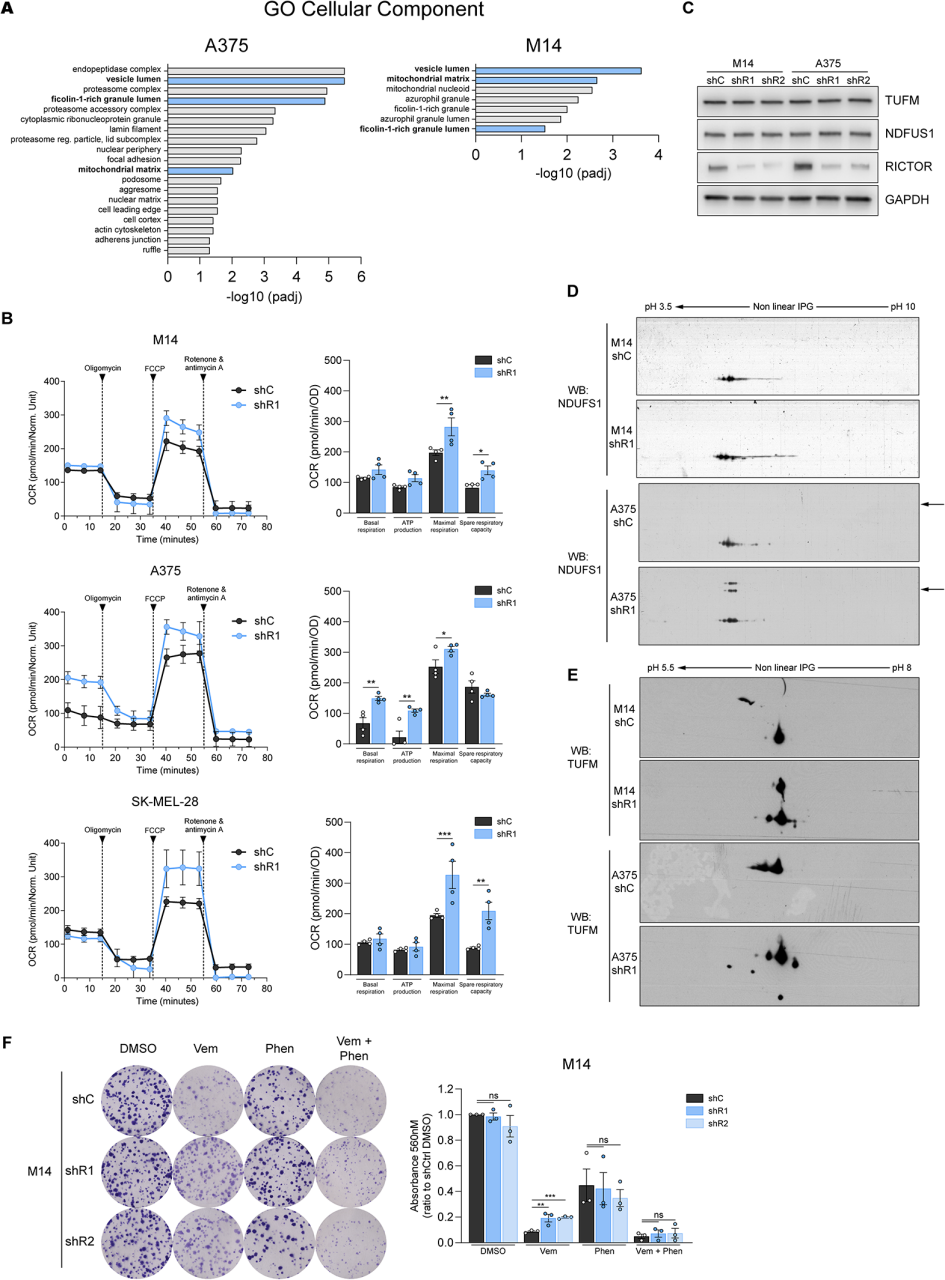
RICTOR depletion in BRAF^V600E^ melanoma cells induces alterations in mitochondrial functions and protein profiles. (**A**) Significantly enriched gene ontology (GO) categories of differentially expressed protein species identified by MALDI-ToF MS in shR1 M14 and A375 cells. Blue bars indicate GO terms in common between the two lineages. Redundant enriched terms relative to identical subsets of proteins have been omitted. (**B**) Oxygen consumption rate (OCR) measurement performed on indicated cell lines using Seahorse XFp analyzer. Bar graphs represent indicated functional parameters calculated from the same measurements (*n* = 4). *p < 0.05, **p < 0.01, ***p < 0.001 one-way ANOVA followed by Sidak’s multiple comparisons test (**C**) Western blot analysis performed on indicated cell lines under basal conditions. (**D, E**) Western blot analysis of NDUFS1 (D) and TUFM (E) proteins in the indicated cell lines performed after 2D-Gel electrophoresis (2D-GE) under basal conditions. Arrows indicate NDFUS1 proteoforms with different molecular weights. See Fig S4B-D for quantification of western blots shown in **C-E**. (**F**) CFE assay of indicated cell lines cultured for 12 days in presence of DMSO, 0.5 µM Vemurafenib (Vem), 200 µM Phenformin (Phen) or the combination of 0.5 µM Vemurafenib + 200 µM Phenformin (Vem + Phen). Bar graphs represent the mean values of 3 independent experiments ± SEM. **p < 0.01, ***p < 0.001, one-way ANOVA followed by Dunnett’s multiple comparisons test

Gene Ontology (GO) analysis of differentially expressed protein species revealed that in both lineages, RICTOR depletion was associated with GO Cellular Components categories such as “vesicle lumen”, “ficolin rich granule lumen”, and “mitochondrial matrix” (Fig. 4A). Among upregulated moieties, we found proteins related to oxidative stress protection (SOD2, PRDX5 [M14]), mitochondrial functions (NDUFS1, PDHA1, HSD17B10, GATD3 and TUFM [M14]; ACADM, SUCLA2, OAT, ALDH1B1 and TUFM [A375]) and NAD^+^ metabolism (NAMPT, NAPRT [M14]; IDH1 [A375]). A complete list of enriched GO categories is shown in Supplementary Table S4.

Because enhanced mitochondrial respiration is typical of BRAF/MEKi-resistance [6, 24, 25, 33, 34], and in light of the mitochondrial OXPHOS gene signature identified in BRAF-mutated low RICTOR tumors (Fig. 2G, H), we found of particular interest the changes in proteins involved in metabolic pathways that fuel mitochondrial oxidative metabolism such as glutamine metabolism (OAT), mitochondrial fatty acid beta-oxidation (ADACM; HSD17B10), tricarboxylic acid cycle (SUCLA2, IDH2) and ETC (NDUFS1). Accordingly, through Seahorse analysis we found an overall increase in maximal respiration rates in all cellular backgrounds upon RICTOR knockdown, though some cell line-specific differences were identified for other parameters (Fig. 4B). Interestingly, spots identified in both M14 and A375 RICTOR-deficient cells contained TUFM, a key regulator of mitochondrial protein translation and oxidative phosphorylation [35], processes previously implicated in adaptive resistance to BRAFi in BRAF^V600E^ melanoma cells [6, 36]. The NDUFS1 protein identified in M14 cells is the core subunit of the ETC Complex I that transfers electrons from NADH to the respiratory chain, and its overexpression enhances mitochondrial functions [37]. Although we did not find significant changes in TUFM protein and NDUFS1 protein/mRNA levels between RICTOR-proficient and -deficient cells (Fig. 4C, S4A, B), the 2D-immunoblotting analysis revealed different migratory patterns of TUFM and NDUFS1 moieties in shR1 cells in both M14 and A375 cell lines (Fig. 4D, E, S4C, D). Moreover, in A375 shR1 cells, the NDUFS1 antibody detected high molecular weight protein species that were absent in shC cells (Fig. 4D).

These results indicate that the increase in TUFM and NDUFS1 expression detected by MS in RICTOR-deficient cells likely reflects the increase in proteoforms sensitive to RICTOR depletion, which may connect mTORC2 to ETC regulation. To functionally investigate the involvement of mitochondrial respiration in the BRAFi-resistant phenotype of shR1 cells, we carried out CFE survival assays in the presence of Vemurafenib and/or the ETC Complex I inhibitor Phenformin, a derivative of the anti-diabetic drug Metformin. Whereas treatment with Phenformin alone similarly reduced of ∼ 40% the clonogenic ability of both RICTOR-deficient and –proficient cells under basal conditions, when combined with Vemurafenib it selectively diminished the CFE of RICTOR-deficient cells to levels comparable to those of BRAFi-sensitive control cells (Fig. 4F). These data indicate that ETC Complex I inhibition induces a substantial rescue of the BRAFi-tolerant phenotype of shR1 cells, further indicating that mitochondrial respiration plays an important role in protecting these cells from BRAF inhibition.

### Increased NAMPT activity in RICTOR-depleted cells is crucial for their BRAFi resistance

**Fig. 5:**
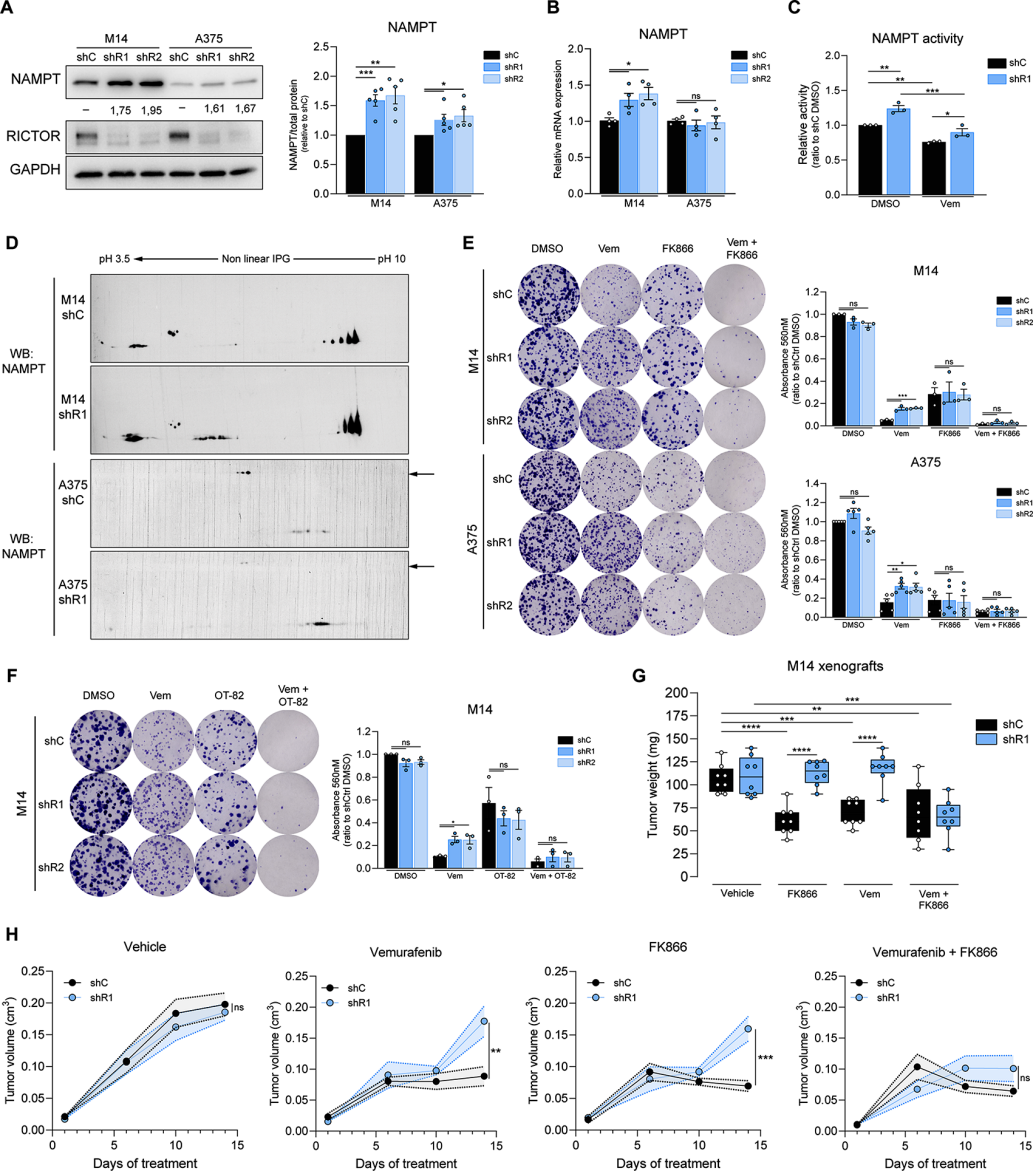
RICTOR silencing in BRAF^V600E^ melanoma cell lines leads to increased NAMPT activity which can be pharmacologically inhibited to restore sensitivity to BRAFi. (**A**) WB analysis performed on indicated cell lines under basal conditions. Densitometric quantification of NAMPT band intensity is indicated below each band. *Right panel*: densitometric quantification of WB, each value was normalized on shC cells of the same lineage. Bar graphs represent the mean values of 5 independent experiments ± SEM. *p < 0.05, **p < 0.01, ***p < 0.001, one-way ANOVA followed by Dunnett’s multiple comparisons test. (**B**) qRT-PCR analysis of NAMPT gene expression in the indicated cell lines under basal conditions. Bar graphs represent mean values of 4 independent experiments ± SEM. *p < 0.05, one-way ANOVA followed by Dunnett’s multiple comparisons test. (**C**) NAMPT enzymatic activity of M14 cells treated for 24 h with 5 µM Vemurafenib (Vem) or vehicle control (DMSO), normalized on untreated shC cells. Bar graphs represent mean values of 3 independent experiments ± SEM. *p < 0.05, **p < 0.01, ***p < 0.001, two-way ANOVA followed by Tukey’s multiple comparisons test. (**D**) WB analysis of NAMPT protein in the indicated cell lines performed after 2D-GE under basal conditions. Arrows indicate NDFUS1 proteoforms with different molecular weights. See Fig S5B for quantification. (**E**) CFE assay of indicated cell lines cultured for 12 days in presence of DMSO, 0.5 µM Vem, 2.5 nM FK866 or the combination of 0.5 µM Vem + 2.5 nM FK866 (Vem + FK866). Bar graphs represent the mean values of independent experiments ± SEM (*n* = 3 for M14 cells; *n* = 4 for A375 cells). *p < 0.05, **p < 0.01, ***p < 0.001, ****p < 0.0001, one-way ANOVA followed by Dunnett’s multiple comparisons test. (**F**) CFE assay of M14 cells cultured for 12 days in presence of DMSO, 0.5 µM Vem, 1.2 nM OT-82 or the combination of 0.5 µM Vem + 1.2 nM OT-82 (Vem + OT-82). Bar graphs represent the mean values of 3 independent experiments ± SEM. *p < 0.05, one-way ANOVA followed by Dunnett’s multiple comparisons test. (**G**) Tumor weights of tumor xenografts of shC or shR1 M14 cells in NSG mice (*n* = 8 mice/group), treated for 14 days with the indicated drugs. **p < 0.01, ***p < 0.001, ****p < 0.0001, two-way ANOVA followed by Tukey’s multiple comparisons test. (**H**) Growth curves of M14 shC or shR1 xenografts used for tumor weight measurement, each graph refers to the treatment indicated at the top. *p < 0.05, **p < 0.01, two-way ANOVA followed by Tukey’s multiple comparisons test performed at the experimental endpoint

Our proteomic analysis also identified in RICTOR-depleted cells an increase in the expression of proteins involved in NAD^+^ metabolism and biosynthesis (NAMPT, NAPRT, IDH1). Specifically, NAMPT is the rate-limiting enzyme of the NAD^+^ salvage pathway in mammals and increases in its expression/activity play key roles in melanoma therapeutic resistance to BRAF/MEKi [7, 23]. Albeit M14 and A375 cell lines differ in their absolute levels of NAMPT protein expression, with the former having > 5 fold higher protein amount than the latter under basal conditions, in both lineages, RICTOR knockdown induced a significant increase in NAMPT levels relative to control conditions (Fig. 5A). RICTOR-depleted M14 cells also displayed increased NAMPT mRNA levels, while RICTOR-proficient and –deficient A375 cells possessed similar level of *NAMPT* transcript (Fig. 5B). Measurements of NAMPT catalytic activity revealed an increase in M14 shR1 cells, both under basal conditions and upon Vemurafenib treatment (Fig. 5C). In A375 shC cells NAMPT activity was found below the threshold of detection of our enzymatic assay (< 0.10 pmol/h/µg), which is likely due to their intrinsically lower NAMPT levels compared to the M14 cell lineage (Fig. 5A). Nevertheless, in shR1 A375 cells, NAMPT activity was measurable both under basal conditions and upon Vemurafenib treatment (0.183 pmol/h/µg and 0.123 pmol/h/µg, respectively), indicating that also in this cell lineage the catalytic activity of the enzyme is increased by RICTOR depletion. Total NAD^+^ content in M14 cells was not affected by RICTOR silencing or Vemurafenib treatment (Fig S5A), although we cannot rule out the occurrence of a localized accumulation of NAD^+^ in specific cellular compartments or a faster NAD^+^ consumption in RICTOR-silenced cells by NAD^+^-consuming enzymes.

Immunoblotting analysis aimed to detect differences in NAMPT moieties after 2D electrophoresis between RICTOR-deficient and -proficient cells also revealed changes not only in levels but also in the migratory pattern of some NAMPT proteoforms in both M14 and A375 backgrounds (Fig. 5D, S5B). Overall, our analysis revealed that RICTOR deficiency is coupled to an increased NAMPT protein expression and/or activity in BRAF^V600E^ melanoma cells, and that both transcriptional and post-transcriptional mechanisms can account for this effect in a cell lineage-specific manner.

To verify whether in shR1 cells the overall increase in NAMPT activity plays a role in their BRAFi resistance, we compared the survival of mTORC2-deficient and –proficient cells by CFE assays carried out in the presence of Vemurafenib and/or two structurally unrelated NAMPT inhibitors (NAMPTi), FK866 and OT-82 (Fig. 5E, F). Similar to Phenformin treatment, exposure of cells to low nanomolar concentrations of both FK866 and OT-82 caused a comparable reduction of CFE in both genotypes. In the A375 cellular background, FK866 alone caused a more pronounced drop in cell clonogenicity in all genotypes as compared to the M14 lineage. In the latter, the combination of Vemurafenib + FK866 or OT-82 caused a drop in the clonogenicity of RICTOR–deficient cells equivalent to that observed in control cells. Thus, the inhibition of NAMPT enhances the response of RICTOR-deficient cells to Vemurafenib, indicating that the increased activity of the enzyme in these cells is critical for their BRAFi resistance.

To verify if the NAMPT-dependent BRAFi-resistant phenotype induced by RICTOR depletion holds true also in vivo, we injected M14 RICTOR-deficient and -proficient cells subcutaneously in NOD scid gamma (NSG) mice, and when tumors became palpable, mice were treated with Vemurafenib and/or FK866. Tumor growth was progressively monitored, and after 14 days of treatment the animals were sacrificed and tumors were measured and weighted. As shown in Fig. 5G, H and S5C, whereas tumors generated by RICTOR-proficient cells displayed a decrease in weight of about 50% in response to Vemurafenib, weight and the volume of RICTOR-deficient tumors at the experimental endpoint were comparable to those of vehicle-treated control tumors. The combination of Vemurafenib and FK866 induced instead in RICTOR-deficient tumors a growth inhibition similar to that of Vemurafenib-treated control tumors. The sensitivity of RICTOR-deficient and -proficient xenografts to the treatment with NAMPTi alone differed between the two genotypes, as shR1 tumors resulted overall less responsive to the individual FK866 treatment. Nevertheless, even in this in vivo setting, NAMPT inhibition combined to Vemurafenib treatment significantly enhanced the responses of RICTOR-deficient xenografts to Vemurafenib, confirming that NAMPT activity is key for counteracting the response of these tumors to BRAFi.

## Discussion

Our research uncovers an unforeseen role for RICTOR/mTORC2 downregulation in promoting the development of resistance to BRAFi-based therapies in BRAF^V600E^ melanoma cells. This discovery seems at odds with the well-established role of mTORC2 signaling in promoting cell growth and survival across various pathophysiological contexts [14, 38–40], including melanoma progression [38, 39]. However, our findings align with emerging evidence indicating tumor suppressive functions for mTORC2 in different settings [15, 16, 41].

In fact, we demonstrate that downregulation of RICTOR in drug-naïve cells accelerates the acquisition of resistance to Vemurafenib, indicating a role for mTORC2 downregulation during the early adaptation to BRAFi. Indeed, RICTOR-depleted melanoma cells exhibit intrinsic tolerance to BRAFi, either alone or in combination with MEKi. This adaptive role is further supported by the observation of endogenous RICTOR decline during the initial phase of response of drug-naïve cells to BRAF inhibition. Of note, we described this decrease also in cellular colonies surviving sustained Vemurafenib treatment, which likely represent the precursors of future BRAFi-resistant cell populations.

Our data suggest that a decrease in MAPK signaling is the primary trigger for this phenomenon, which occurs through processes leading to the reduction of RICTOR protein levels. Although the specific molecular mechanism remains unidentified, we propose that BRAFi-induced RICTOR downregulation relies on the modulation of its protein stability, as it is sensitive to proteasomal inhibition and unrelated to changes in mRNA levels. These data are consistent with recent findings that highlight the importance of post-translational mechanisms on the overall regulation of RICTOR/mTORC2 in different models [42–45]. However, it remains possible that other unidentified factors (like microRNAs or changes in mRNA translation [8, 46, 47]) may also contribute to regulate RICTOR protein levels at different stages of melanoma progression.

In many cases, mTORC2 functions have been approached as a prospective target in tumor cells displaying advanced stages of therapeutic resistance. These cells are also typically characterized by upregulation in growth factor receptor signaling [3], for which mTORC2 acts as amplifier of mitogenic stimuli [9]. Consistently, we also found a generalized decrease in basal clonogenicity after inducing RICTOR depletion in irreversibly resistant cells. However, our study shows that the specific response of RICTOR-deficient BiR cells to BRAF/MEKi in this context ranges from an increased sensitivity (in A375 BiR cells) to a stimulation of clonogenicity to BRAF/MEKi (in M14 and SK-MEL-28 cells). The described behaviour of BiR A375 cells is in agreement with the finding of Jebali et al. [38], that showed enhanced responses of BRAFi-resistant A375 cells to mTOR(C2) inhibition.

Thus, although the effects of RICTOR depletion in the responses of cells to targeted therapy may vary in BiR cells, RICTOR knockdown in drug-naïve cells consistently promotes a faster progression towards BRAFi resistance.

Proteomic analysis of RICTOR-deficient cells revealed significant alterations in proteins related to NAD^+^ biosynthesis and mitochondrial processes. We observed that RICTOR knockdown correlates with increased NAMPT expression/activity, with multi-layered and cell lineage-specific mechanisms leading to NAMPT upregulation. RICTOR deficiency alters post-translational modifications in NAMPT likely affecting its activation and/or stability, which is suggestive of a complex interplay between mTORC2 signaling and NAD^+^ biosynthesis in melanoma. 2D proteomic analysis also identified a differential representation of NDUFS1 (core component of ETC Complex I) and TUFM (mitochondrial translation regulator) proteoforms, suggesting mTORC2 regulation on mitochondrial dynamics in melanoma. This is further supported by GSEA analysis of melanoma TCGA data, which indicates upregulation of mitochondrial gene signatures in low RICTOR tumors. The connection between increase in mitochondrial functions and resistance to BRAFi is supported by our experimental evidence, as both NAMPT and ETC inhibition reverses the BRAFi tolerance of RICTOR-depleted cells. Our findings are supported by previous works that show NDUFS1 modulation by post-translational modifications, and by poorly-characterized associations between RICTOR downregulation and gain of mitochondrial functions [18, 19, 48].

The clinical significance of our findings is supported by analysis of the cutaneous melanoma subset in the TCGA database, which reveals a positive correlation between lower RICTOR protein and poorer outcomes in patients with BRAF^V600E^ mutations. Conversely, higher levels of RAPTOR mRNA correlate with worse prognosis, consistent with the pro-tumorigenic role of mTORC1 in melanoma [13]. These results suggest that the use of pan-mTOR inhibitors, often proposed in the context of melanoma treatment [40, 49], could potentially represent a double-edged sword, with mTORC1 inhibition potentially counteracting therapeutic resistance while mTORC2 inhibition exacerbating it.

This highlights the importance of assessing RICTOR protein levels for diagnostic and prognostic purposes, given the poor correlation between RICTOR mRNA and protein levels in melanoma patients. Accordingly, patients with low RICTOR tumors may benefit from NAMPTi therapy in combination with targeted therapy, with low RICTOR levels serving as a predictive biomarker for NAMPTi response, thus improving the applications of NAMPT inhibitors in the clinics.

An outstanding question is whether Immune Checkpoint Inhibitors would represent a valuable alternative for the treatment of BRAF-mutated low RICTOR tumors. This is especially relevant in light of recent evidence that connect tumor immunogenicity with metabolic alterations reminiscent of those we observed in RICTOR-deficient cells [50, 51].

In conclusion, our study reveals an important role for RICTOR/mTORC2 in modulating response to BRAFi therapy, highlighting potential therapeutic avenues. In particular, RICTOR protein may serve as a suitable biomarker for guiding therapeutic strategies, rather than as an actionable target per se, as often proposed. These findings strongly emphasize the need for personalized treatment strategies based on tumor molecular profiles.

### Electronic supplementary material

Below is the link to the electronic supplementary material.

Supplementary Material 1

Supplementary Material 2

Supplementary Material 3

Supplementary Material 4

Supplementary Material 5

Supplementary Material 6

Supplementary Material 7

Supplementary Material 8

Supplementary Material 9

Supplementary Material 10

Supplementary Material 11

Supplementary Material 12

Supplementary Material 13

Supplementary Material 14

Supplementary Material 15

Supplementary Material 16

## Data Availability

Full and uncropped western blots were uploaded as Supplementary Material. The datasets generated during and/or analyzed during the current study are available from the corresponding author on reasonable request. The mass spectrometry proteomics data have been deposited to the ProteomeXchange Consortium via the PRIDE partner repository (https://www.ebi.ac.uk/pride/) with the dataset identifiers PXD045346 and PXD050614.

## Abbreviations

AKT: V-Akt Murine Thymoma Viral Oncogene Homolog
ACADM: acyl-CoA dehydrogenase medium chain
ALDH1B1: Aldehyde dehydrogenase 1 family member B1
AXL: AXL receptor tyrosine kinase
BRAF: v-Raf murine sarcoma viral oncogene homolog B
DEPTOR: DEP domain-containing mTOR-interacting protein
ERK1/2: Extracellular signal-regulated kinase1/2
ETC: Electron Transport Chain
GAPDH: Glyceraldehyde 3-phosphate dehydrogenase
GATD3: Glutamine Amidotransferase Class 1 Domain Containing 3
GSEA: Gene Set Enrichment Analysis
HSD17B10: Hydroxysteroid 17-beta dehydrogenase 10
IDH1: Socitrate dehydrogenase (NADP(+)) 1
MAPK: Mitogen-activated protein kinase
MEK1/2: Mitogen-activated protein kinase kinase1/2
MEM: Minimum Essential Media
MITF: Melanocyte inducing transcription factor
mLST8: Mammalian lethal with Sect. 13 protein 8
mTOR: mammalian target of rapamycin
NAD: Nicotinamide adenine dinucleotide
NAMPT: Nicotinamide phosphoribosyltransferase
NAPRT: Nicotinate phosphoribosyltransferase
NDRG1: N-myc downstream regulated gene 1
NDUFS1: NADH: Ubiquinone Oxidoreductase Core Subunit S1
NSG: NOD/SCID/IL2γr^null^
OAT: ornithine aminotransferase
OXPHOS: oxidative phosphorylation
PDHA1: pyruvate dehydrogenase E1 subunit alpha 1
PI3K: Phosphoinositide 3-kinase
PKCα: Protein kinase C-α
PRAS40: proline-rich Akt substrate of 40 kDa
PRDX5: Peroxiredoxin 5
PROTOR1/2: Protein observed with Rictor-1/2
P90RSK: Ribosomal protein S6 kinase A1
RB: RB transcriptional corepressor 1
RICTOR: Rapamycin-insensitive companion of mammalian target of rapamycin
SGK1: Serum and glucocorticoid-regulated kinase 1
SIN1: Stress-activated protein kinase-interacting protein
SOD2: Superoxide dismutase
SOX10: SRY-box transcription factor 10
SUCLA2: Succinate-CoA ligase ADP-forming subunit beta
TCGA: The cancer genome atlas
TUFM: Tu translation elongation factor, mitochondrial
