# Supplementary figures and images for: RICTOR/mTORC2 downregulation in BRAFV600E melanoma cells promotes resistance to BRAF/MEK inhibition
Source: Mol Cancer. 2024 May 16;23:105. doi: 10.1186/s12943-024-02010-1 (PMC11097536; doi:10.1186/s12943-024-02010-1)

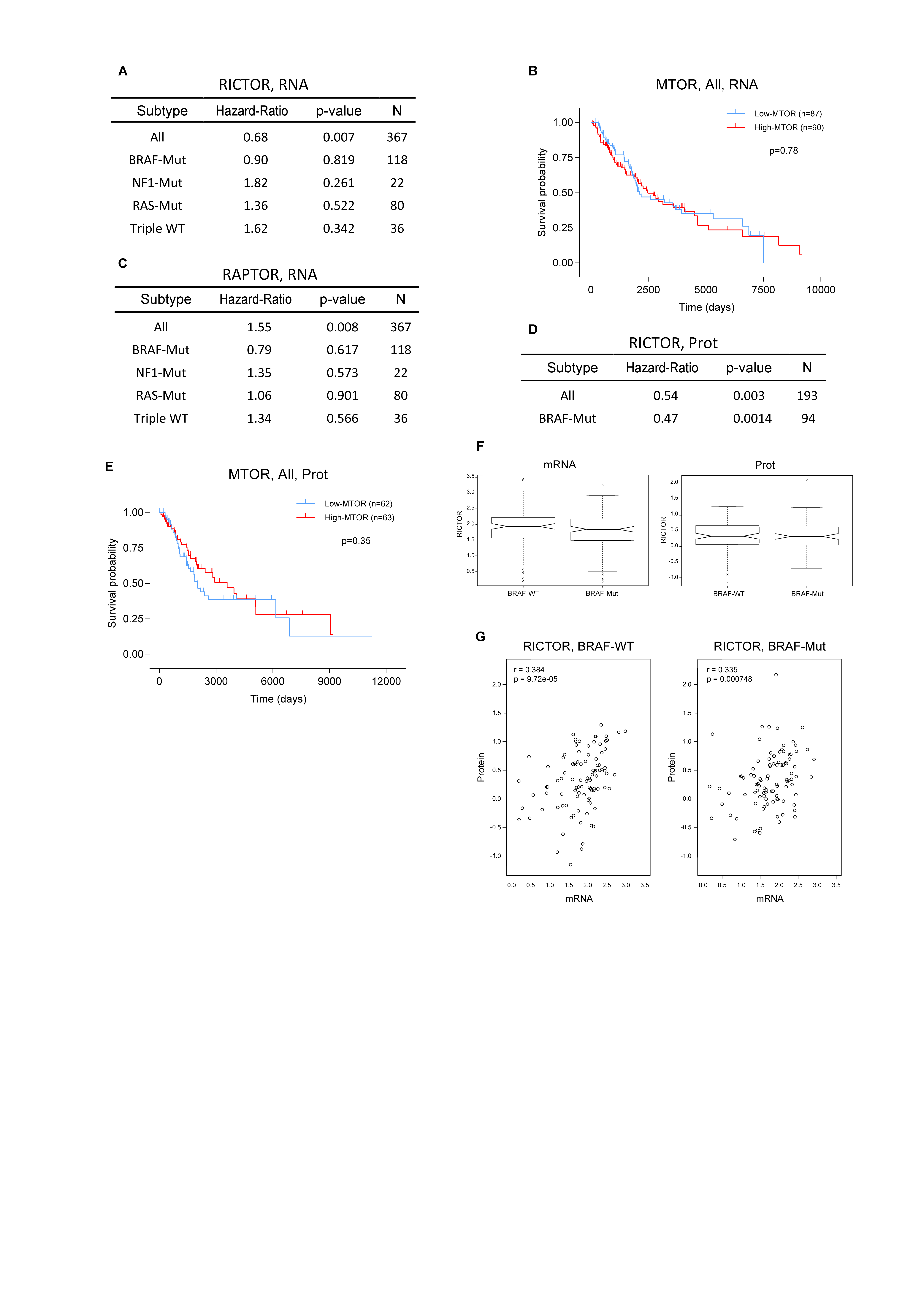

Supplement: Supplementary file 1 — Supplementary Material 1 [file 12943_2024_2010_MOESM1_ESM.tif]

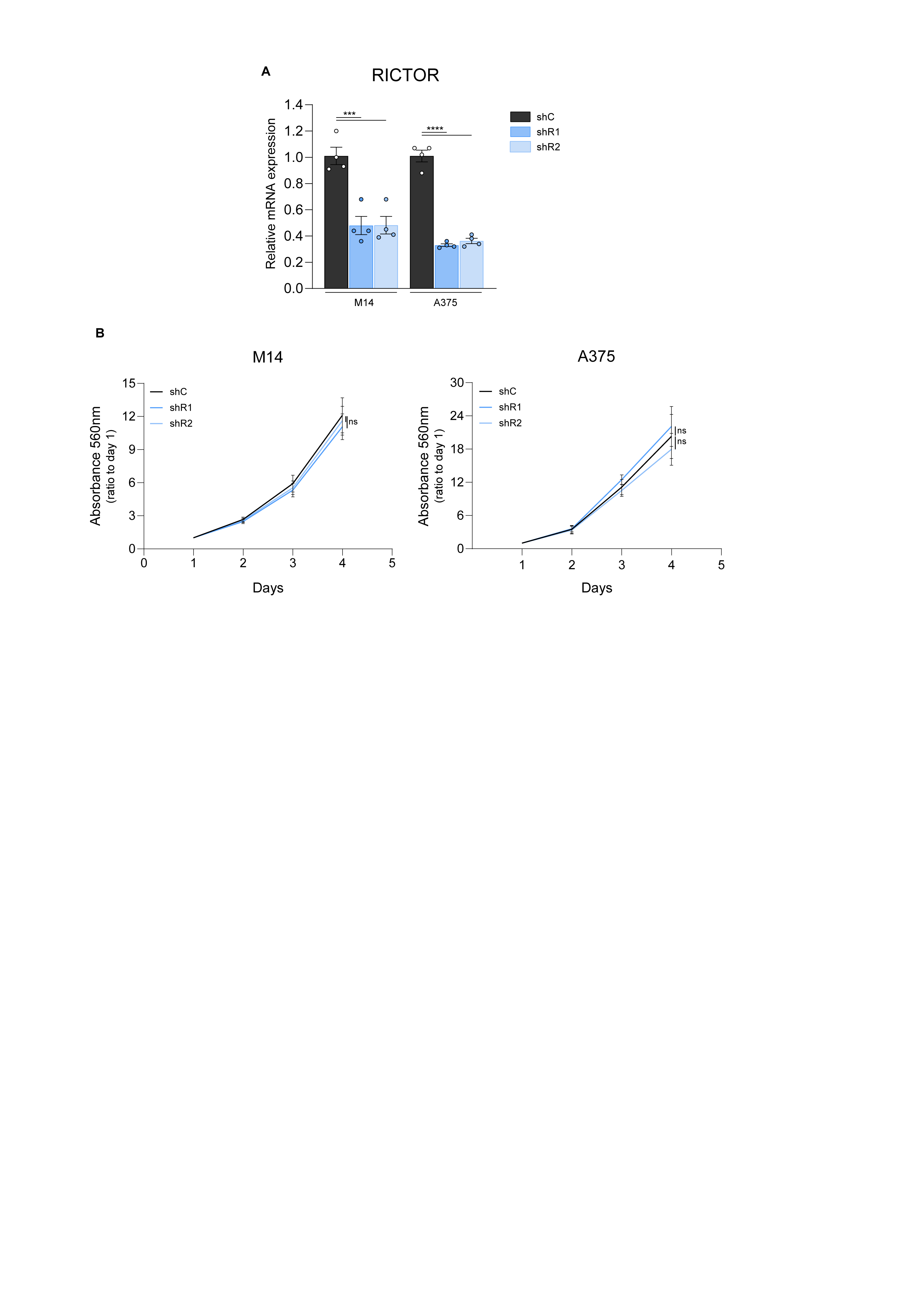

Supplement: Supplementary file 2 — Supplementary Material 2 [file 12943_2024_2010_MOESM2_ESM.tif]

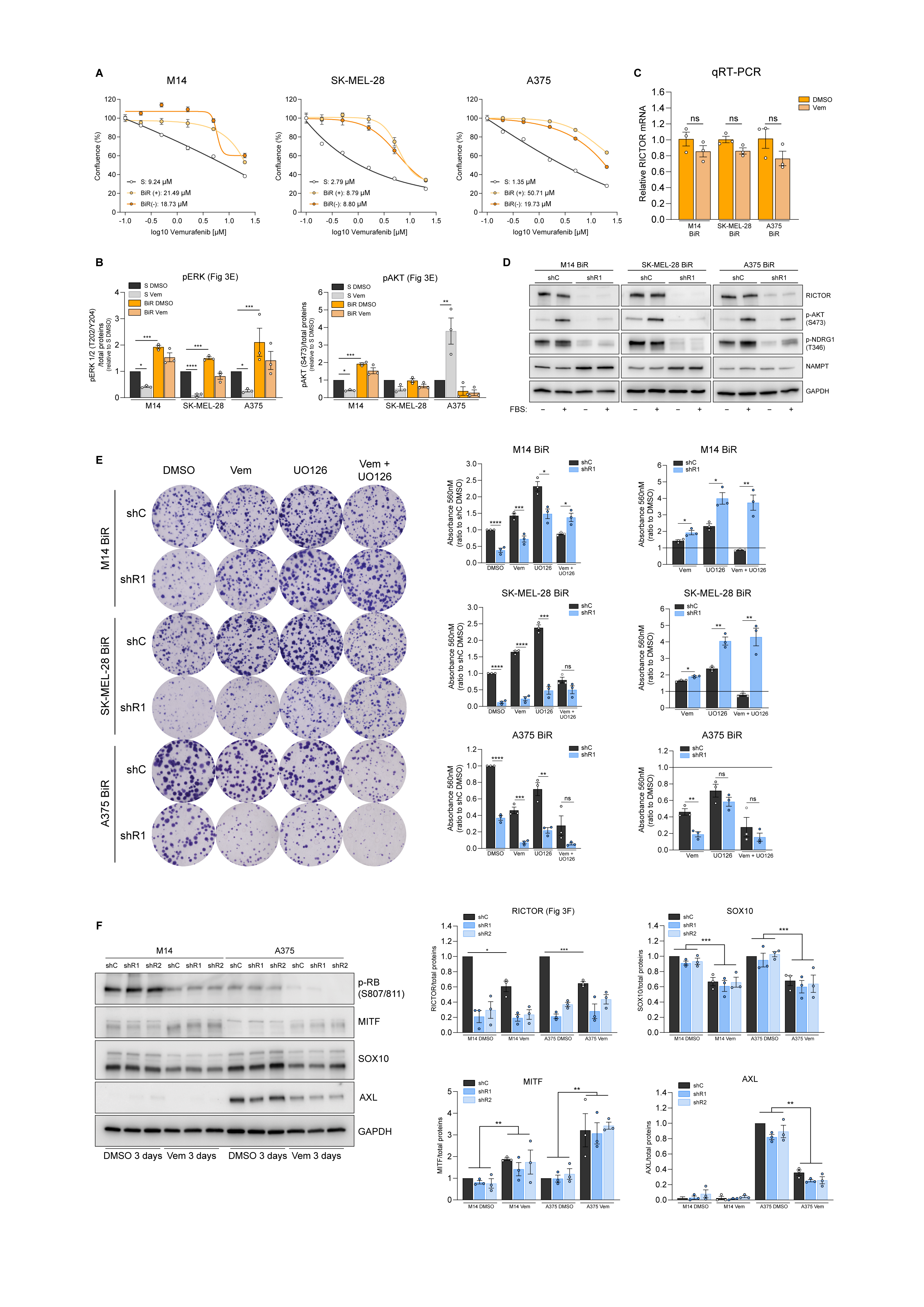

Supplement: Supplementary file 3 — Supplementary Material 3 [file 12943_2024_2010_MOESM3_ESM.tif]

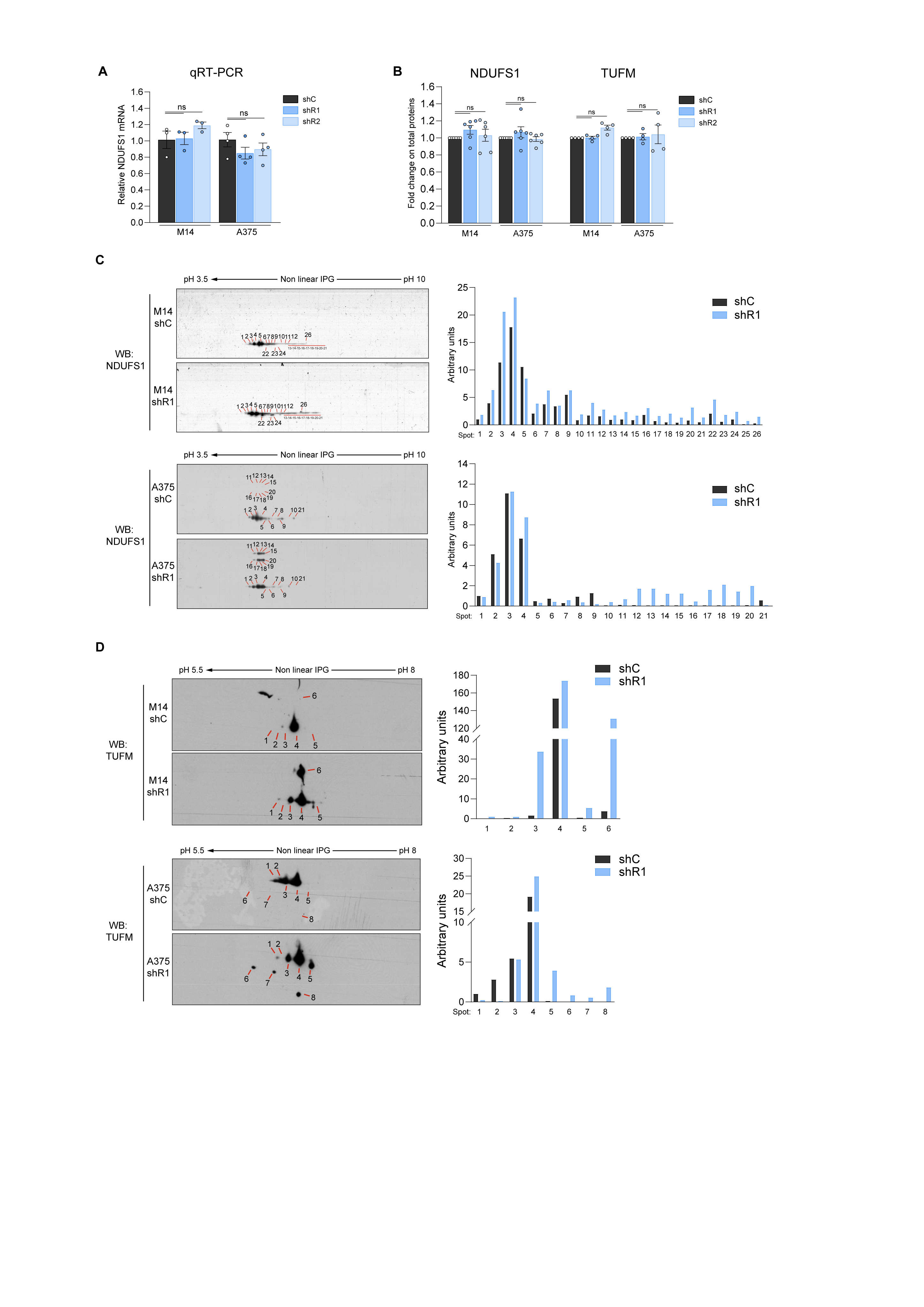

Supplement: Supplementary file 4 — Supplementary Material 4 [file 12943_2024_2010_MOESM4_ESM.tif]

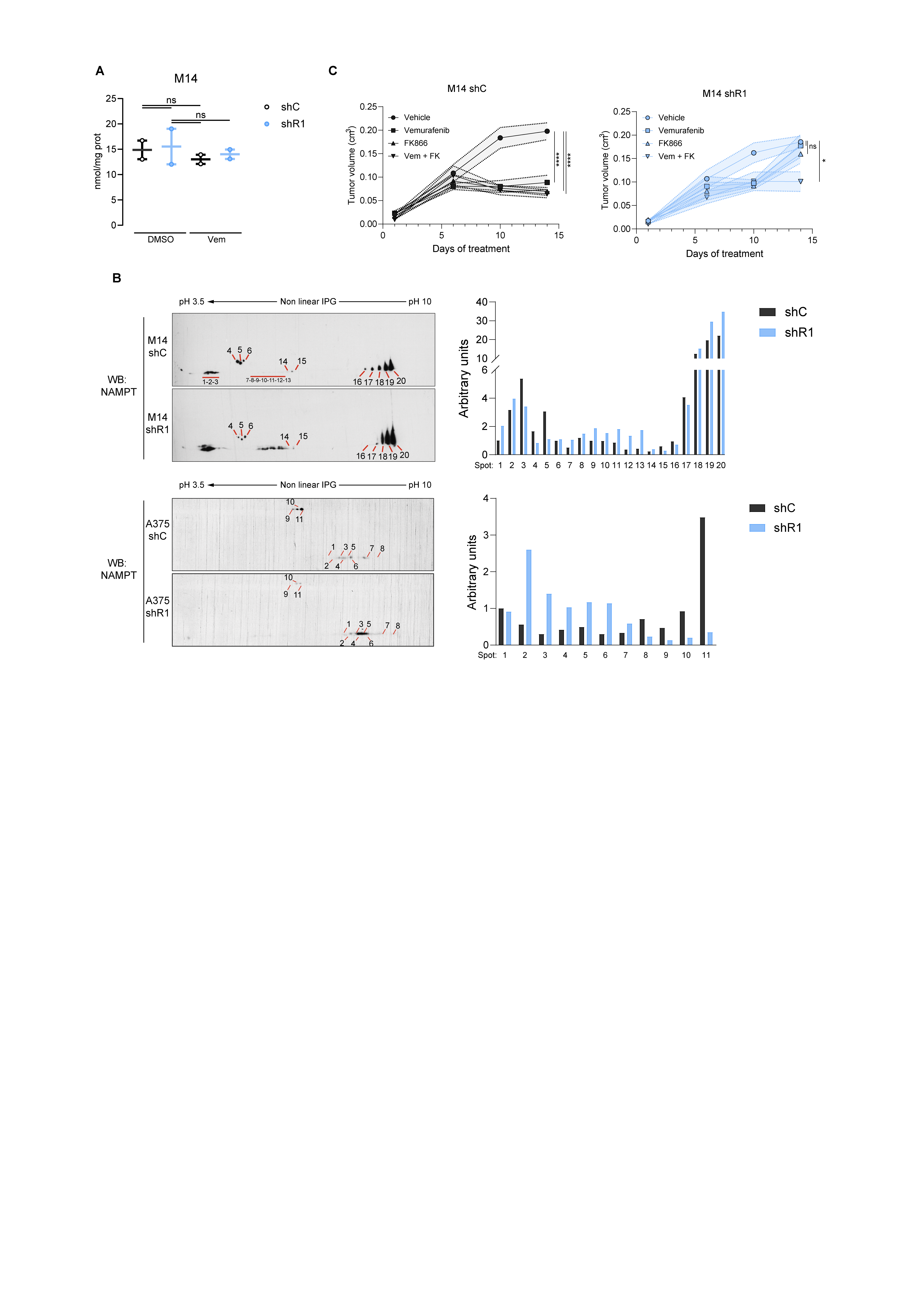

Supplement: Supplementary file 5 — Supplementary Material 5 [file 12943_2024_2010_MOESM5_ESM.tif]

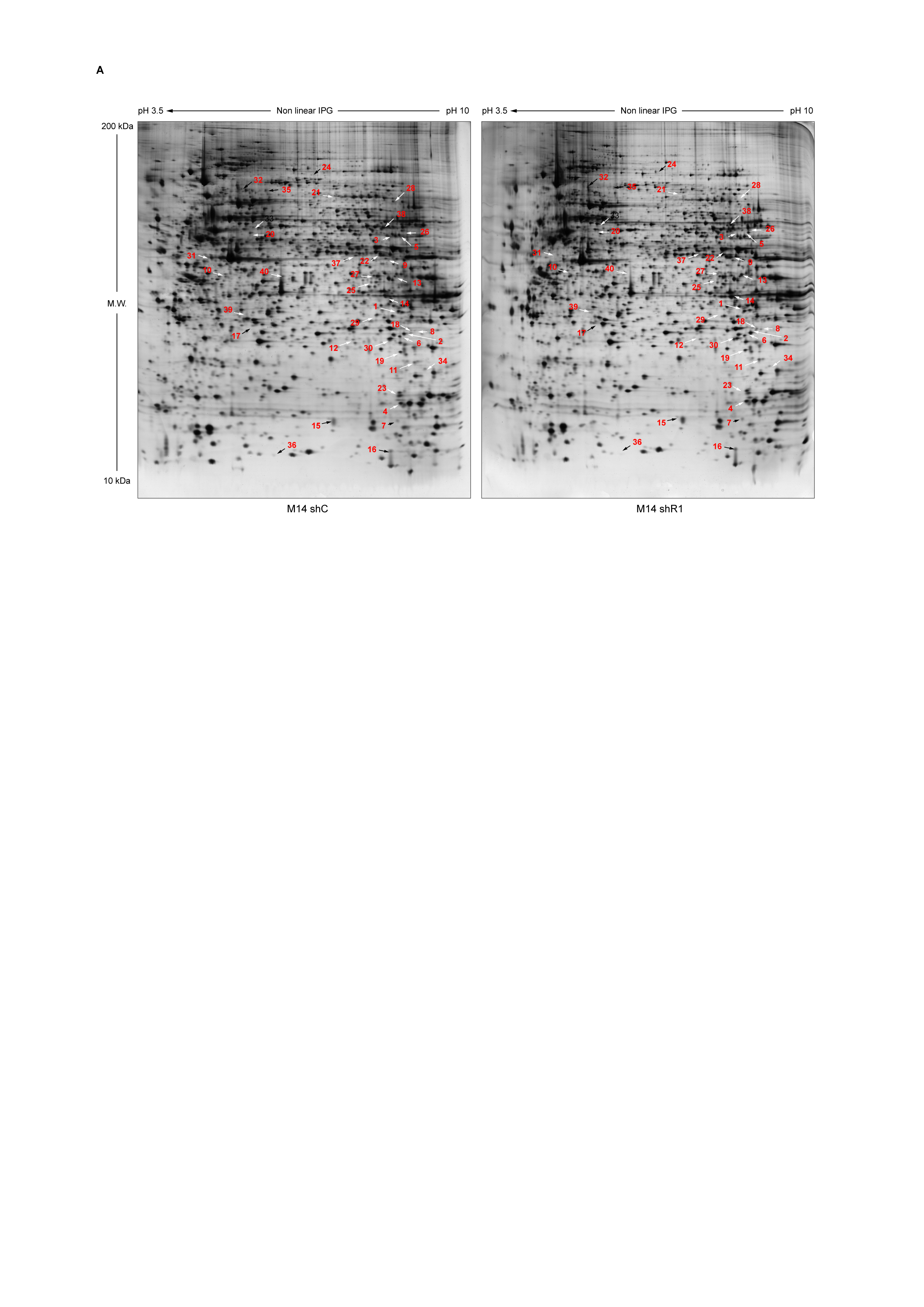

Supplement: Supplementary file 6 — Supplementary Material 6 [file 12943_2024_2010_MOESM6_ESM.tif]

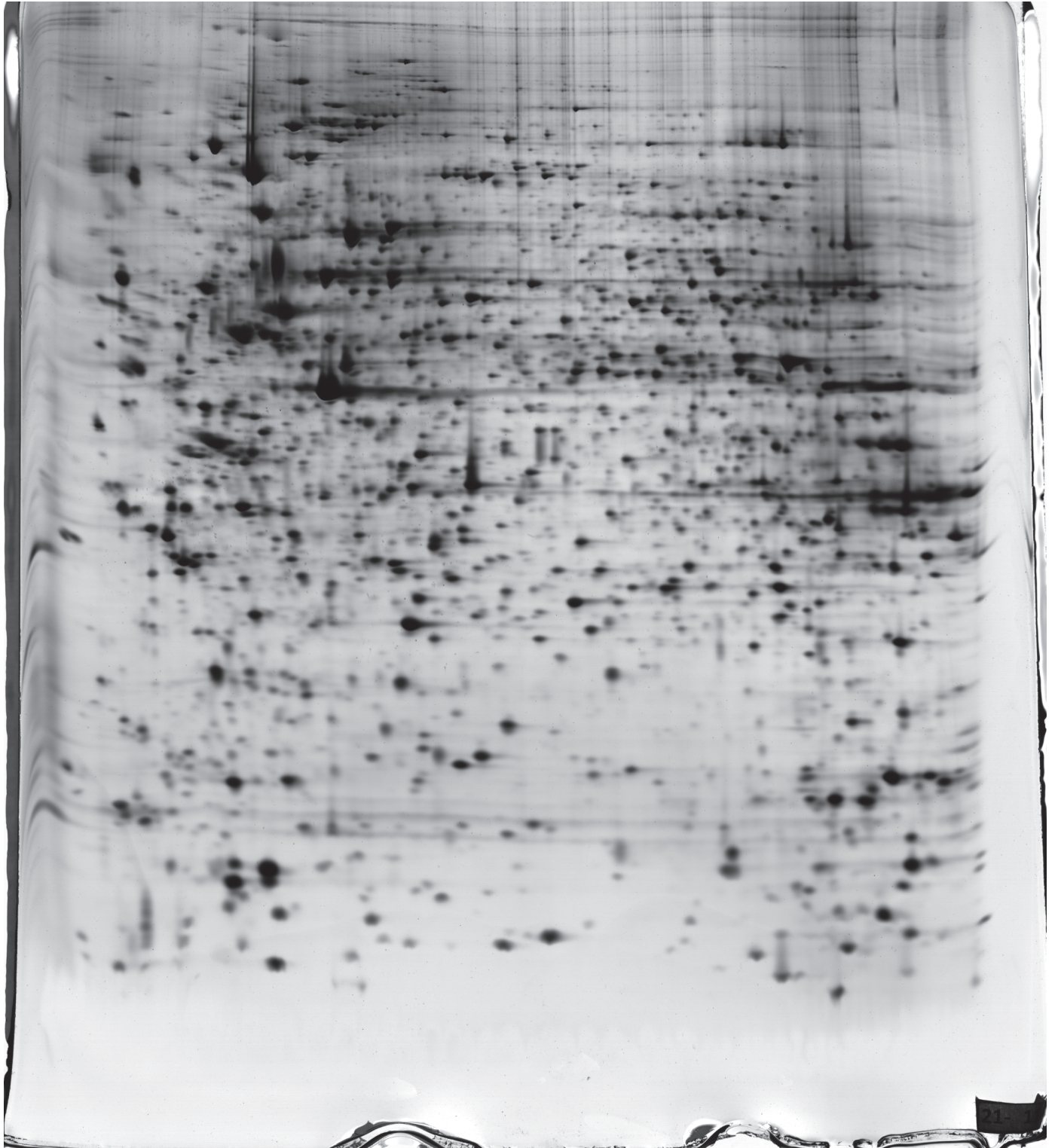

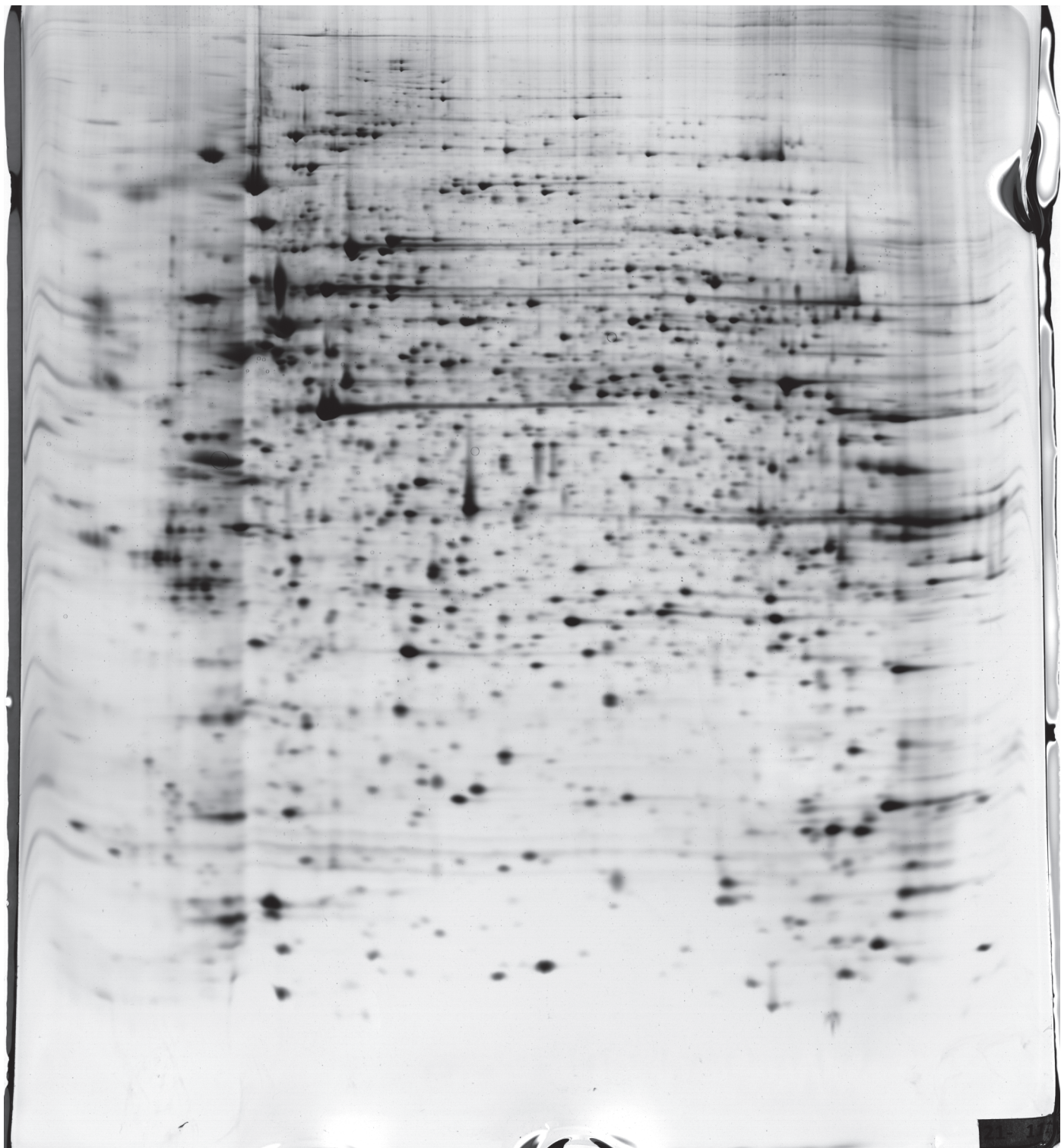

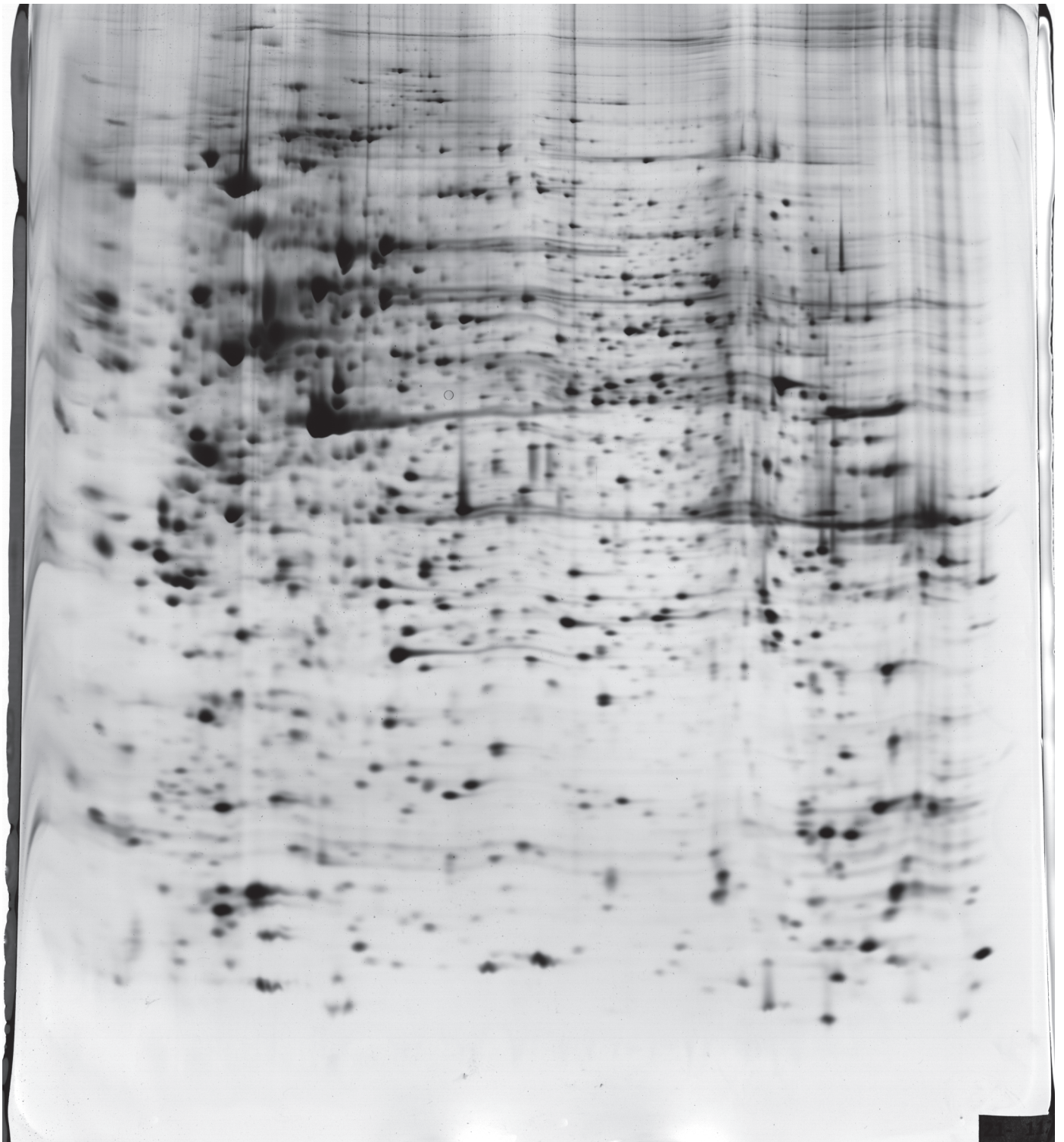

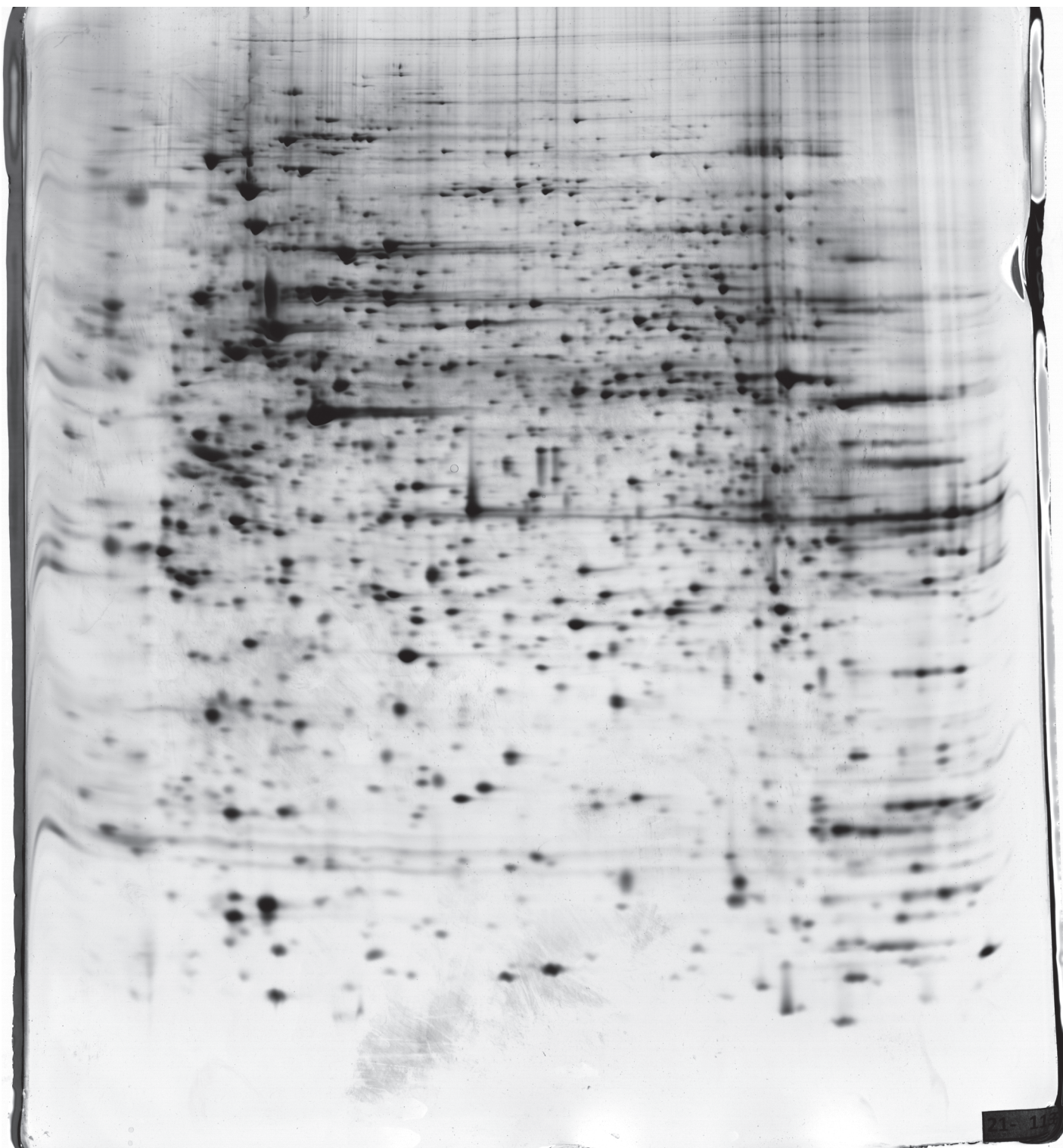

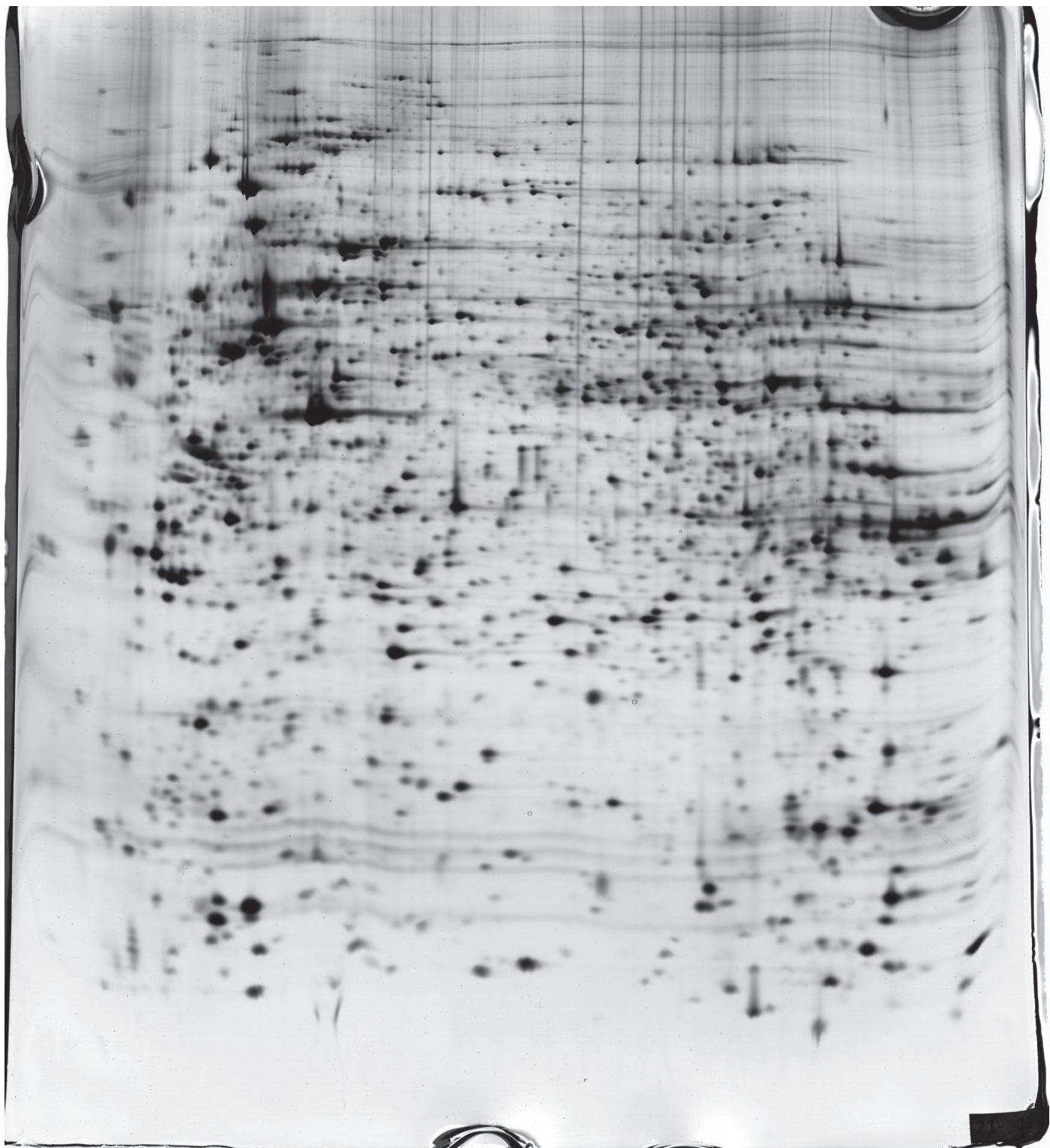

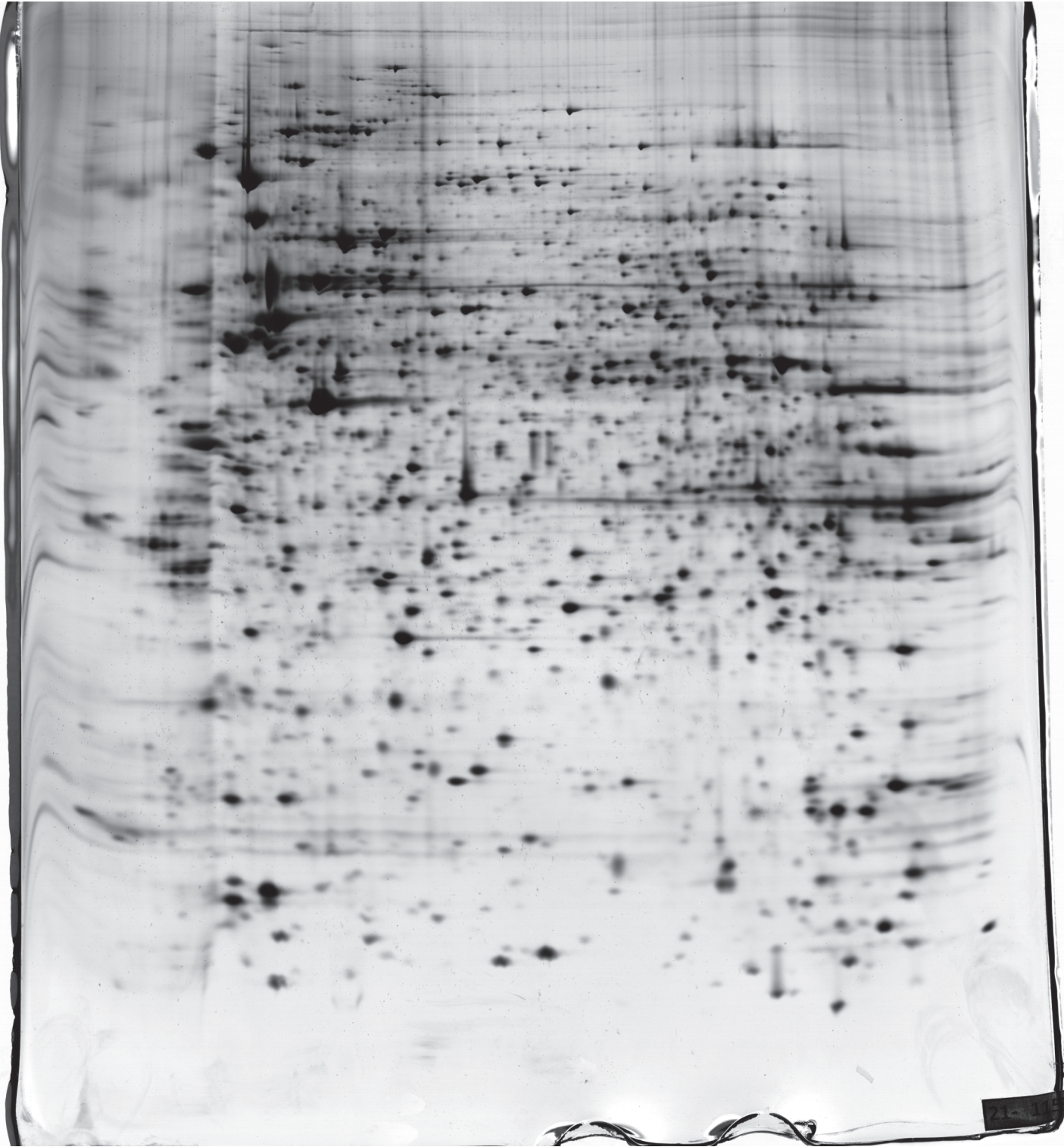

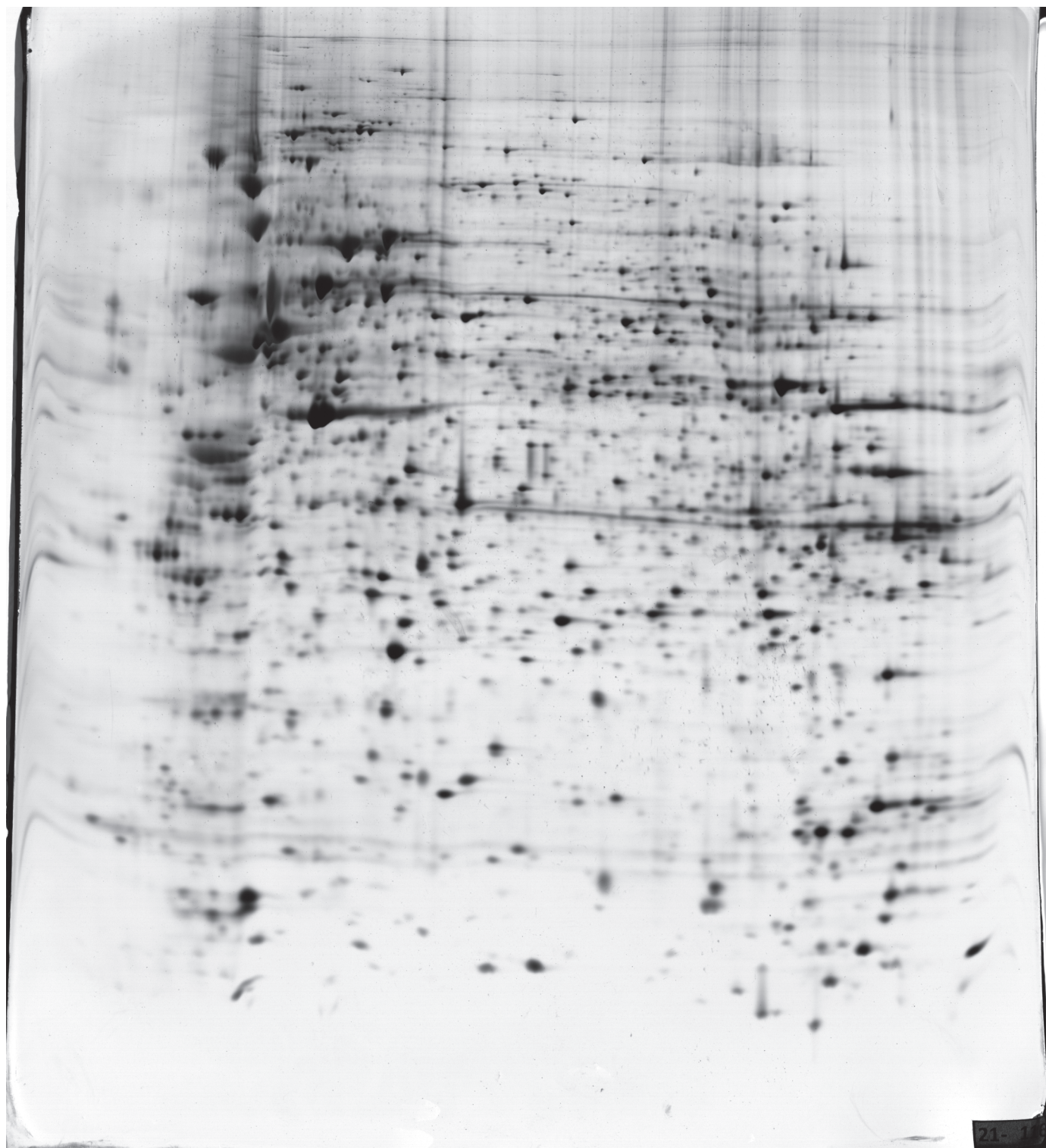

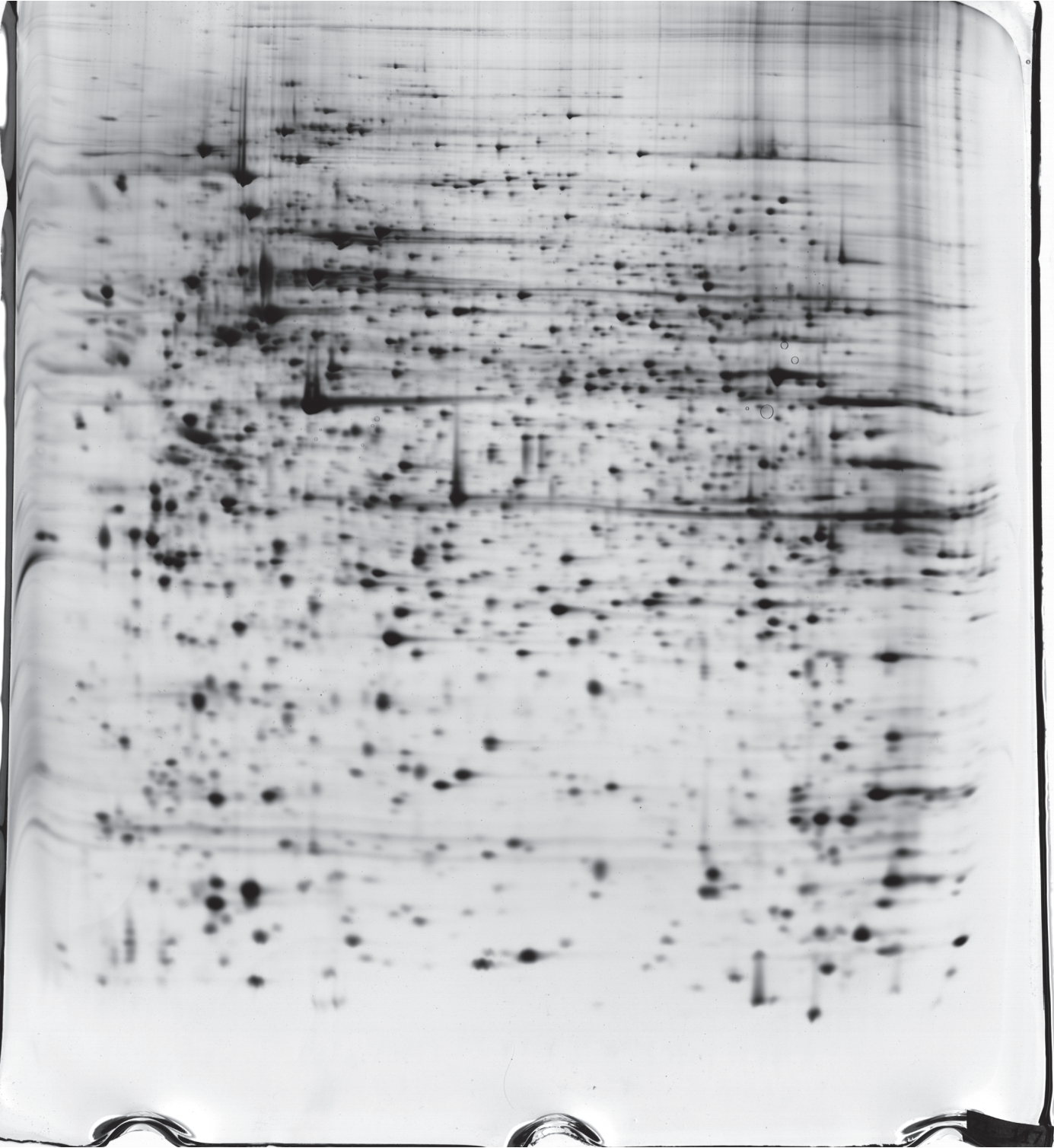

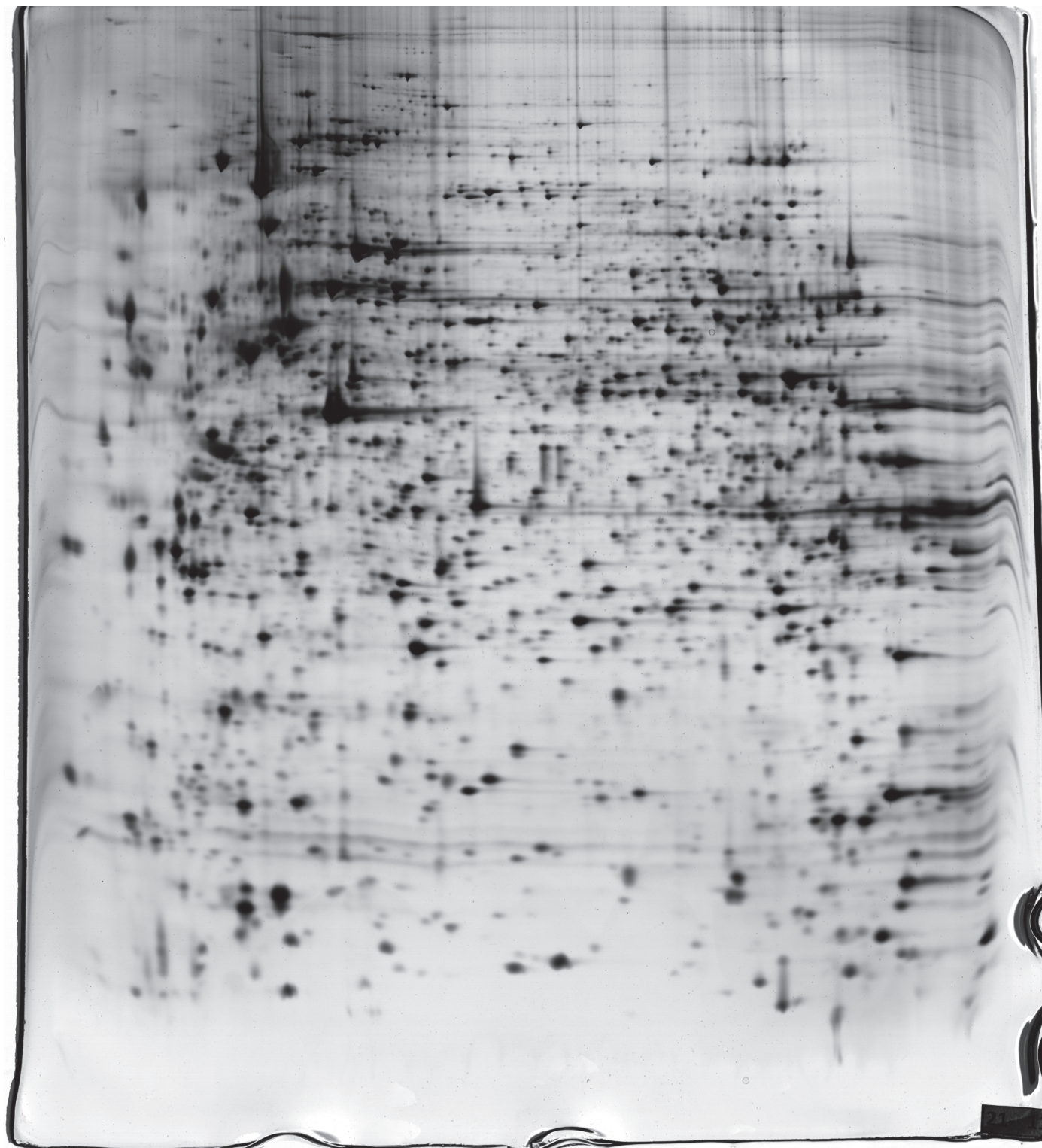

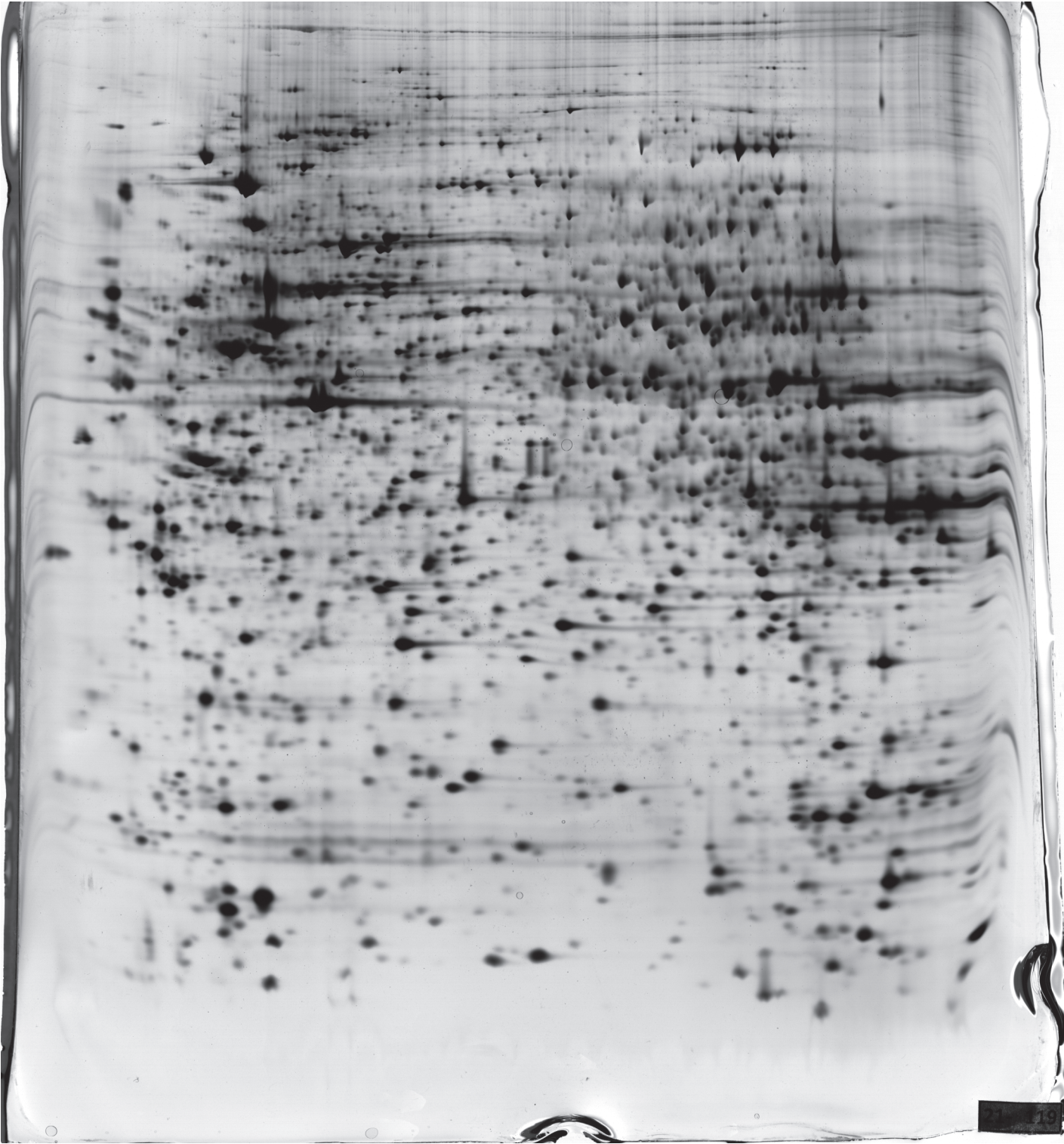

Supplement: Supplementary file 14 — Supplementary Material 14 [file 12943_2024_2010_MOESM14_ESM.pdf]
