# Supplementary material for: RICTOR/mTORC2 downregulation in BRAFV600E melanoma cells promotes resistance to BRAF/MEK inhibition
Source: Mol Cancer. 2024 May 16;23:105. doi: 10.1186/s12943-024-02010-1 (PMC11097536; doi:10.1186/s12943-024-02010-1)
Supplement: Supplementary file 11 — Supplementary Material 11 [file 12943_2024_2010_MOESM11_ESM.docx]

**SUPPLEMENTARY FIGURE LEGENDS**

**Supplementary Figure S1. Analysis of Melanoma patients’ data from the TCGA database.**

(**A, C**) Cox regression analysis of the whole SKCM dataset (All) or after stratifying patients based on genetic subtypes (BRAF-Mut, NF1-Mut, RAS-Mut, Triple WT), using RICTOR RNA (**A**) or RAPTOR RNA (**C**) levels as continuous independent variables. “All” category also includes tumors for which information about the genetic subtype is not available and thus cannot be assigned to specific genetic subtypes. Number of patients (N), hazard-ratio and p-values calculated by likelihood ratio test are indicated for each subtype. (**B, E**) Kaplan-Meier survival analysis of the entire SKCM dataset without considering BRAF mutational status (All), obtained from gene expression database (RNA) (**B**) or from RPPA (Prot) (**E**) data. High-MTOR = fourth quartile; Low-MTOR = first quartile. Number of patients for each group and p-value calculated by Log-rank test are indicated in individual graphs. (**D**) Cox regression analysis of the whole (All) SKCM RPPA dataset or after filtering for annotated BRAF Hotspot Mutations (BRAF-Mut) using RICTOR protein level as continuous independent variable. Number of patients (N), hazard-ratio and p-values calculated by likelihood ratio test are indicated for All and BRAF-Mut groups. (**F**) Expression levels of RICTOR mRNA (*left panel:* BRAF-WT n=138, BRAF-Mut n=118) and protein (*right panel:* BRAF-WT n=99, BRAF-Mut n=94) in BRAF-WT and BRAF-Mut patients of the SKCM dataset. (**G**) Correlation analysis of RICTOR mRNA and protein levels in BRAF-WT (*left panel*) and BRAF-Mut (*right panel*) patients of the SKCM dataset. Pearson correlation coefficient (r) and p-value are indicated for each group (BRAF-WT n=99; BRAF-Mut n=94).

**Supplementary Figure S2. RICTOR downregulation in M14 and A375 cell lines does not affect basal proliferation rates.**

(**A**) qRT-PCR analysis of RICTOR gene in M14 and A375 cell lines transduced with scramble shRNA (shC) or with two RICTOR-targeting shRNAs (shR1 and shR2). Bar graphs represent the mean values of 4 independent experiments ± SEM. ***p < 0.001, *****p* < 0.0001, one-way ANOVA followed by Dunnett’s multiple comparisons test. (**B**) Proliferation assay performed on M14 and A375 cell lines transduced with scramble shRNA (shC) or with two RICTOR-targeting shRNAs (shR1 and shR2). Values normalized on corresponding absorbance data at day 1. Curves represent the mean values of independent experiments (M14 n=6; A375 n=3) ± SEM. ns = not significant, one-way ANOVA followed by Dunnett’s multiple comparisons test.

**Supplementary Figure S3. Analysis of drug response and effects of RICTOR knockdown in cells irreversibly resistant to BRAFi.**

(**A**) Dose-response curves of indicated cell lines, obtained by Incucyte analysis after 72 hours of treatment with indicated doses of Vemurafenib. BiR (-) cells were cultured in the absence of Vemurafenib for 3 weeks prior to treatment with the indicated drug doses to assess for irreversible BRAFi resistance while BiR (+) cells were maintained with 1.6 µM Vemurafenib; S = Vemurafenib-sensitive cells. IC50 values are displayed within each panel. (**B**) Quantification of western blot bands intensity relative to Fig 3E, each value was normalized on DMSO treated sensitive (S) cells of the same lineage. Bar graphs represent the mean values of 3 independent experiments ± SEM. **p* < 0.05, ***p* < 0.01, ****p* < 0.001, *****p* < 0.0001, two-way ANOVA followed by Sidak’s multiple comparisons test. (**C**) qRT-PCR analysis of RICTOR gene expression performed on M14, A375 and SK-MEL-28 BiR cell lines treated for 72 hours with 1.6 µM Vemurafenib (Vem) or vehicle control (DMSO). Bar graphs represent mean values of 3 independent experiments ± SEM. ns = not significant, unpaired t-test. (**D**) WB analysis of indicated BiR cell lines transduced with scramble shRNA (shC) or with a RICTOR-targeting shRNA (shR1). Cells were analyzed after 24 hours of serum starvation (-) or 24 hours of serum starvation followed by 15 minutes of refeeding (+). Cells were kept in presence of 1.6 µM Vemurafenib. (**E**) CFE assay of indicated cell lines cultured for 12 days in presence of vehicle control (DMSO), 1.6 µM Vemurafenib (Vem), 1 µM UO126 (UO126) or the combination of 1.6 µM Vemurafenib + 1 µM UO126 (Vem + UO126).  Bar graphs represent the mean values of 3 independent experiments ± SEM. ns = not significant, **p* < 0.05, ***p* < 0.01, ****p* < 0.001, *****p* < 0.0001, unpaired t-test. Values were normalized on untreated shC cells for each cell lineage (*middle panel*) or for the untreated condition (DMSO) of each genotype (*right panel*). (**F**) Additional WB analysis relative to data shown in Fig 3F. *Right panel*: quantification of bands intensity by densitometric analysis of indicated protein bands. Comparison between DMSO and Vemurafenib-treated groups was performed by unpaired t-test, ***p* < 0.01, ****p* < 0.001.

**Supplementary Figure S4. Quantification of NDUFS1 and TUFM proteoforms after 2D-GE**

(**A**) qRT-PCR analysis of NDUFS1 gene performed on indicated cell lines. Bar graphs represent the mean values of 4 independent experiments ± SEM. ns = not significant, one-way ANOVA followed by Dunnett’s multiple comparisons test. (**B**) Quantification of western blot bands intensity, each value was normalized on shC cells of the same lineage analyzed on the same membrane. Bar graphs represent the mean values of 6 independent experiments ± SEM. ns = not significant, one-way ANOVA followed by Dunnett’s multiple comparisons test. (**C, D**) Quantification of individual proteoforms of NDUFS1 (**C**) and TUFM (**D**) after 2D-GE separation followed western blot analysis on indicated cell lines.

**Supplementary Figure S5. Supporting material relative to NAMPT expression and functions.**

(**A**) NAD^+^ of M14 cells treated for 24 hours with 5 µM Vemurafenib (Vem) or vehicle control (DMSO). Results represent the mean values of independent experiments ± SEM (n = 2). ns = not significant, two-way ANOVA followed by Tukey’s multiple comparisons test. (**B**) Quantification of individual proteoforms of NAMPT after 2D-GE separation followed western blot analysis on indicated cell lines. (**C**) Growth curves of M14 shC (*left panel*) or shR1 (*right panel*) xenografts used for tumor weight measurement, each graph refers to the tumors of the corresponding genotype treated with indicated drugs. *p < 0.05, ****p* < 0.001, *****p* < 0.0001, two-way ANOVA followed by Tukey’s multiple comparisons test performed at the experimental endpoint.

**Supplementary Figure S6. Silver staining gels used for 2D proteomic analysis.**

(**A**) Representative image of silver staining used in 2D proteomic analysis of M14 shC and shR1 cells. Numbers of spots indicated by arrows correspond to Spot n° column present in Table 1 and Supplementary Table S2.

**Supplementary Table S1. Complete GSEA analysis of metastatic SKCM TCGA samples**

Full list of Gene Ontology (GO) categories obtained from Gene Set Enrichment Analysis (GSEA) ranked for Normalized Enrichment Score (NES) based on correlation with RICTOR expression used to generate the dotplot shown in **Fig 1G,H**. **“**all”, “mut” and “wt” sheets contain GO found in the respective categories.

**Supplementary Tables S2 and S3. Extended table of proteomic results shown in Table 1 and Table 2.**

Indicated are: spot number, protein names of the identified spots by MALDI-ToF MS, UniProt Entry name, gene name and accession number (AC), the p-value determined by one-way ANOVA Test, the means of the Volumes of single spots/Volume of total spots quantified by densitometric analysis of specific spots in shC and shR cells, the fold change obtained by the ratio between the mean value of each spot, the isoelectric point (pI) and the molecular weight (MW) of each protein. Last part of the table is dedicated to Mascot Search Results for protein identification by Peptide Mass Fingerprint (PMF). Columns report Score, the expected p-value, matched peptides, and sequence Coverage (%) of the identified proteins.

**Supplementary Table S4. Gene Ontology terms emerged from proteomic analyses.**

Full list of Gene Ontology (GO) categories used to generate the bar graphs represented in **Fig 4A**. “M14 All” and “A375 All” sheets contain the full list of significantly enriched ontologies based on proteins identified in the respective cell lines. “M14 non redundant go” and “A375 non redundant go” sheets contain the of GO after filtering terms including identical sets of proteins, highlighted with different colors in the corresponding sheets.
