# Supplementary material for: RICTOR/mTORC2 downregulation in BRAFV600E melanoma cells promotes resistance to BRAF/MEK inhibition
Source: Mol Cancer. 2024 May 16;23:105. doi: 10.1186/s12943-024-02010-1 (PMC11097536; doi:10.1186/s12943-024-02010-1)
Supplement: Supplementary file 12 — Supplementary Material 12 [file 12943_2024_2010_MOESM12_ESM.docx]

**SUPPLEMENTARY MATERIALS AND METHODS**

**RICTOR downregulation with shRNA and siRNA**

Stable RICTOR-silenced cell lines were generated by lentiviral transduction of two independent RICTOR-targeting shRNAs (Addgene #1853 and #1854), while control shC cells were generated by lentiviral transduction of scramble shRNA (Addgene #1864) of the same cells of origin. Target cells (M14, A375, SK-MEL-28) were incubated with lentiviral vector-containing supernatant supplemented with Sequa-brene (Sigma-Aldrich) at a final concentration of 8 μg/mL. After 20 hours incubation, lentiviral vector-containing supernatant was substituted with fresh growth medium, and the following day selection antibiotic was added (Puromycin, 2 μg/ml). Transduced cells were expanded under continuous antibiotic selection, and all experimental procedures were carried out in the absence of Puromycin.

Transfection of RICTOR targeting (#L-016984-00, Horizon Discovery, Cambridge, UK) or non-targeting pool (#D-001810-10, Horizon Discovery) was performed using Lipofectamine 3000 (Invitrogen) according to manufacturer’s instructions. Briefly, 250000 cells were seeded in 60 mm dishes at day 1 and after 24 hours were transfected using Lipofectamine 3000 reagent and 0,7 µmoles of each siRNA. 24 hours post-transfection cells were trypsinized and plated in 6-well plates (2000 cells/well) for Colony Forming Efficiency, or for Western Blot analysis.

**Western blotting**

Cells seeded on culture plates were frozen in liquid nitrogen before lysis. Cells were scraped on ice with cold lysis buffer (1% Triton X-100, 50 mM Tris/HCl pH 7.4, 150 mM NaCl, 1mM EDTA) supplemented with 1 mM PMSF, 1 mM Na_3_VO_4_, 10 mM NaF, 1X cOmplete Protease Inhibitors Cocktail (Roche, Basel, Switzerland) and 10 mM sodium butyrate. Lysates were centrifuged at 14000g for 15min at 4°C and protein concentration was measured using the Bradford assay (Bio-Rad, Hercules, CA, USA). Lysates were diluted in Laemmli buffer and boiled for 10 minutes at 95°C. Equal amounts of proteins were loaded on 4–15% Mini- PROTEAN® TGX™ Precast Protein Gels (Bio-Rad) and transferred to PVDF Transfer Membrane (Merck Millipore, Billerica, MA, USA). Membranes were blocked in 5% non-fat dry milk (Santa Cruz Biotechnology, Dallas, TX, USA) in Tris-buffer saline, 0.1% Tween20 and incubated with the indicated antibodies following the manufacturer’s instructions.

The following antibodies were used: Rictor (#9476), Sin1 (#12860), Phospho-Akt (Ser473) (#4060), Phospho-NDRG1 (Thr346) (#5482), Phospho-p44/42 MAPK (Erk1/2) (Thr202/Tyr204) (#4370), p44/42 MAPK (Erk1/2) (#4695), Phospho-MEK1/2 (Ser221) (#2338), MEK1/2 (#9122), Phospho-p90RSK (Thr359/Ser363) (#9344), NDUFS1 (#70264), PBEF/NAMPT (#61122), Phospho-RB (S807/811) (#8516), SOX10 (#D5V9L), AXL (#8861), Ubiquitin (#3936) from Cell Signaling Technology (Danvers, MA, USA); GAPDH (AM4300) from Invitrogen (Waltham, MA, USA); TUFM (#MA5-31364) from Thermo Fisher (Waltham, MA, USA); MITF (#NBP1-88618) from Novus Biologicals (Centennial, CO, USA); Horseradish peroxidase-conjugated secondary antibodies from Sigma-Aldrich. Immunoblots were developed by chemiluminescence with ECL (Clarity Western ECL Substrate, Bio-Rad), acquired with the molecular imager ChemiDoc XRS, and quantified by densitometric analysis using the Image-lab software (Bio-Rad). All comparative images of immunoblots were obtained by exposure of the same membranes.

**Seahorse Metabolic Experiments**

Real-time measurements of oxygen consumption rate (OCR) were made using an XF Extracellular Flux Analyzer (Agilent Technologies, Santa Clara, CA). Cells were seeded in XFe96 plates (Agilent) at 30000 cells/well and OCR was measured using the XFe96 Extracellular Flux Analyzer and the XF Cell Mito stress test kit (Agilent) according to the manufacturer’s instructions. Cells in XF Mito stress media (DMEM supplemented with 10 mM glucose, 2 mM glutamine and 1 mM pyruvate, pH 7.4) were incubated at 37 °C in the absence of CO_2_ for 1 h. Baseline OCR measurements were determined before administration of oligomycin (1 mM), FCCP (1 mM), and a combination of rotenone and antimycin A (0.5 mM). OCR data were obtained and analyzed using the XF Cell Mito Stress Test Generator software (Agilent Seahorse Bioscience).

**Lentiviral vector Production**

Lentiviral vectors were generated as described in (1). Briefly, 3 x 10^6^ HEK-293T cells were plated in a 10 cm cell culture dish in DMEM media supplemented with 10% FBS without penicillin/streptomycin. 24 hours post-plating, cells were incubated overnight with transfection reaction consisting of Lipofectamine 2000 (Invitrogen) and DNA plasmids, both diluted in Opti-MEM (Thermo-Fisher Scientific) following the manufacturer’s instructions. The following plasmids were used: pMD2-VSV-G (envelope plasmid), pCMV-dR8.74 (packaging plasmid) and transfer plasmid (pLKO.1-scramble/Rictor_1/Rictor_2 shRNA). After overnight incubation, the supernatant was removed and replaced with fresh medium. Lentivirus-containing supernatant was collected after 48 hours, filtrated (0.22 µm pore), aliquoted and stored at -80°C until use.

**Reagents**

Vemurafenib, UO126, FK866 used for *in vitro* experiments were purchased from Selleckchem (Houston, TX, USA), OT-82 was purchased from Selleckchem, Bortezomib was purchased from Sigma-Aldrich (St. Louis, MO, USA). Vemurafenib and FK866 used for *in vivo* experiments were purchased from MedChem Express (Monmouth Junction, NJ, USA). Phenformin hydrochloride was purchased from Sigma-Aldrich (St. Louis, MO, USA).

For *in vivo* experiments, FK866 was diluted in an aqueous solution containing 30% propylene glycol, 5% Tween-80 and 5% dextrose (all reagents from Sigma) at a concentration of 10 mg/Kg, while Vemurafenib was diluted in an aqueous solution containing 21% DMSO, 30% PEG-400, 0.5% Tween-80 and 5% propylene glycol (all reagents from Sigma) at a concentration of 30 mg/Kg.

**Determination of drug sensitivity**

Cells were seeded into 96-well plates (2500 cells/well) and treated with the indicated drug doses of Vemurafenib the following day. The plates were placed in an IncuCyte SX5 Live-Cell Analysis System (Essen BioScience, Ann Arbor, Michigan, USA) for 72 hours, with images acquired every 4 hours. Cell confluence was measured and quantified by the IncuCyte imaging system (Essen Bioscience). IC50 values were calculated at the endpoint using the online tool Quest Graph™ IC50 Calculator (AAT Bioquest, Inc., Sunnyvale, CA, USA).

**Growth assay**

Cells were seeded (M14 40000 cells/well; A375 30000 cells/well) into 12-well plates (one plate for each time point) and media change was performed the following day. 24, 48, 74 and 96 hours after media change, cells were fixed with 4% paraformaldehyde for 15 minutes, rinsed with PBS and stained with 0.1% crystal violet. Staining intensity was quantified by dissolving crystal violet with 250 µl of 10% acetic acid for 15 minutes, then 100 µl were moved in 96-well plates and optical density (OD) was measured by 560nm absorbance using Promega GloMax Explorer GM3500.

**NAMPT activity measurement**

The assay allows determination of NAMPT activity by converting NMN to NAD^+^, via three consecutive reactions consisting of NMN deamidation to NaMN, NaMN adenylyation to NaAD, and NaAD conversion to NAD^+^. The reactions are catalyzed by the bacterial recombinant enzymes NMN deamidase (PncC), NaMN adenylyltransferase (NadD) and NAD synthetase (NadE). The formed NAD^+^ is finally quantitated by a fluorometric cycling assay (2).

Briefly, cells (2x10^7^) were washed twice with PBS, and cell pellets were resuspended in 200 µl of 50 mM TRIS/HCl, pH 7.5, 0.15 M NaCl, 1 mM DTT, 1 mM PMSF, 0.002 mg/ml leupeptin, antipain, chymostatin and pepstatin. The suspension was sonicated three times for 1 min at 50 W, with 1 min intervals, and centrifuged at 7000 rpm for 15 min at 4°C. The supernatants were immediately used, and the protein concentration was measured using the Bradford assay (Bio-Rad, Hercules, CA, USA).

The assay mixture contained ethanol buffer (30 mM HEPES, pH 8.0, 1 % v/v ethanol, 8.4 mg/ml semicarbazide), 40 mM HEPES/KOH, pH 7.5, 10 mM KF, 10 mM MgCl_2_, 2.5 mM ATP, 0.05 mM Nam, 1.0 mM PRPP, 6 Units/ml ADH (Sigma A3263), 0.067 mg/ml BSA, 1 Units /ml NadD, 0.03 Units /ml PncC, and 0.25 mg/mL of cell extracts, in a final volume of 135 µL. A control mixture in the presence of 5 μM FK866 was also processed in parallel. Reaction mixtures were incubated at 37°C, and at suitable time intervals, 30 μl aliquots were withdrawn and added with half their volume of 1.2 M cold HClO_4_ to stop the reaction. After 15 minutes on ice, samples were centrifuged (20000 g, 5 minutes), and supernatants were neutralized with 1 M K_2_CO_3_. After centrifugation, 40 μl aliquots were transferred into a flat-bottom 96-well black plate. NaAD conversion to NAD^+^ was started by adding 50 mM HEPES, pH 7.5, 0.15 M KCl, 1.4 mM ATP, 50 mM NH_4_Cl, 11 mM MgCl_2_ and 0.06 U/ml NadE, to a final volume of 145 μl. After incubation (30 minutes, 37 °C) the NAD^+^ cycling reaction was started by adding 96 μl of cycling reagent to each well. The cycling reagent, freshly prepared, contained 100 mM sodium phosphate, pH 8.0, 2% ethanol, 32 µM resazurin, 10 U/mL ADH, 0.1 mg/mL BSA, 10 µM flavin mononucleotide and 0.1 mg/mL diaphorase that was previously purified through a PD MiniTrap Sephadex G-25 column (GE Healthcare) equilibrated and eluted with 10 mM sodium phosphate buffer, pH 8.0. Following addition of the cyclic reagent, the resorufin fluorescence was measured continuously in each well using a Synergy HT microplate reader (Bio-Tek, Winooski,VT, USA) equipped with 544 and 590 nm excitation and emission filters, respectively. The amount of NaAD present in the wells, corresponding to the amount of NMN formed by the enzyme, was calculated by interpolating on a NaAD standard curve the corresponding rate of fluorescence increase, after subtracting the respective control. The results were expressed as pmoles of product formed /hour/µg protein and are means ± standard deviation of two independent experiments. The ancillary bacterial enzymes PncC, NadD, and NadE were prepared as described (2).

**NAD^+^ level measurement**

Pellets from about 3x10^5^ cells were resuspended in 0.15 mL of 0.4 M cold HClO_4_. After centrifugation at 2300 g for 10 min at 4°C, the supernatants were neutralized with 1.0 M K_2_CO_3_. Neutralized samples were then centrifuged as described above and pellets were resuspended in 0.1 mL formic acid for protein determination using the Bradford assay. NAD^+^ was measured in the supernatants through the fluorometric cycling assay described in the previous section, by adding a suitable amount of sample diluted in 0.145 mL of water to 96 μl of cycling reagent. The increase in resorufin fluorescence was measured as described. The amount of NAD^+^ was calculated by interpolation on a NAD^+^ standard curve and the results were expressed as nmol NAD^+^/mg protein. Data are means ± standard deviation of two independent experiments.

**RNA isolation and qRT-PCR**

RNA was isolated from cells using TRIzol reagent (Invitrogen) according to the manufacturer’s instructions and converted to cDNA using the High Capacity cDNA Reverse Transcription kit (Thermo Fisher Scientific). qRT-PCR was performed using the 7900 HT Fast Real Time PCR system (SDS2.3 software) using commercially available primers (TaqMan Gene Expression Assays; Thermo Fisher Scientific): Hs00237184_m1 (*NAMPT*), Hs00192297_m1 (*NDUFS1*), Hs00380903_m1 (RICTOR), Hs99999903_m1 (*ACTB*, used ad housekeeping gene). Comparative CT methods was used to calculate the relative expression of the gene under analysis.

**2D Gel electrophoresis**

2D-Electrophoresis (2DE) was performed using the Immobiline polyacrylamide system (3). Immobilized nonlinear pH 3-10 gradient on strips 18 cm in length (Cytiva, Uppsala, Sweden) (formerly GE Healthcare) were employed in the first dimensional run carried out by Ettan™ IPGphor™ Manifold (GE Healthcare, Uppsala, Sweden) at 16 °C with the following electrical conditions: 200 V for 8 h, from 200 V to 3500 V in 2 h, 3500 V for 2 h, from 3500 V to 5000 V in 2 h, 5000 V for 3 h, from 5000 V to 8000 V in 1 h, 8000 V for 3 h, from 8000 V to 10000 V in 1 h, 10000 V for 10 h for a total of 90,000 VhT. Mass Spectrometry (MS)-preparative strips were pre-rehydrated overnight with 350 µl of denaturing solution. Samples added with 0.2% of carrier ampholytes for the analytical runs and 2% for the preparative ones, were loaded by rehydration loading and cup at the cathodic ends of the IPGstrips, respectively. At the end of the first dimensional run, strips were washed with deionized water and equilibrated with two buffers: the first composed of 6 M Urea, 2% w/v Sodium Dodecyl Sulphate (SDS), 2% w/v DTE, 30% v/v glycerol and 0.05 M Tris-HCl pH 6.8, for 12 min; the second one composed of 6 M Urea, 2% w/v SDS, 2.5% w/v Iodoacetamide, 30% v/v glycerol, 0.05 M Tris-HCl pH 6.8 and a trace of bromophenol blue, for 5 min. The second dimension was then performed at 40 mA/gel constant current on 9-16% SDS polyacrylamide linear gradient gels (size: 18 x 20 cm x 1.5 mm) at 9°C. Analytical gels were stained with ammoniacal silver nitrate, while preparative gels underwent a mass spectrometry-compatible silver staining (4); then, gels were digitized with Image Scanner III laser densitometer supplied with the LabScan 6.0 software (GE Healthcare). 2D image analysis was performed using Melanie 9 software (Geneva Bioinformatics-GeneBio, Geneva, Swiss). Gel comparison highlighted quantitative and qualitative protein differences, validated by a statistical analysis. By Melanie 9 software, the ANOVA test was applied to compare the percentage of relative volume (%V) of the 2DE protein spots among the groups (ShC vs ShR). Particularly, only spots with a p-value ≤ 0.05 and a fold change at least of 1.5 in the %V means ratio, were considered differentially abundant.

**Protein Identification by MALDI-ToF Mass Spectrometry**

Differential proteins found were identified via MALDI-ToF MS using peptide mass fingerprinting (PMF). Differential spots were manually excised from MS-compatible silver-stained gels. Spots were destained first in a solution of 30 mM potassium ferricyanide and 100 mM sodium sulphate anhydrous, and then later in 200 mM ammonium bicarbonate. Then, they were dehydrated in 100% acetonitrile (ACN). Protein spots were rehydrated and digested overnight at 37 °C in a trypsin solution. Digested proteins were then placed on a MALDI target, dried, and covered with a matrix solution of 5 mg/mL α-cyano-4-hydroxycinnamic acid (CHCA) in 50% *v*/*v* ACN and 0.5% *v*/*v* trifluoroacetic acid (TFA). The MS analysis was carried out using the UltrafleXtreme™ MALDI-ToF/ToF mass spectrometer (Bruker Daltoniks, Bremen, Germany), equipped with a 200 Hz smartbeam™ I laser in the positive reflector mode with the following parameters: 80 ns of delay; ion source 1: 25 kV; ion source 2: 21.75 kV; lens voltage: 9.50 kV; reflector voltage: 26.30 kV; and reflector 2 voltage: 14.00 kV. The applied laser wavelength and frequency were 353 nm and 100 Hz, respectively, and the percentage was set to 50%. Final mass spectra were produced by averaging 1500 laser shots targeting five different positions within the spot. MS spectra were acquired and processed via the FleXanalysis software version 3.0 (Bruker) using peptides arising from trypsin autoproteolysis as the internal standard for calibration. The resulting mass lists were filtered for common contaminants such as matrix-related ions, trypsin autolysis, and keratin peaks. Protein identification was carried out by utilizing the peptide mass fingerprinting search using MASCOT (Matrix Science Ltd., London, UK, <http://www.matrixscience.com>; accessed on 2 May 2022); setting up the following parameters: Homo sapiens as taxonomy, SwissProt as database, 20 ppm as mass tolerance, one admissible missed cleavage site, and carbamidomethylation (iodoacetamide alkylation) of cysteine as fixed modification and oxidation of methionine as a variable modification. Only protein identifications with a *p*-value < 0.04, a minimum of four matched peptides and a minimum MASCOT score of 55 were considered.

**TCGA data download and pre-processing**

TCGA SKCM data were downloaded through TCGAbiolinks (5). ENSEMBL gene Ids were converted to HGCN symbols with biomaRt(6), with the ENSEMBL ID having the highest average expression being kept in the case of multiple ENSEMBL IDs mapping to the same gene symbol, and data were log2 transformed with an offset of 1. Only samples annotated as metastatic were retained for further analyses.

Proteomic (RPPA) data were downloaded from the UCSC XENA database (7) and matched with the transcriptomic samples obtained from the corresponding patient.

All the TCGA data were downloaded in May 2021.

**Correlation with survival**

TCGA SKCM survival data were obtained with the GDCquery_clinic function from the TCGAbiolinks package. The overall survival of metastatic melanomas with the highest and lowest quartile in RICTOR/RAPTOR/MTOR expression were compared and displayed as Kaplan-Meier curves with the survminer package.

**Enrichment analysis**

Spearman’s correlation between RICTOR and all other genes across metastatic melanomas (TCGA, transcriptional data) were obtained and ranked to perform a GSEA with Gene Ontology, Biological Process categories. For this, the msigdbr package (8) was used to obtain the GO lists (category C5, subcategory GO:BP) and the GSEA was done with the fgsea package (9).

**Software and Plots**

All the analyses were done with R 4.0.3 (10).

Packages used for plotting: Ggpubr (11), survminer (12), ggplot2 (13).

**References**

1. Saoncella S, Tassone B, Deklic E, Avolio F, Jon C, Tornillo G, et al. Nuclear Akt2 opposes limbal keratinocyte stem cell self-renewal by repressing a FOXO-mTORC1 signaling pathway. Stem Cells. 2014 Mar;32(3):754–69.

2. Zamporlini F, Ruggieri S, Mazzola F, Amici A, Orsomando G, Raffaelli N. Novel assay for simultaneous measurement of pyridine mononucleotides synthesizing activities allows dissection of the NAD + biosynthetic machinery in mammalian cells. FEBS Journal. 2014 Nov 1;281(22):5104–19.

3. Landi C, Liberatori G, Puccini M, Shaba E, Vantaggiato L, Vitolo S, et al. Proteomics coupled with AhR-reporter gene bioassay for human and environmental safety assessment of sewage sludge and hydrochar. Science of the Total Environment. 2023 Sep 15;891.

4. Chevallet M, Luche S, Diemer H, Strub JM, Van Dorsselaer A, Rabilloud T. Sweet silver: A formaldehyde-free silver staining using aldoses as developing agents, with enhanced compatibility with mass spectrometry. Proteomics. 2008;8(23–24):4853–61.

5. Colaprico A, Silva TC, Olsen C, Garofano L, Cava C, Garolini D, et al. TCGAbiolinks: An R/Bioconductor package for integrative analysis of TCGA data. Nucleic Acids Res. 2016 May 5;44(8):e71.

6. Durinck S, Spellman PT, Birney E, Huber W. Mapping identifiers for the integration of genomic datasets with the R/Bioconductor package biomaRt. Nat Protoc. 2009 Jan;4(8):1184–91.

7. Goldman MJ, Craft B, Hastie M, Repečka K, McDade F, Kamath A, et al. Visualizing and interpreting cancer genomics data via the Xena platform. Nat Biotechnol. 2020;38(6):675–8.

8. Dolgalev I. msigdbr: MSigDB Gene Sets for Multiple Organisms in a  Tidy Data Format. R package version 7.1.1.  https://CRAN.R-project.org/package=msigdbr. 2020;

9. Korotkevich G, Sukhov V, Budin N, Shpak B, Artyomov MN, Sergushichev A. Fast gene set enrichment analysis. Available from: https://doi.org/10.1101/060012

10. R Core Team. R: A language and environment for statistical computing. Vienna, Austria: R Foundation for Statistical Computing; 2018.

11. Kassambara A. Ggpubr:“Ggplot2” Based Publication Ready Plots (Version 0.3. 0)[Computer Software]. 2020;

12. Kassambara A, Kosinski M, Biecek P. survminer: Drawing Survival Curves using “ggplot2”. R package version 0.4.8.  https://CRAN.R-project.org/package=survminer. 2020;

13. Gómez-Rubio V. ggplot2 - Elegant Graphics for Data Analysis (2nd Edition) . J Stat Softw. 2017;77(Book Review 2).
