# Supplementary material for: RICTOR/mTORC2 downregulation in BRAFV600E melanoma cells promotes resistance to BRAF/MEK inhibition
Source: Mol Cancer. 2024 May 16;23:105. doi: 10.1186/s12943-024-02010-1 (PMC11097536; doi:10.1186/s12943-024-02010-1)
Supplement: Supplementary file 13 — Supplementary Material 13 [file 12943_2024_2010_MOESM13_ESM.pdf]

Figure 2A

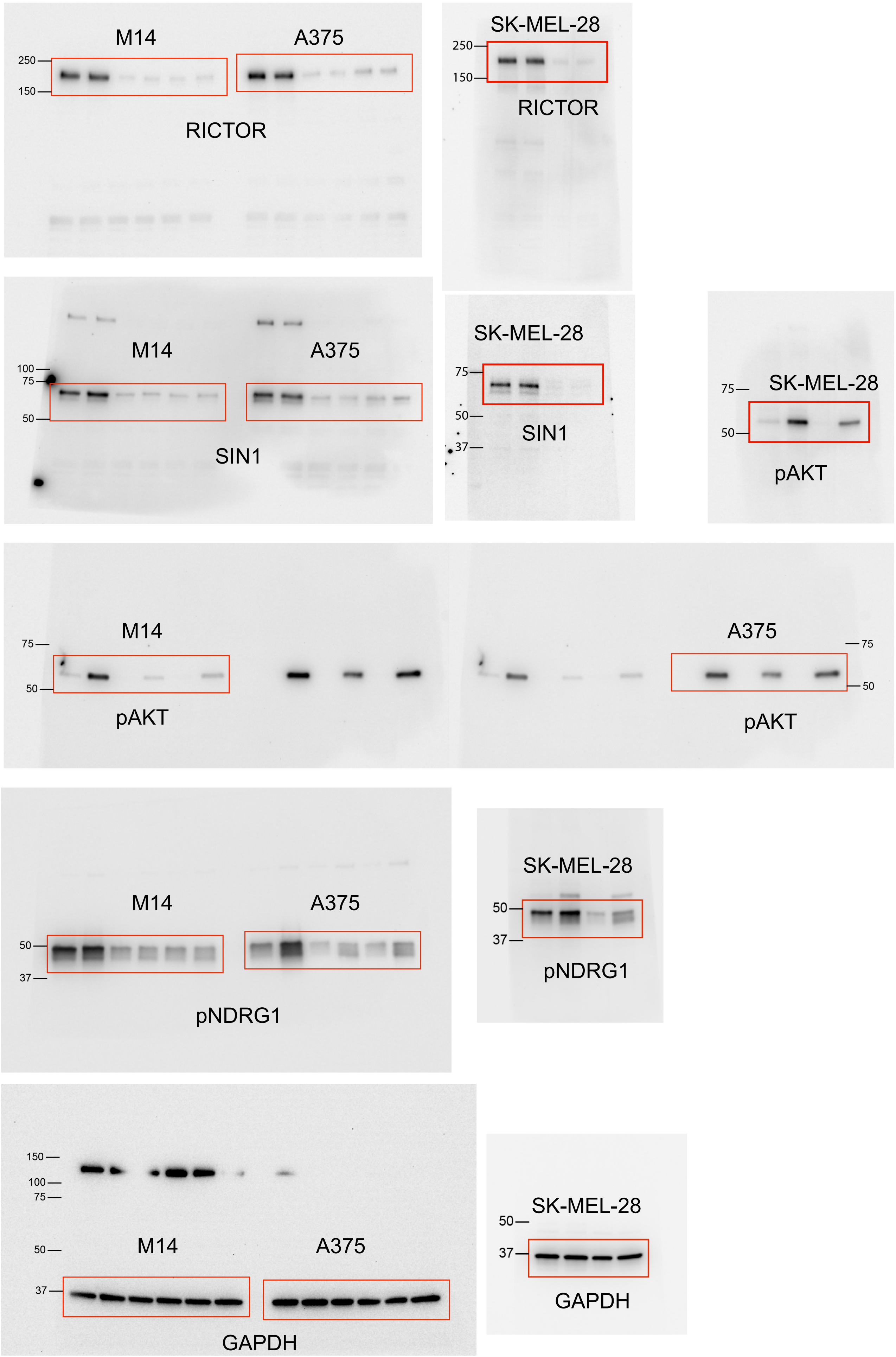
M14
A375
250
150
RICTOR
SK-MEL-28
250
150
RICTOR
M14
A375
100
75
50
SIN1
SK-MEL-28
75
50
37
SIN1
75
SK-MEL-28
50
pAKT
M14
75
50
pAKT
A375
75
50
pAKT
M14
A375
50
37
pNDRG1
SK-MEL-28
50
37
pNDRG1
150
100
75
50
M14
A375
37
GAPDH
SK-MEL-28
50
37
GAPDH

Figure 2D

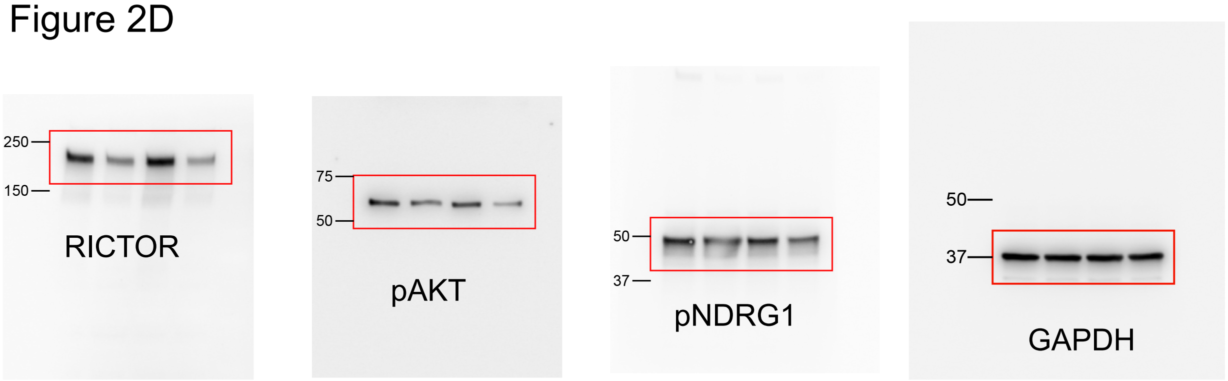

Figure 3A

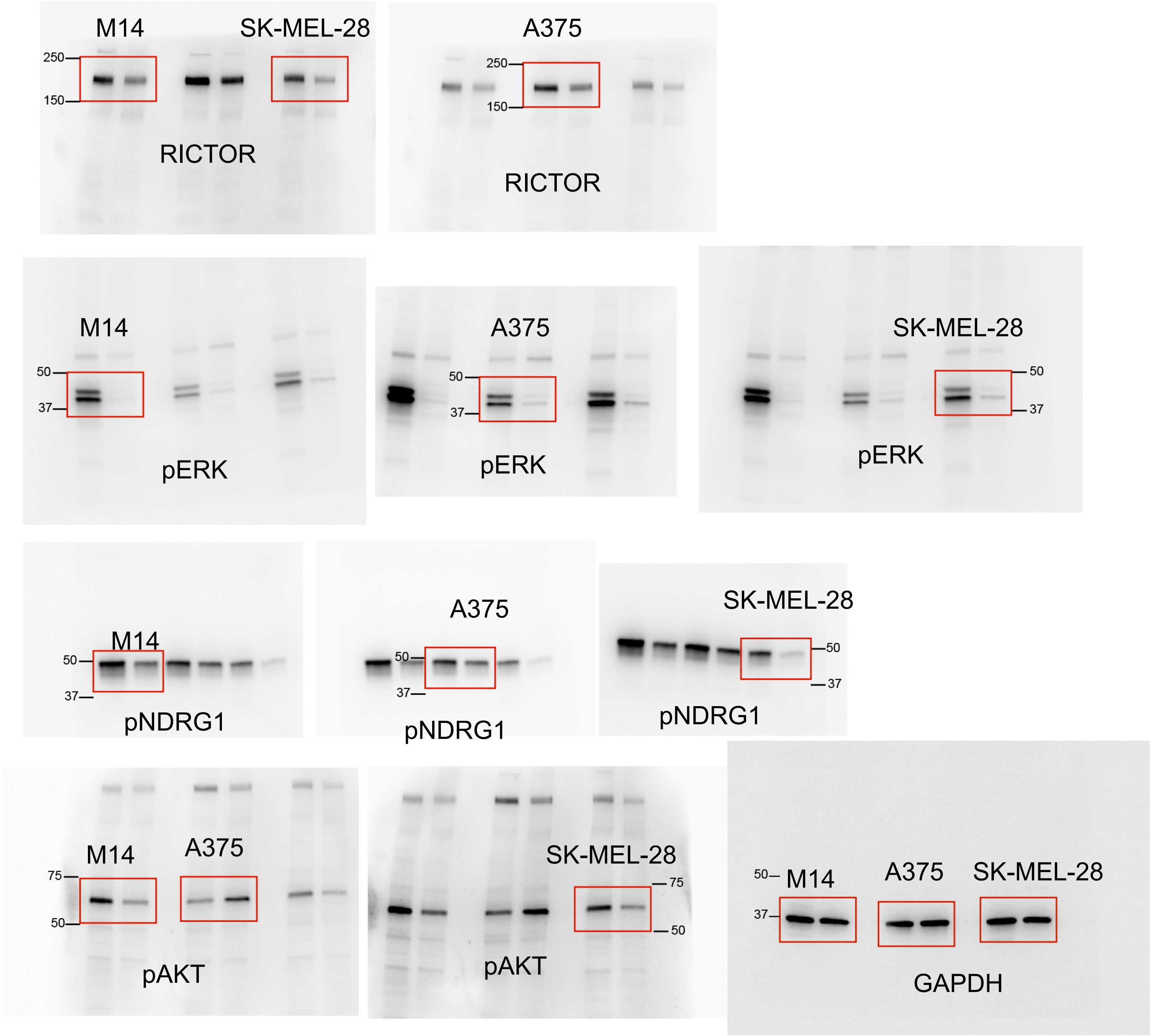

Figure 3C

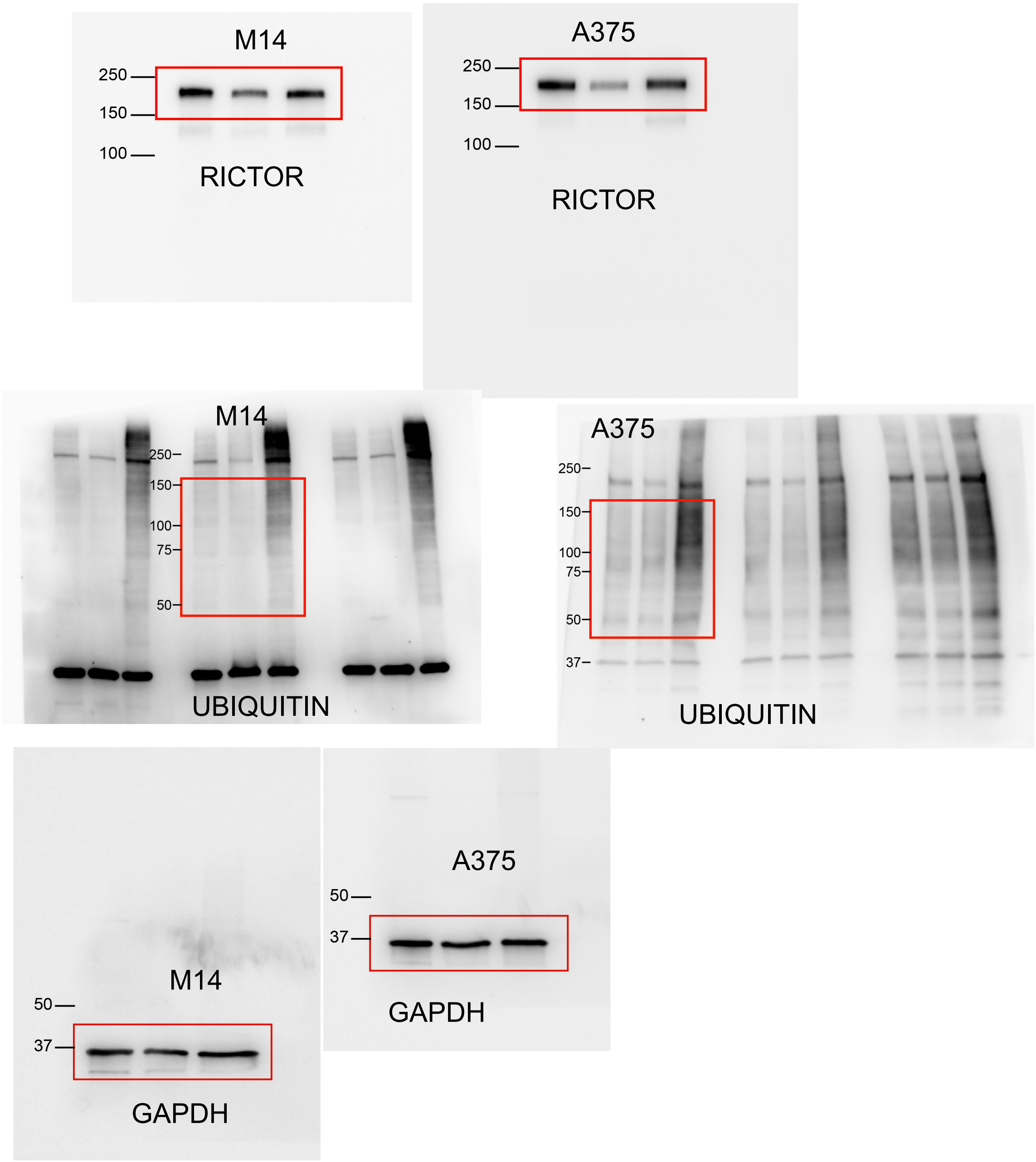

M14
250
150
100
RICTOR
A375
250
150
100
RICTOR
M14
250
150
100
75
50
UBIQUITIN
A375
250
150
100
75
50
37
UBIQUITIN
M14
50
37
GAPDH
A375
50
37
GAPDH

Figure 3D

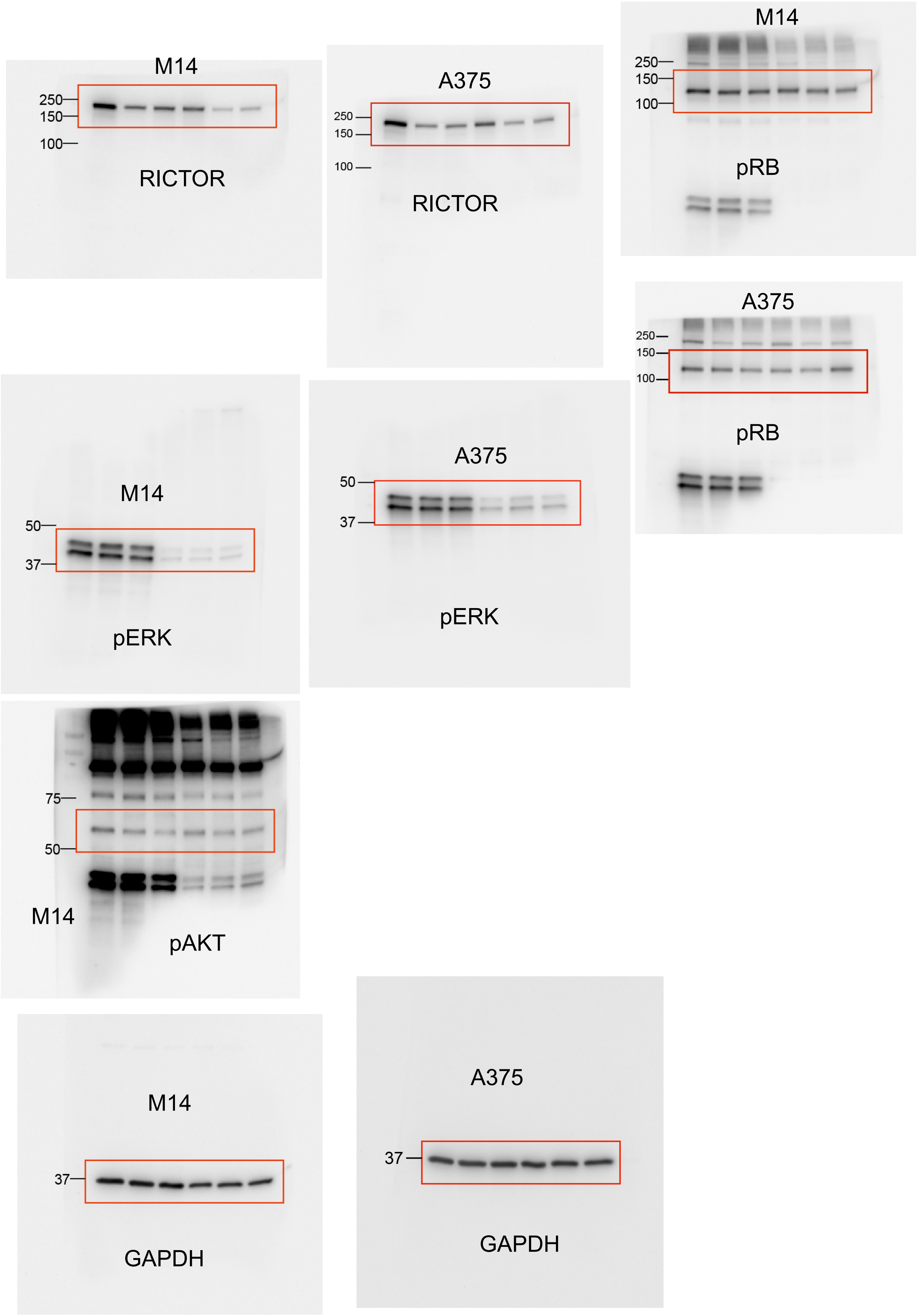
M14
250
150
100
RICTOR
A375
250
150
100
RICTOR
M14
250
150
100
pRB
A375
250
150
100
pRB
M14
50
37
pERK
A375
50
37
pERK
75
50
M14
pAKT
M14
37
GAPDH
A375
37
GAPDH

Figure 3E

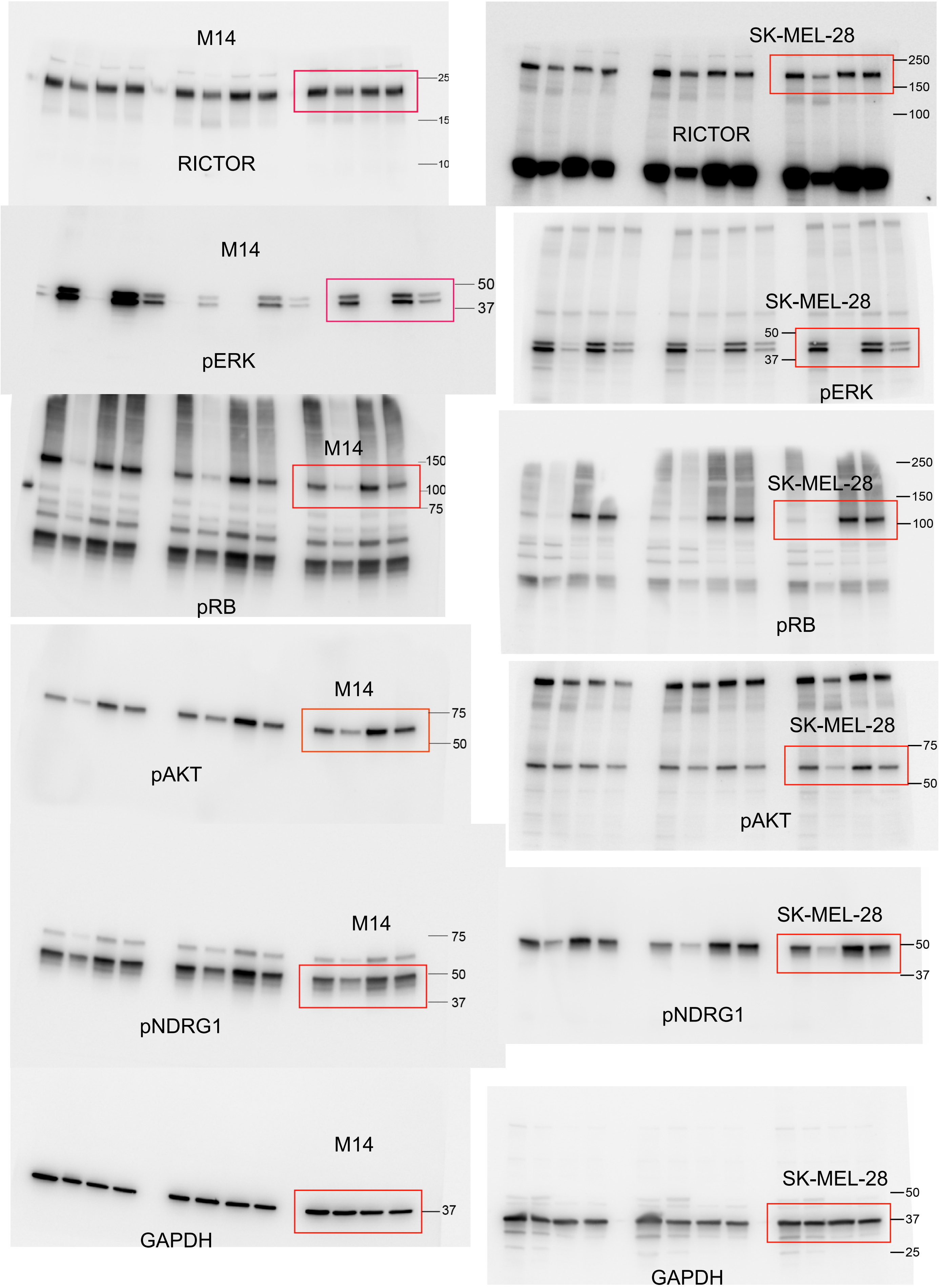

M14
25
15
10
RICTOR
SK-MEL-28
250
150
100
RICTOR
M14
50
37
pERK
SK-MEL-28
50
37
pERK
M14
150
100
75
pRB
SK-MEL-28
250
150
100
pRB
M14
75
50
pAKT
SK-MEL-28
75
50
pAKT
M14
75
50
37
pNDRG1
SK-MEL-28
50
37
pNDRG1
M14
37
GAPDH
SK-MEL-28
50
37
25
GAPDH

Figure 3E

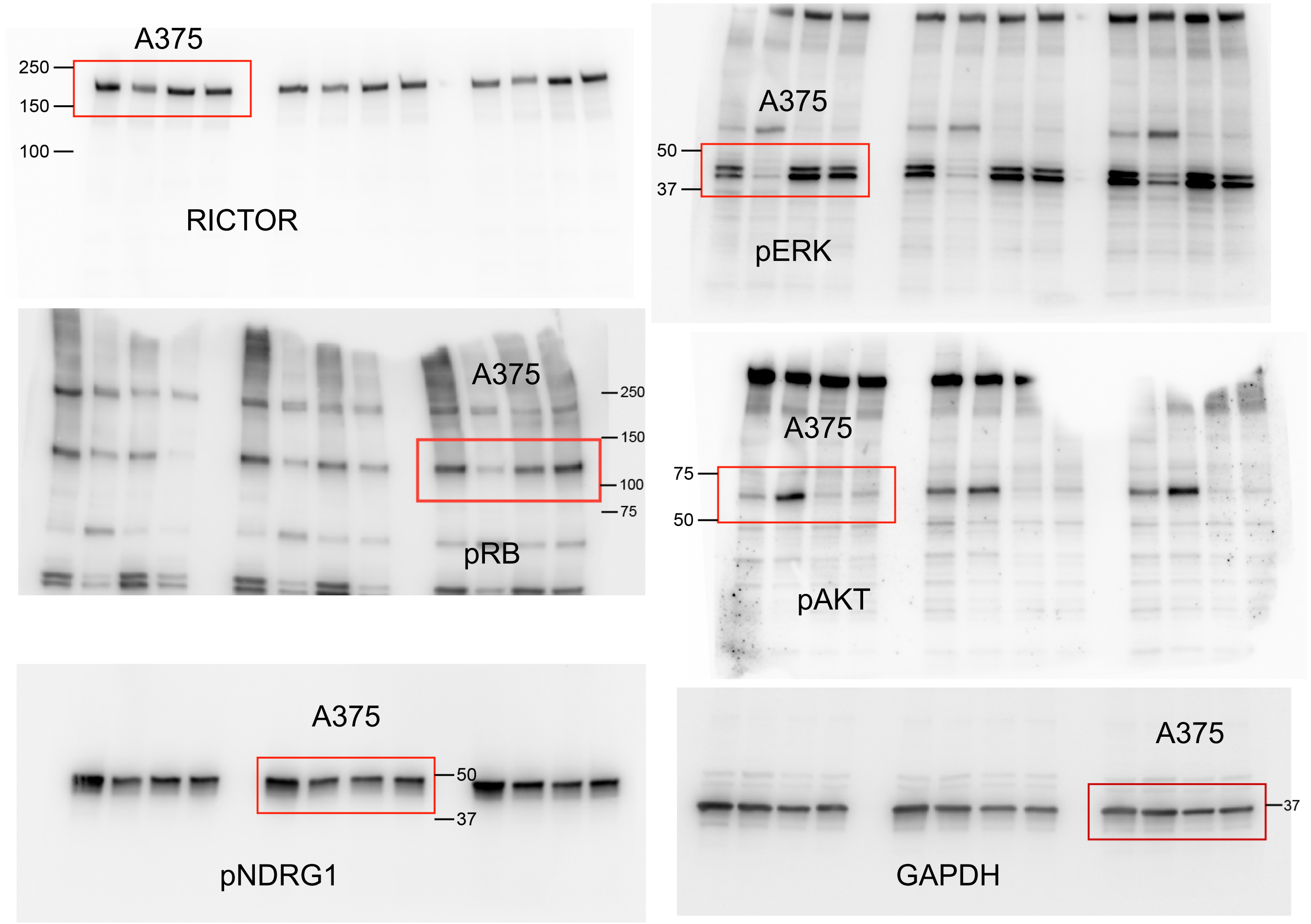

Figure 3F

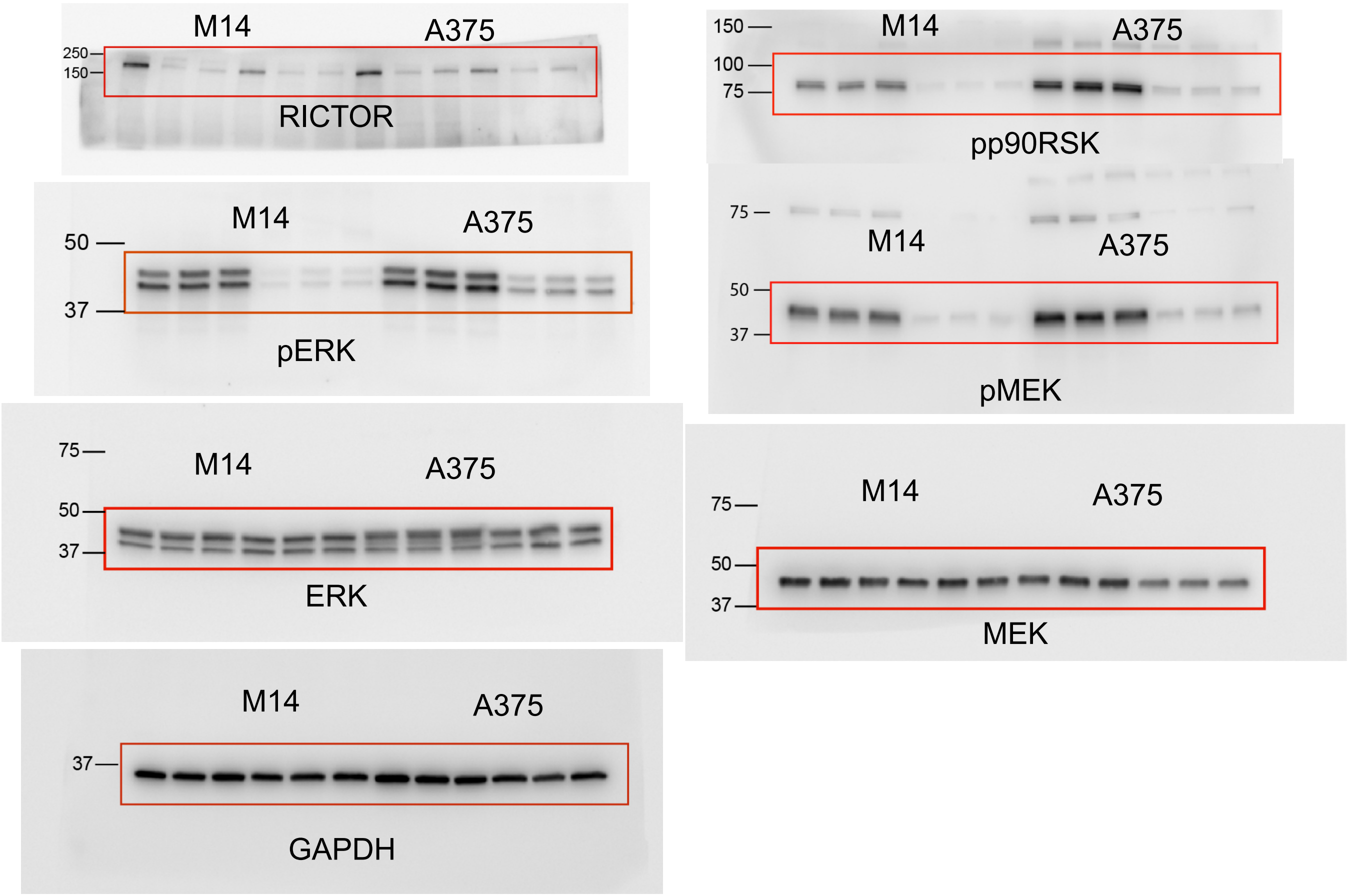

Figure 4C

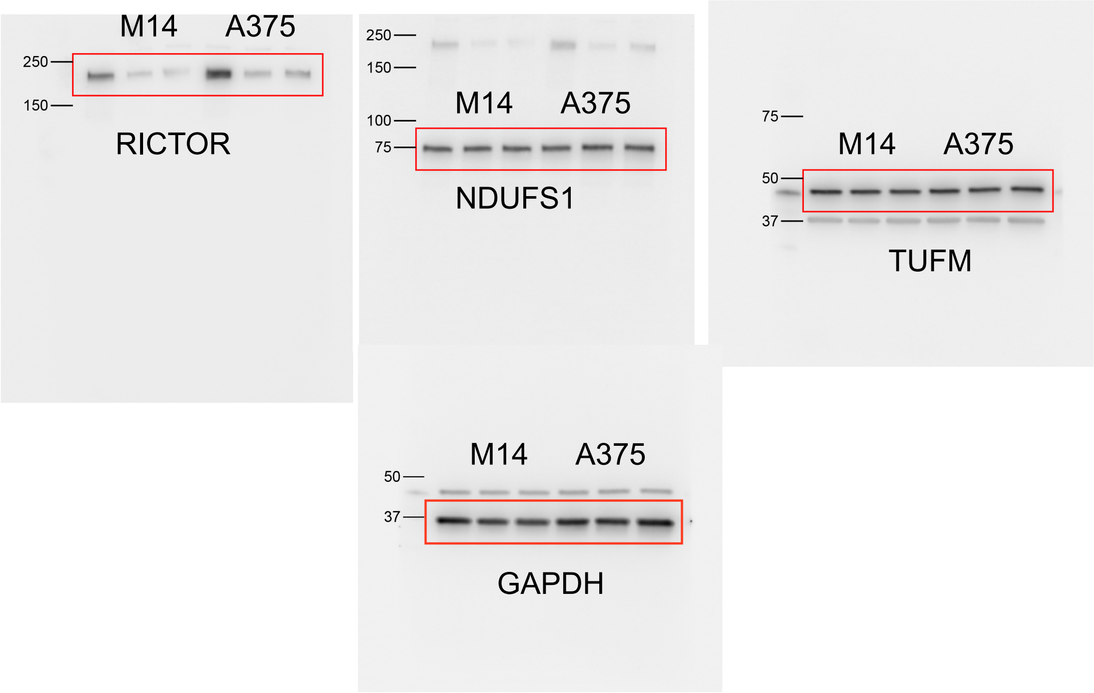

Figure 4E

TUFM

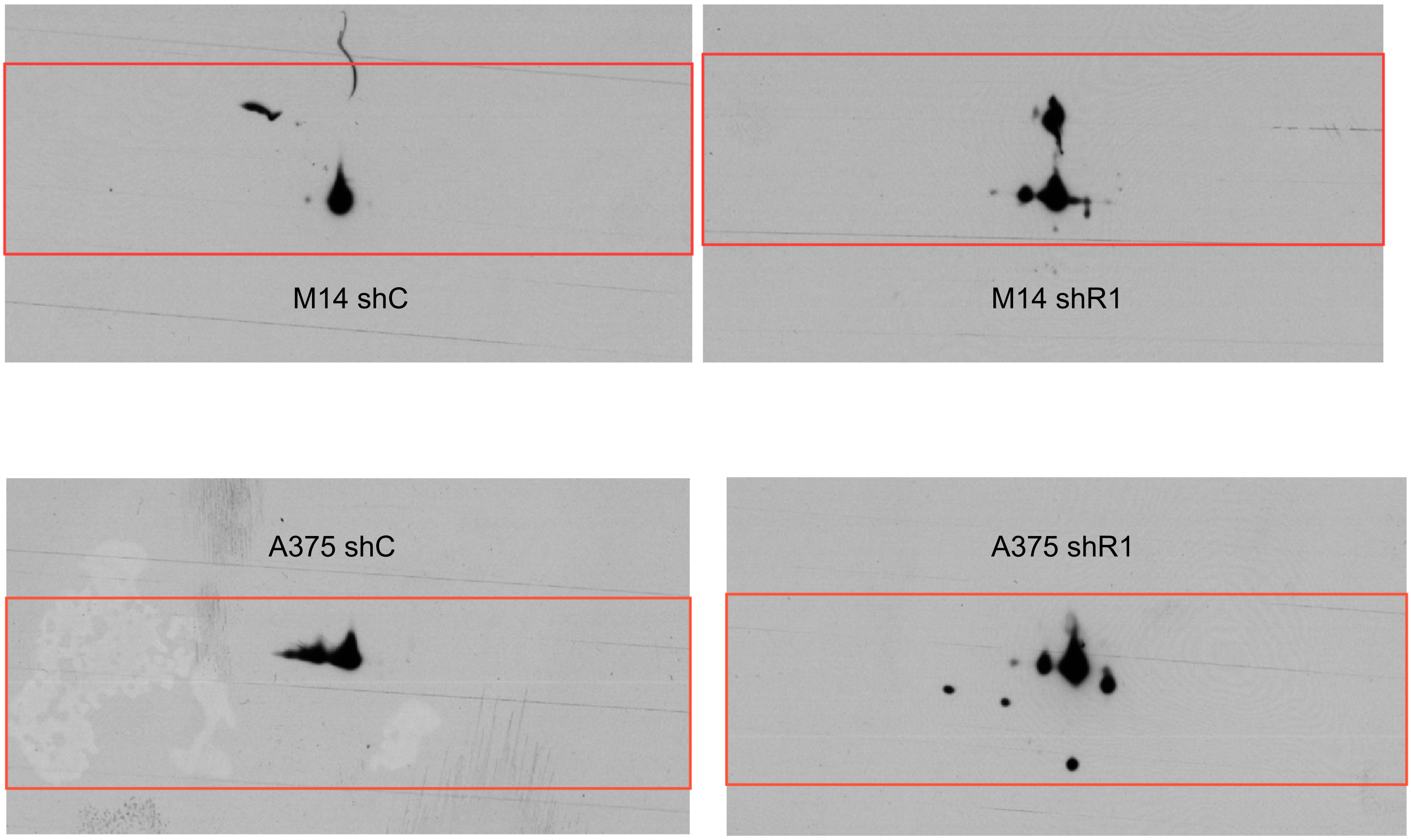

Figure 4D NDUFS1

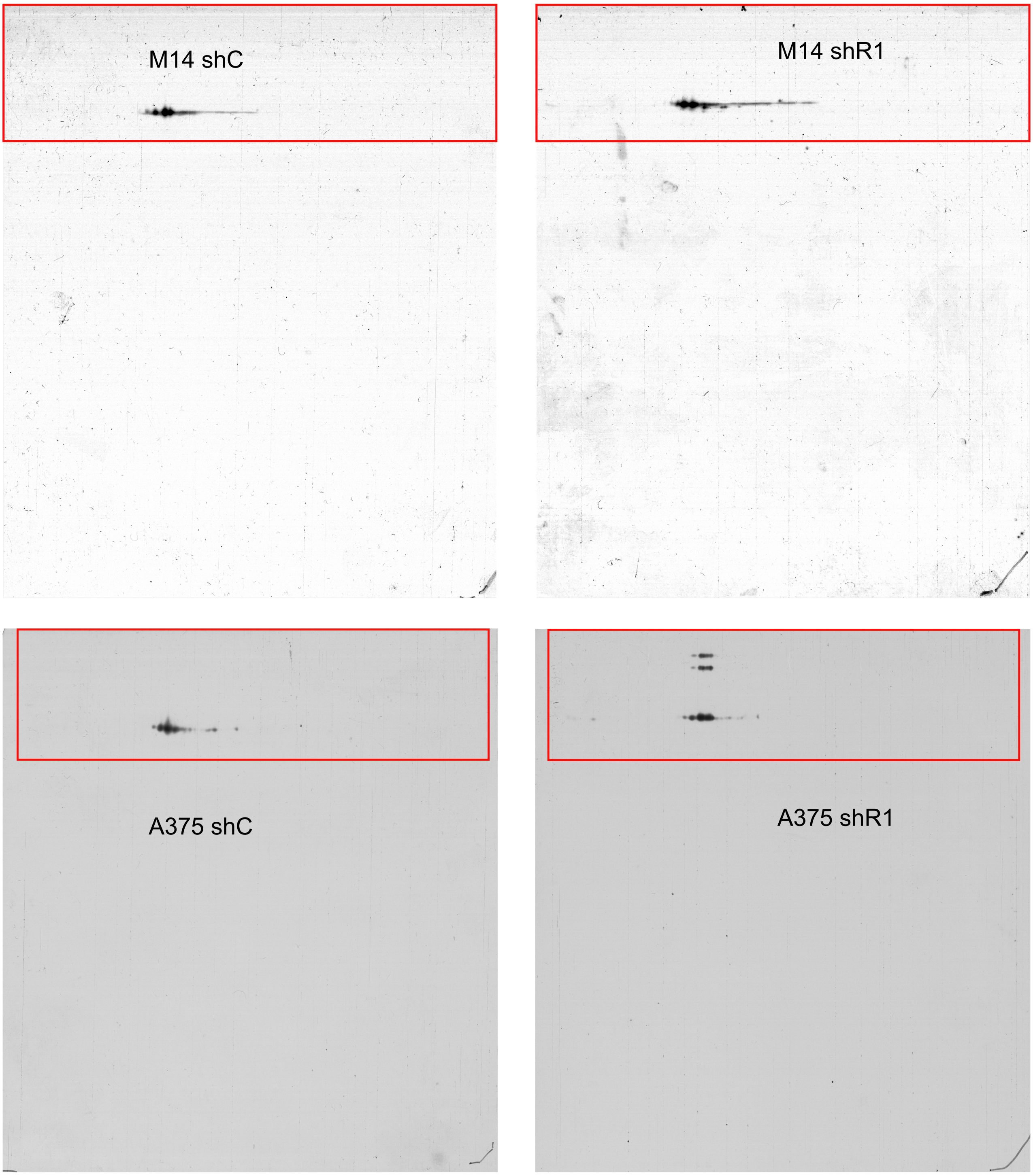

Figure 5A

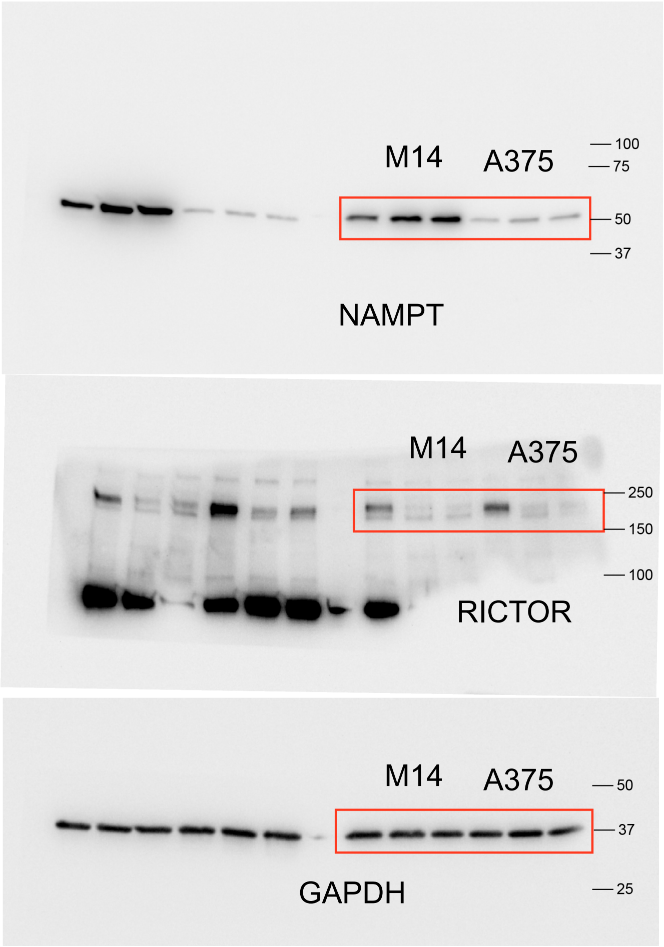
M14
A375
100
75
50
37
NAMPT
M14
A375
250
150
100
RICTOR
M14
A375
50
37
GAPDH
25

Figure 5D NAMPT

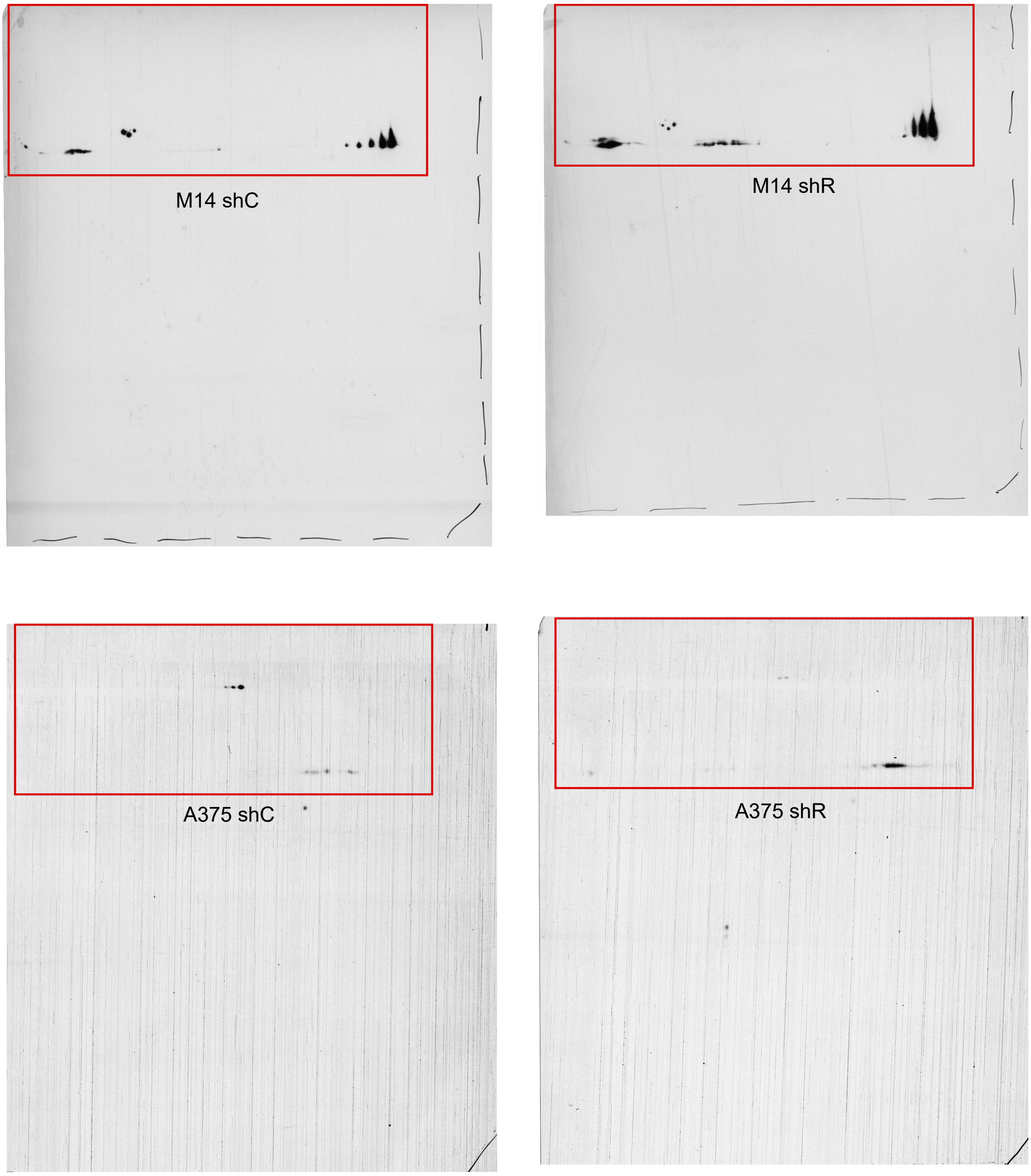

Figure S3D

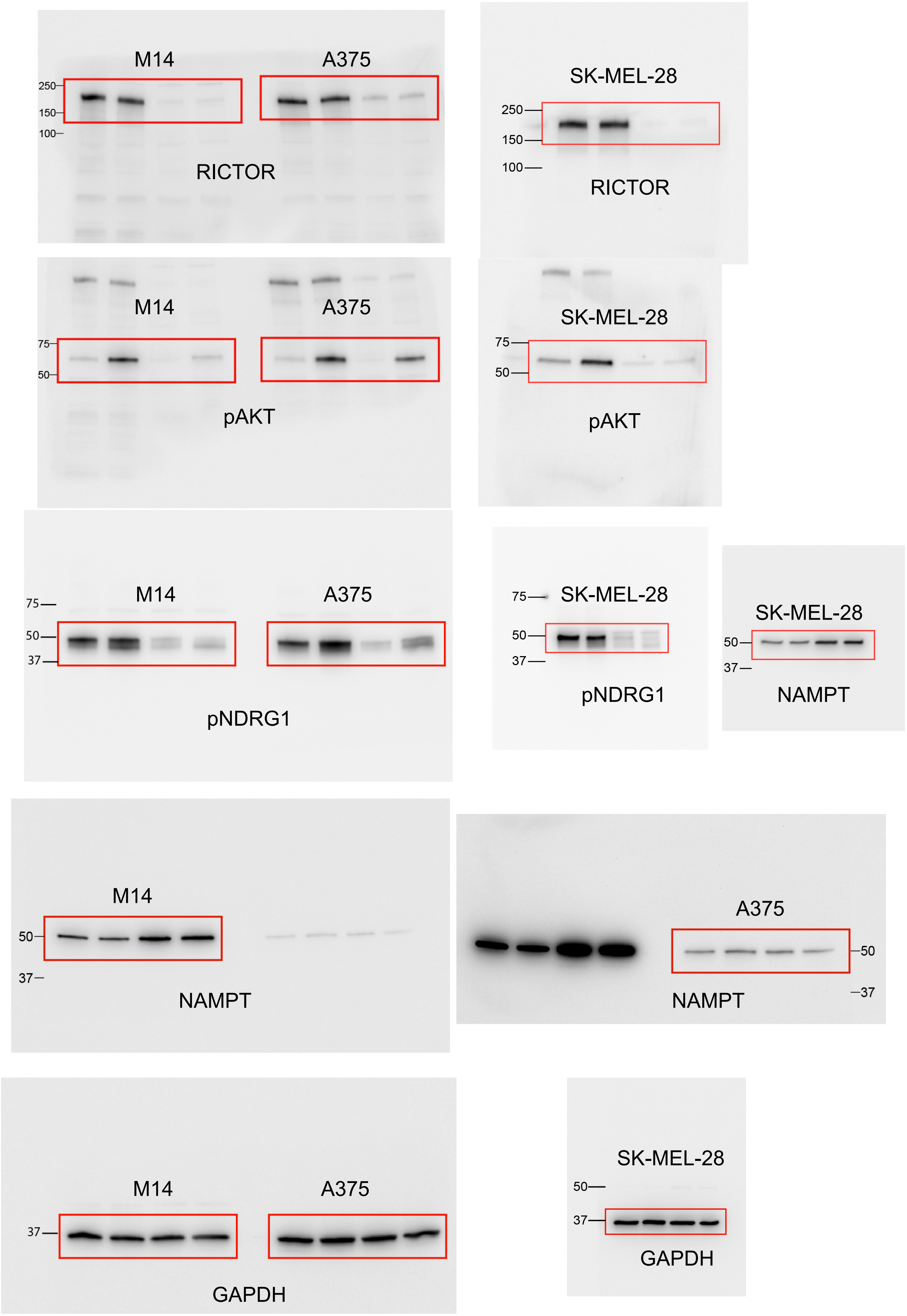
M14
A375
250
150
100
RICTOR
SK-MEL-28
250
150
100
RICTOR
M14
A375
75
50
pAKT
SK-MEL-28
75
50
pAKT
75
M14
A375
50
37
pNDRG1
75
SK-MEL-28
50
37
pNDRG1
SK-MEL-28
50
37
NAMPT
M14
50
37
NAMPT
A375
50
37
NAMPT
M14
A375
37
GAPDH
SK-MEL-28
50
37
GAPDH

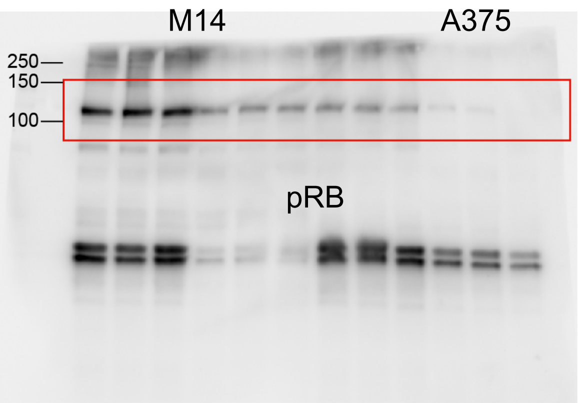
M14
A375
250
150
100
pRB

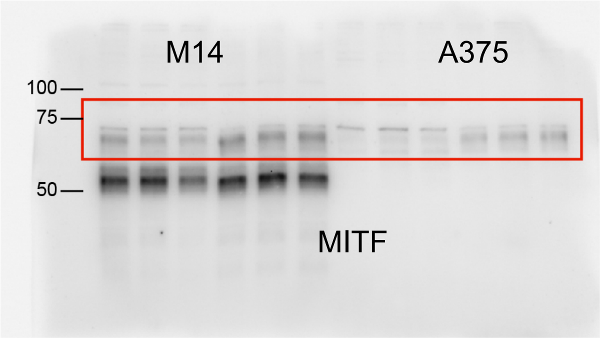
M14
A375
100
75
50
MITF

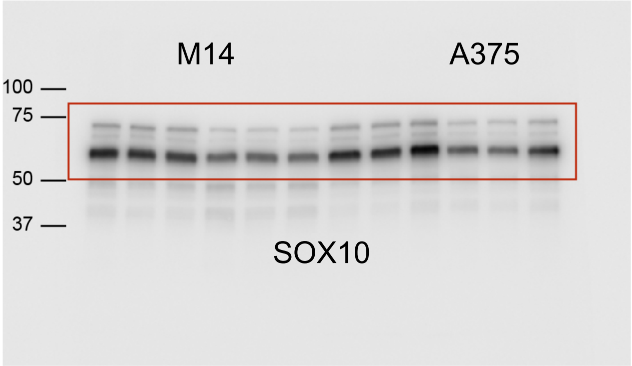
M14
A375
100
75
50
37
SOX10

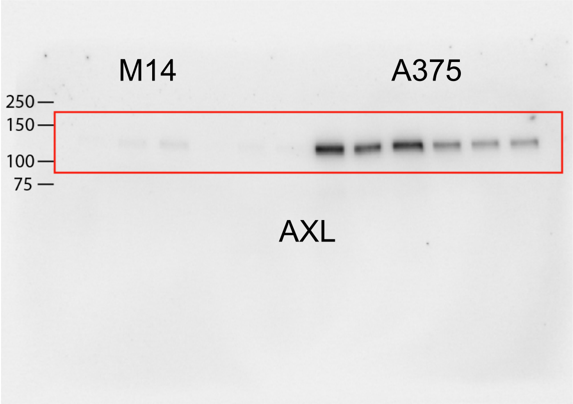
M14
A375
250
150
100
75
AXL
